# Climatic and soil factors explain the two-dimensional spectrum of global plant trait variation

**DOI:** 10.1038/s41559-021-01616-8

**Published:** 2021-12-23

**Authors:** Julia S. Joswig, Christian Wirth, Meredith C. Schuman, Jens Kattge, Björn Reu, Ian J. Wright, Sebastian D. Sippel, Nadja Rüger, Ronny Richter, Michael E. Schaepman, Peter M. van Bodegom, J. H. C. Cornelissen, Sandra Díaz, Wesley N. Hattingh, Koen Kramer, Frederic Lens, Ülo Niinemets, Peter B. Reich, Markus Reichstein, Christine Römermann, Franziska Schrodt, Madhur Anand, Michael Bahn, Chaeho Byun, Giandiego Campetella, Bruno E. L. Cerabolini, Joseph M. Craine, Andres Gonzalez-Melo, Alvaro G. Gutiérrez, Tianhua He, Pedro Higuchi, Hervé Jactel, Nathan J. B. Kraft, Vanessa Minden, Vladimir Onipchenko, Josep Peñuelas, Valério D. Pillar, Ênio Sosinski, Nadejda A. Soudzilovskaia, Evan Weiher, Miguel D. Mahecha

**Affiliations:** 1 Max-Planck-Institute for Biogeochemistry, Jena, Germany; 2 Remote Sensing Laboratories, Department of Geography, University of Zurich, Zurich, Switzerland; 3 German Centre for Integrative Biodiversity Research (iDiv), Leipzig, Germany; 4 Institute of Systematic Botany and Functional Biodiversity, University of Leipzig, Leipzig, Germany; 5 Department of Chemistry, University of Zurich, Zurich, Switzerland; 6 Escuela de Biología, Universidad Industrial de Santander, Bucaramanga, Colombia; 7 Department of Biological Sciences, Macquarie University, Sydney, New South Wales Australia; 8 Institute for Atmospheric and Climate Science, ETH Zurich, Zurich, Switzerland; 9 Norwegian Institute of Bioeconomy Research, Oslo, Norway; 10 Department of Economics, University of Leipzig, Leipzig, Germany; 11 Smithsonian Tropical Research Institute, Ancón, Panama; 12 Geoinformatics and Remote Sensing, Institute for Geography, University of Leipzig, Leipzig, Germany; 13 Environmental Biology Department, Institute of Environmental Sciences, CML, Leiden University, Leiden, the Netherlands; 14 Systems Ecology, Department of Ecological Science, Faculty of Science, Vrije Universiteit Amsterdam, Amsterdam, the Netherlands; 15 Instituto Multidisciplinario de Biología Vegetal (IMBIV), CONICET and FCEFyN, Universidad Nacional de Córdoba, Córdoba, Argentina; 16 Global Systems and Analytics, Nova Pioneer, Johannesburg, South Africa; 17 Chairgroup Forest Ecology and Forest Management, Wageningen University, Wageningen, the Netherlands; 18 Land Life Company, Amsterdam, the Netherlands; 19 Research Group Functional Traits, Naturalis Biodiversity Center, Leiden, the Netherlands; 20 Plant Sciences, Institute of Biology Leiden, Leiden University, Leiden, the Netherlands; 21 Estonian University of Life Sciences, Tartu, Estonia; 22 Department of Forest Resources, University of Minnesota, St Paul, MN USA; 23 Hawkesbury Institute for the Environment, Western Sydney University, Penrith, New South Wales Australia; 24 Institute for Global Change Biology and School for Environment and Sustainability, University of Michigan, Ann Arbor, MI USA; 25 Department of Plant Biodiversity, Institute of Ecology and Evolution, Friedrich-Schiller University, Jena, Germany; 26 School of Geography, University of Nottingham, Nottingham, UK; 27 School of Environmental Sciences, University of Guelph, Guelph, Canada; 28 Department of Ecology, University of Innsbruck, Innsbruck, Austria; 29 Department of Biological Sciences and Biotechnology, Andong National University, Andong, Korea; 30 Plant Diversity and Ecosystems Management Unit, School of Biosciences and Veterinary Medicine, University of Camerino, Camerino, Italy; 31 Department of Biotechnologies and Life Sciences (DBSV), University of Insubria, Varese, Italy; 32 Jonah Ventures LLC, Boulder, CO USA; 33 Facultad de Ciencias Naturales y Matemáticas, Universidad del Rosario, Bogotá, Colombia; 34 Departamento de Ciencias Ambientales y Recursos Naturales Renovables, Facultad de Ciencias Agronómicas, Universidad de Chile, Santiago, Chile; 35 School of Molecular and Life Sciences, Curtin University, Perth, Western Australia Australia; 36 College of Science, Health, Engineering and Education, Murdoch University, Murdoch, Western Australia Australia; 37 Department of Forestry, Universidade do Estado de Santa, Catarina, Lages, Brazil; 38 INRAE University Bordeaux, BIOGECO, Cestas, France; 39 Department of Ecology and Evolutionary Biology, University of California, Los Angeles, CA USA; 40 Department of Biology, Vrije Universiteit Brussel, Brussels, Belgium; 41 Landscape Ecology Group, Institute of Biology and Environmental Sciences, University of Oldenburg, Oldenburg, Germany; 42 Department of Ecology and Plant Geography, Moscow State Lomonosov University, Moscow, Russia; 43 CSIC, Global Ecology Unit CREAF-CSIC-UAB, Bellaterra, Spain; 44 CREAF, Cerdanyola del Vallés, Spain; 45 Department of Ecology, Universidade Federal do Rio Grande do Sul, Porto Alegre, Brazil; 46 Embrapa Recursos Genéticos e Biotecnologia, Brasília, Brazil; 47 Centre for Environmental Sciences, Hasselt University, Diepenbeek, Belgium; 48 Institute of Environmental Sciences, Leiden University, Leiden, the Netherlands; 49 Department of Biology, University of Wisconsin, Eau Claire, WI USA; 50 Remote Sensing Centre for Earth System Research, University of Leipzig, Leipzig, Germany; 51 Helmholtz Centre for Environmental Research, Leipzig, Germany

**Keywords:** Macroecology, Biogeography, Ecophysiology, Plant ecology

## Abstract

Plant functional traits can predict community assembly and ecosystem functioning and are thus widely used in global models of vegetation dynamics and land–climate feedbacks. Still, we lack a global understanding of how land and climate affect plant traits. A previous global analysis of six traits observed two main axes of variation: (1) size variation at the organ and plant level and (2) leaf economics balancing leaf persistence against plant growth potential. The orthogonality of these two axes suggests they are differently influenced by environmental drivers. We find that these axes persist in a global dataset of 17 traits across more than 20,000 species. We find a dominant joint effect of climate and soil on trait variation. Additional independent climate effects are also observed across most traits, whereas independent soil effects are almost exclusively observed for economics traits. Variation in size traits correlates well with a latitudinal gradient related to water or energy limitation. In contrast, variation in economics traits is better explained by interactions of climate with soil fertility. These findings have the potential to improve our understanding of biodiversity patterns and our predictions of climate change impacts on biogeochemical cycles.

## Abstract (web summary)

The authors investigate the broad-scale climatological and soil properties that co-vary with major axes of plant functional traits. They find that variation in plant size is attributed to latitudinal gradients in water or energy limitation, while variation in leaf economics traits is attributed to both climate and soil fertility including their interaction.

## Main

Plant functional traits have proved useful in identifying life history strategies^1,2^ for predicting plant community assembly^3,4^ and for assessing the impact of vegetation composition and diversity on ecosystem functioning^5,6^. Consequently, vegetation models including coupled climate–vegetation models benefit from a better representation of plant trait variation to adequately analyse terrestrial biosphere dynamics under global change^6,7^. Today, in combination with advanced gap-filling techniques^8^, databases of plant traits have sufficient coverage to allow quantitative analyses of plant form and function at the global scale^9^. Analysing six fundamental traits, Díaz and colleagues^10^ revealed that essential patterns of form and function across the plant kingdom can be captured by two main axes. The first reflects the size spectrum of whole plants and plant organs. The second axis corresponds to the ‘leaf economics spectrum’^11^ emerging from the necessity for plants to balance leaf persistence against plant growth potential. The concept of a global spectrum of plant form and function has since been investigated from various perspectives^12–14^. It has been shown, for instance, that orthogonal axes of variation in size and economics traits emerge even in the extreme tundra biome^13^ or at the scale of plant communities^12^. However, it remains unclear whether the two axes remain dominant for extended sets of traits or when differentiating among growth forms. A particular knowledge gap is what environmental controls determine these two axes of plant form and function.

There is ample evidence that large-scale variation of individual plant traits is related to environmental gradients. Early plant biogeographers suggested that climate and soils together shape plant form and function^15–17^ but could not propose a more precise theoretical framework describing these fundamental relationships. Over the last decades, examples have thus accumulated without an overall framework in which to place them^13,18,19^. For instance, tree height depends on water availability^20,21^ while leaf economics traits depend on soil properties, especially soil nutrient supply, as well as on climatic conditions reflected in precipitation^18,22,23^. Leaf size, leaf dark respiration rate, specific leaf area (SLA), leaf N and P concentration, seed size and wood density, all show broad-scale correlations with climate or soil^22,24–27^. It has also been reported that many of these traits show latitudinal patterns^24–27^. Generalizing such insights is, however, not trivial, as soil properties partly mirror climate gradients, as a consequence of long-term soil formation through weathering, leaching and accumulation of organic matter—processes related to temperature and precipitation^28^; however, climate-independent features reflecting geology and surface morphology also contribute to soil fertility^28^. Soil may furthermore buffer climate stresses; for example, by alleviating water deficit in periods of low precipitation^29^.

Combining the insights suggests that the global spectrum of plant traits reveals two internally correlated orthogonal groups and that many plant traits are individually linked to environmental gradients, we expect that both trait groups should closely follow gradients of climate and soil properties. Here, we investigate to what extent the major dimensions underpinning the global spectrum of plant form and function can be attributed to global gradients of climate and soil conditions; and to what extent these factors can jointly or independently explain the global spectrum of form and function.

**Extended Data Fig. 1:**
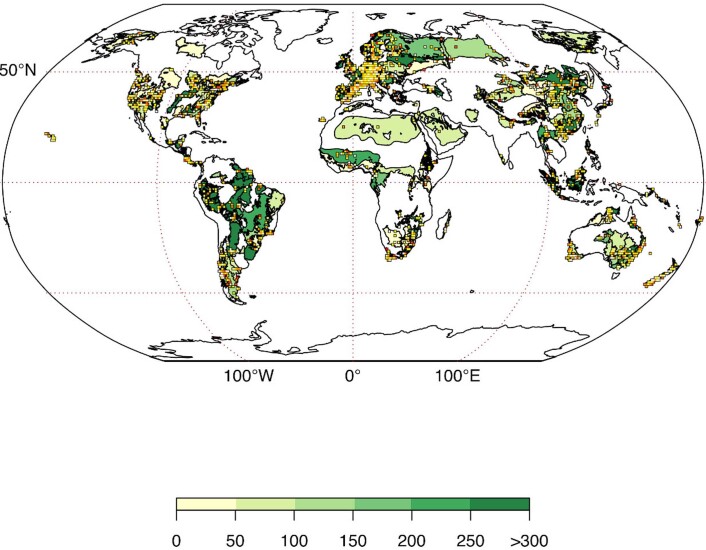
Map of Ecoregion data. Map of ecoregions (30) included in this study (n=220).

We compiled and analysed a dataset of 17 functional traits with a sufficient number of records in the TRY database^9^ to characterize the main ecoregions of the world^30^, that is, environmentally homogeneous areas with distinct biota (Extended Data Fig. 1). The dataset is based on 225,206 georeferenced observations comprising records of 20,655 species. The trait data were complemented with 21 climate variables and 107 soil variables (Methods; Supplementary Tables 1 and 2). Trait–environment relationships were analysed for species medians aggregated to ecoregions using ridge regression^31^, a robust method (Supplementary Figs. 1–3) suitable to deal with high-dimensional, unbalanced and collinear predictors in combination with hierarchical partitioning^32^ (Methods).

## Results

Our main analysis is based on median trait values of plant species per ecoregion. The rationale is that species presence indicates how the trait space can be realized in a given environment. Spatial aggregation is a suitable means to increase the detectability of global trait patterns (Supplementary Fig. 3), as described in earlier studies, where traits have been binned by temperature classes^33^ or for different altitudinal ranges^22^. Extreme outliers, for instance towering trees such as the Californian Sequoia (*Sequoiadendron sempervirens*), may still exist far away from the equator, where precipitation is sufficiently high^20^ but their influence is outweighed in our approach by an increasing fraction of small-statured herbaceous species from tropical to temperate and boreal regions.

### Orthogonal axes and trait clusters

**Fig. 1:**
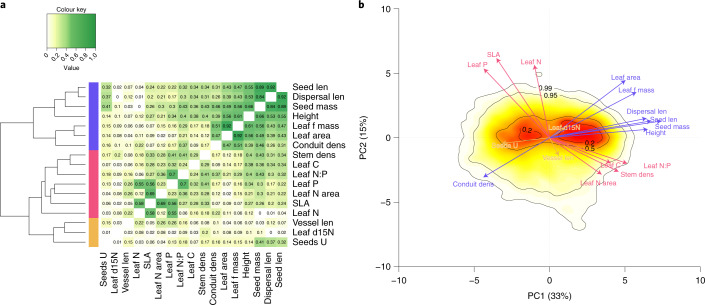
Previously identified global axes of variation in size and economics traits hold for an extended trait set (*n* = 36,197 species per ecoregion median). The set of 17 investigated traits (Supplementary Table 5) can be primarily divided into size and economics traits, which load differently onto the two PC axes describing their global distribution. **a**, Heatmap of covariation. Trait correlations are indicated using absolute Pearson correlation coefficients, with green shades indicating high absolute correlation and yellow shades indicating low absolute correlation. On the left, the distance tree of traits derived from hierarchical clustering is illustrated. Three resulting groups are: (1) size-related traits (blue) consisting of conduit density (conduit dens), leaf area, leaf fresh mass (leaf f mass), plant height (height), seed mass, dispersal unit length (dispersal len) and seed length (seed len); (2) economics traits (red) comprising SLA, leaf N content per area (leaf N area), leaf N, P and C concentrations, leaf N/P ratio (leaf N:P) and stem specific density (stem dens); and (3) a third (yellow) consisting of the number of seeds per reproduction unit (seeds U), leaf *δ*15N (leaf d15N) and vessel element length (vessel len). **b**, The first two PCs of the PCA. Arrow tips refer to the loading of the traits (Supplementary Fig. 6). Contour lines delineate the colour scale that corresponds to the kernel density of species (dense, red to sparse, light yellow; 20%, 50%, 95% and 99% of all species). PC1 explains 33% of trait variation and PC2 15% (Supplementary Fig. 5).

To understand whether the axes of variation identified for the grouping of six traits^10^ also hold for the extended set of 17 traits, we cluster their trait–trait correlations (Fig. 1a and Supplementary Fig. 4) and further represent these relations on the basis of their principal components (PCA; Methods). This analysis supports the clear distinction of size versus economics traits identified by Díaz and colleagues^10^. The group of size traits contains two subclusters. The first includes height and seed size traits: plant height (height), seed mass, seed length and dispersal unit length (dispersal length). The second subset contains traits that are linked through plant hydraulic scaling relationships^34^ and contrasts high conduit density (that is, number of conduits per sapwood cross-sectional area) with high leaf area and leaf fresh mass (leaf f mass). Economics traits represent dry mass and nutrient investments in plant tissues, and the rate and duration of returns on those investments^11^. They are represented by leaf nitrogen content per leaf area (leaf N area), leaf nitrogen (leaf N), phosphorus (leaf P) and carbon (leaf C) content per dry mass, leaf N to P ratio (leaf N:P) and SLA. Stem specific density (stem density) takes an intermediate position (Fig. 1b) but more closely clusters with this set of economics traits (Fig. 1a), suggesting a syndrome of traits promoting slow to fast nutrient and carbon processing at the whole-plant level^35–37^. Furthermore, we identify a third group of traits that appear to be only weakly correlated with any other trait. This third group contains seed number per reproduction unit (seeds U), leaf *δ*15N (leaf d15N) and vessel element length (vessel length). The first two principal components (PC) of the PCA on the trait data represent 48% of the overall variation (Supplementary Fig. 5). PC1 is determined by size traits and accounts for 33% of the variance; PC2 is determined by economics traits and accounts for 15% of the variance (Fig. 1b and Supplementary Fig. 6). These two main axes remain clearly identifiable when the analysis is conducted separately for woody and non-woody species (Supplementary Figs. 7 and 8). The remaining PCs each account for less than 10% of variance (PC3 = 9.36%). In the following, we focus on the two groups of size and economics traits (Supplementary Fig. 5).

### Latitudinal trait variation

**Fig. 2:**
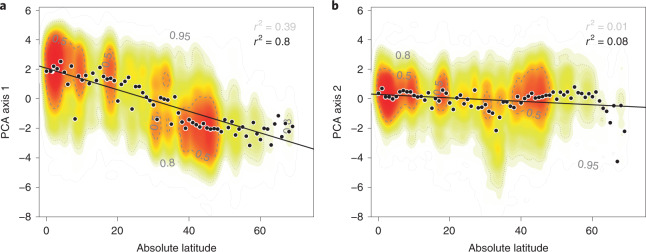
Size traits, not economics traits vary with latitude: the PC1 of the PCA on 17 plant traits shows a clear latitudinal gradient while PC2 does not (*n* = 36,197 species per ecoregion median). Contour lines delineate the colour scale that corresponds to the kernel density of species (dense, red to sparse, light yellow; 5%, 95%, 99% quantiles). Mean estimates aggregated at 1^°^ absolute latitude are indicated as black dots. The line refers to a linear model (ordinary least squares). **a**, PC1 representing mainly size traits (conduit density, leaf area, leaf fresh mass, plant height, seed mass, dispersal unit length, seed length) regressed against absolute latitude. Linear model: *r*^2^ = 0.38 without bins; *r*^2^ = 0.84 aggregated at 1^°^ absolute latitude. **b**, PC2 representing mainly economics traits (leaf N, leaf N per area, leaf P, leaf N:P ratio, SLA, leaf C, stem density) regressed against absolute latitude. Linear model: *r*^2^ = 0.01 without bins; *r*^2^ = 0.08 aggregated at 1^°^ absolute latitude.

As an investigation of broad-scale gradients among size and economics traits, we analyse latitudinal gradients of the first (PC1) and second (PC2) principal components. PC1—representing primarily size-related traits—shows a strong linear latitudinal signal (on the basis of species: *r*^2^(PC1) = 0.37, at the ecoregion level *r*^2^(PC1_aggregated_) = 0.84; Fig. 2a). By contrast, the axis representing primarily economic traits, PC2, shows little response to latitude (on the basis of species: *r*^2^(PC2) = 0.01, at the ecoregion level *r*^2^(PC2_aggregated_) = 0.08; Fig. 2b, for woody non-woody species Supplementary Fig. 9), except for a dip at 35^°^ and declining sharply at 60^°^ where the species density also drops (but see Supplementary Fig. 10 for comparison to an independent dataset from arctic latitudes which shows the same pattern). Latitudinal gradients are known to be strongly related to climate, due to the distribution of solar energy and general atmospheric circulation patterns. Therefore, we propose that those climate (and soil) aspects that co-vary with latitude consistently determine size traits, while they have little effect on economics traits, which are more strongly affected by latitude-independent soil (and climate) effects (Supplementary Fig. 11).

### Climate and soil: joint and independent effects

**Fig. 3:**
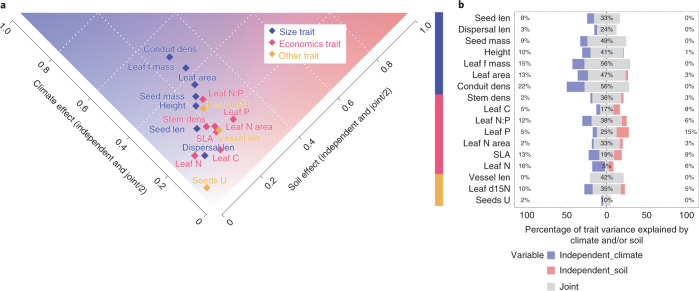
Climate and soil variables explain up to 77% of variance in size and economics traits. Hierarchical partitioning^32^ identifies the contribution of climate and soil variables to explain each trait (*n* = 220, ecoregional median trait: blue, size; red, economics; yellow, other). The joint effect is the fraction explained by both climate and soil together, and is split equally among them. The independent effect is the fraction of *r*^2^ explained exclusively by either soil or climate variables. a, Tilted *x*–*y* plot of the soil versus climate variables to explain a trait. The axes show the sum of the respective joint and independent effect (hierarchical partitioning). The colours reflect the strength of: the independent effect of climate plus its share of the joint effect (*r*^2^; purple); and soils' independent effect plus its share of the joint effect (peach). The sum of both axes equals the total *r*^2^ explained by climate and soil; in cases where soil showed a negative independent effect only the climate-independent effect is shown (and vice versa but see Table 1). b, Percentage variation explained by climate (purple, percentages on the left), soil (peach, percentages on the right) and jointly (grey, percentages in the middle) for trait groups— size, economics and other. Total bar length = total *r*^2^ explained by climate and soil; in cases where soil showed a negative independent effect only the climate-independent effect is shown (and vice versa but see Table 1). For leaf area, climate and soil jointly explain 47%, the independent climate effect explains an additional 13% of the variance, while soil explains 3%, totalling 63% of variance explained. For trait abbreviations see Fig. 1.

The differences in latitudinal relationships between the two PC axes support the hypothesis that different environmental factors should drive variation within the separate groups of size versus economics traits. We assess the joint and independent effects of climate and soil on trait variability (ridge regression, RR; Table 1 and Fig. 3). Overall, size traits are better explained (RR; *r*^2^ = 0.55; maximum *r*^2^ = 0.78 for conduit density; Table 1) than are economics traits (RR; *r*^2^ = 0.40; maximum *r*^2^ = 0.55 for leaf N:P ratio; Table 1). We find a substantial joint effect of climate and soil variables—in every case larger than either unique effect—which reflects strong interactions between specific climate and soil predictors (RR with hierarchical partitioning (HP); Fig. 3b and Supplementary Fig. 12). However, we also observe independent effects of climate and soil (RR with HP; Fig. 3 and Table 1). The independent climate effects are observed across traits but size traits tend to be better explained by the independent climate effects than are economics traits. In contrast, independent soil predictors are relevant for all economics traits but not size traits (apart from a small contribution to leaf area). We interpret these results as evidence for the importance of both joint and independent effects of climate and soil variables for whole-plant strategies^2,37,38^ which we show here at the global scale along with a dichotomous tendency of a stronger imprint of climate factors on size traits and of soil conditions on economics traits (Fig. 3, Supplementary Figs. 13–38 and Supplementary Table 3). We propose that the dominance of joint effects implies that interactions between soil and climate properties are of primary importance in plant trait ecology; as opposed to trait syndromes being defined by single environmental variables in isolation.

**Table 1:** Showing for each trait the variance explained (*r*^2^) by ridge regression models for 220 ecoregions and the independent effects for climate and soil listed from hierarchical partitioning that, respectively, add up with the joint effect to the variance explained by climate or soil

Trait^a^	Group	Explained variance^b^ by soil and climate (*r*^2^)	Soil (independent effect^b^) (*r*^2^)	Climate (independent effect^b^) (*r*^2^)	Joint effect^b^ (*r*^2^)
		Ridge regression model	Hierarchical partitioning	Hierarchical partitioning	Hierarchical partitioning
Seed length	Size	0.4	–0.01	0.08	0.33
Dispersal length	Size	0.26	–0.01	0.03	0.24
Seed mass	Size	0.57	–0.01	0.09	0.49
Height	Size	0.52	0.01	0.1	0.41
Leaf f mass	Size	0.72	0	0.15	0.56
Leaf area	Size	0.63	0.03	0.13	0.47
Conduit density	Size	0.77	–0.01	0.22	0.56
Stem density	Economics	0.41	0.03	0.02	0.36
Leaf C	Economics	0.29	0.08	0.05	0.17
Leaf N:P	Economics	0.55	0.06	0.12	0.38
Leaf P	Economics	0.45	0.15	0.05	0.25
Leaf N area	Economics	0.39	0.03	0.02	0.33
SLA	Economics	0.41	0.09	0.13	0.19
Leaf N	Economics	0.26	0.06	0.16	0.05
Vessel length	Other	0.4	0	–0.03	0.42
Leaf d15N	Other	0.51	0.05	0.1	0.35
Seeds U	Other	0.1	–0.02	0.02	0.1

^a^For full versions of traits, see main text. Mean values; minimum and maximum values from different cross-validation runs in Supplementary Table 8. Negative values indicate a reduction of explained variance when respective variables are added to the ridge regression model.

**Fig. 4:**
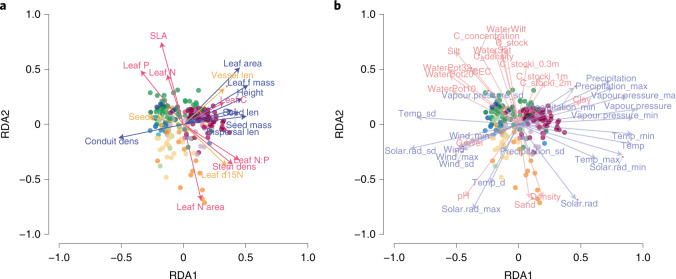
RDA of traits reveals the relationships of climate and soil factors associated with trait distributions (*n* = 220, ecoregion median, only topsoil layer variables included; variance explained: RDA1 = 63%, RDA2 = 18%). **a**,**b**, The output of the RDA is split into two plots: traits (**a**), where arrows are coloured according to trait groups (blue, size traits; red, economics traits; yellow, other traits; arrow length and point positions scaled to fit the plot); and environmental factors (**b**), where arrows are coloured according to predictor group (climate, blue and soil, red variables; arrow length and point positions scaled to fit the plot). In **a** and **b**, points represent ecoregions and are coloured according to biome (red, tropics; green, temperate; yellow, desert; orange, Mediterranean; dark blue, tundra). Climate variable abbreviations are composed of the variable (average if not stated differently) and a suffix. Variables are: Solar.rad, solar radiation; Vapour.pressure, vapour pressure; Wind, average wind speed; Temp, temperature; Precipitation, precipitation. Suffixes are: no suffix, mean of respective variable; d, diurnal range; min, annual minimum of the respective variable; sd, seasonality of respective variable. Soil variable abbreviations (all topsoil) are: Density, soil density (kg/m^3^); pH, pH value; Sand, sand fraction (vol%); Silt, silt fraction (vol%); Clay, clay fraction (vol%); C_concentration, organic carbon concentration; C_density, organic carbon density; CEC, cation exchange capacity; C_stock, soil organic carbon stock at depth 0.00 m; C_stocki, organic carbon stock for depth intervals—0.3m (0–0.30 m), 1m (0–1 m), 2m (0–2 m); WaterWilt, available soil water capacity (volumetric fraction) until wilting point; WaterSat, saturated water content; WaterPot, available soil water capacity for moisture potentials—10 (–10 kPa;pF 2.0), 20 (–20 kPa;pF 2.3), 32 (–31.6 kPa;pF 2.5). For trait abbreviations see Fig. 1.

We next ask how the climate and soil datasets are interdependent and which predictors add the most relevant information. For this purpose, we related all traits to environmental variables in a redundancy analysis (RDA; Methods; Fig. 4). The RDA again identifies two main axes of size and economics traits (Fig. 4a), which are now shown together with the environmental variables that co-vary linearly with those traits (Fig. 4b and Supplementary Fig. 39). The first RDA axis corresponds to size traits (Fig. 4a) and represents an axis of water and energy (for example, precipitation, vapour pressure and temperature; Fig. 4b). Two attributes of soil texture important for water retention—the fraction of gravel and clay—also vary along this axis. The second RDA axis corresponds to economics traits (Fig. 4a) co-varying with an axis of soil variables generally associated with soil fertility (that is, soil texture (silt versus sand), water holding capacity, carbon concentration and stocks), as well as the climate variable mean solar radiation (Fig. 4b).

## Discussion

This study shows that the proposed global spectrum of plant form and function fits well to a substantially extended trait space compared to the original study^10^, with seven traits that capture the whole-plant size spectrum and seven traits that capture the leaf economic spectrum and only three traits that do not fall along these dimensions (Fig. 1b). One explanation could be that the varying fraction of woody and non-woody species would drive these patterns. However, we showed that these two main trait groups remain clearly identifiable when the analysis is conducted separately, yet with fewer samples, for woody and non-woody species (Supplementary Fig. 8).

However, we cannot discard the possibility that additional traits may add relevant axes of trait variation. For example, our study does not include carbon fixation rates^39^ or fire adaptation traits^40^, nor does it include any root traits—representing an essential gap to be filled at the global scale^41^. The respective data are too scarce to yet be integrated with global datasets. If such data were available they would have the potential to fundamentally change our perception of global plant form and function, and their relation to ecosystem functioning.

Variation in size traits, represented by PC1 in Fig. 1b, shows a clear latitudinal gradient (Fig. 2b). In contrast, variation in economics traits (represented by PC2) does not show a latitudinal trend. Only a dip is apparent at around 35^°^ (absolute), in addition to a decrease at high latitudes above 60^°^ (absolute) where available data become increasingly limited. However, comparison to a recent arctic dataset indicates that this decrease in variation at high latitudes reflects available observations (see Supplementary Fig. 10 for a comparison to independent data). These patterns might represent a response to nutrient limitation and drought^42,43^ in water-scarce and nutrient-scarce deserts and Mediterranean regions (Supplementary Fig. 40) or boreal and arctic areas characterized by short growing periods slowing down mineralization. The dip at ~35^°^ indeed can be related to low water availability (Supplementary Fig. 41). At high latitudes, cold winters and short growing seasons constrain plant height^13^ and require on average more conservative nutrient-use strategies (like evergreen leaves) and protection against frost damage than the global mean, despite the high functional diversity in economics traits observed at these latitudes^13^. Additional datasets may shed more light on specific conditions, for example see Bjorkman et al.^19^. Future studies should quantify how individual stressors, for example radiative stress or water stress, relate to global patterns of trait variation.

The climate and soil factors used in this analysis explain up to 77% of observed trait variation—a high fraction given that trait variation is widely known to be determined also by other factors such as biotic interactions (for example, soil biota) and anthropogenic effects or disturbances and local effects such as those of microclimate^12,44–46^. Recent findings on how different trait groups vary with the environment indicate that size and economics traits vary differently^13^ and in particular respond differently to climate and soil^19^.

Our analyses reveal a dominant joint effect of climate and soil drivers on trait variation—as already suggested by a number of earlier studies^18,19,22^ but not yet quantified globally.

The orthogonality of the two main dimensions of plant trait variation suggests that different aspects of climate and soil variables are relevant to explain plant trait patterns at the global scale (Supplementary Figs. 11–39). While latitude-related variables (mainly climate) explain size traits, variables that share less explanatory power with latitude (mainly soil) explain economics traits (Supplementary Table 4 and Supplementary Fig. 11). The RDA presented in Fig. 4 (Supplementary Fig. 39) provides some insight on the nature of these climate–soil interactions. The first RDA axis, which describes variation in size traits, resembles a latitudinal gradient. On one extreme end, ample water supply from high and frequent precipitation, abundant water vapour and constant rates of high solar radiation meet the fundamental requirements of plant physiology—water, sunlight and warm temperatures. Additionally, these conditions promote weathering of soil minerals but also microbial activity, contributing to fast turnover rates of organic matter supporting nutrient provisioning^28,47^; in brief, they represent conditions that allow plants to grow fast and tall in the race for light. Large vessels supporting large leaves promote high rates of water transport and thus growth, which is only possible because of the small risk of embolism under these benign water conditions^43^. The high carbon gains can be invested in large fruits and seeds (seed mass, seed length and dispersal unit length). Further along this gradient, the above-mentioned plant requirements become limited: water supply and temperatures are reduced and slow metabolic rates aboveground and belowground. In ecoregions of the boreal and desert biomes, conduit diameter is constrained by the risk of cavitation during freeze–thaw cycles^43^ and water scarcity, amplified by little water holding capacity of gravel-rich soils. Our analysis thus indicates that size traits appear to be related to a latitudinal gradient of climatic favorability for plant growth determined by water and light availability.

Important correlates of water and nutrient availability are associated with the second RDA axis, describing variation in economics traits. Traits associated with an acquisitive strategy are related to indicators of soil fertility, most importantly silt and organic matter concentration as well as pH (refs. ^18,28^). Soil pH is intermediate between the two axes, as might be expected given that pH reflects both broad-scale climate variation (especially aridity^47^) and a variety of processes related to nutrient availability and soil microbial communities^18,48–50^. Silt forms the substrate of our most fertile soils as its structure is able to retain water against gravitation (unlike sand) but renders it accessible to plants under drought conditions^28,51^ (unlike clay). The high fertility is associated with a high concentration of organic matter, which has a high cation exchange capacity especially under high pH (ref. ^47^). On the opposite end of the gradient, sandy soils require adaptations to both water and nutrient limitation. The trait configuration at the conservative end of the economics traits (low SLA, high tissue density and high organ longevity) represents an adaptation to both^11,37^. Various processes exist that lead to variation in the soil characteristics underlying the second RDA axis independent of latitude^18^—for example, sandstone as a geological substrate giving rise to sandy soils exists from the tropics to the arctic^28,51^. However, different climate variables related to solar radiation, temperature and precipitation, which influence long- and short-term soil development processes directly and indirectly via soil biology^28,51,52^, are related to this axis. Variation in economic traits is most probably the evolutionary response to exploiting this partly climate-independent edaphic niche axis.

Size traits are on average explained better than economics traits by the environmental variables considered in this study. The lower fraction of explained variance for economics traits could have several causes. Firstly, data on soil factors that are likely to be very important, such as soil nitrogen and phosphorus availability^18,23^, are not yet available at a global scale. Secondly, economics traits show relatively more within-site variation than across-site variation in comparison to size traits (Supplementary Fig. 42), probably because economics traits vary more than size traits within one plant; for example, leaf N per area and SLA vary with age and light availability^53^. Thirdly, soil heterogeneity within ecoregions—both abiotic and biotic—may weaken the relationship between economics traits and environmental variables^12,54,55^. Reasons for small-scale soil variation are, for example, topography, soil age and thus fertility^56^ but also abundance of microbial communities and mycorrhiza that interact with climate, pH, soil properties and also plant traits^50^. Trait–environment relationships due to smaller scale variation require well-resolved soil data. However, we note that soil physics and chemistry explain a large portion of variance along the trait PC axis three (which itself explains slightly less than 10% of variance in the PCA (9.36%); Supplementary Figs. 5, 6 and 38). We expect that with improved soil datasets and a higher resolution, the joint control of climate and soil on trait variation will probably appear even stronger and more evenly distributed between the two groups of driver variables.

Our analysis can serve as reference for model developments that increasingly consider plant functional traits as part of vegetation dynamics under climate change^44^.

Individual plants and their trait syndromes are considered to be viable only within specific environmental conditions^2^. Therefore trait–environment relationships should be scale-independent. However, different plant strategies can be successful under given environmental conditions, which in addition are often confounded by small-scale variation. In analyses to date, trait–environment relationships become more apparent for aggregations higher than the community scale^12^, where most of the small-scale variation is averaged out. In addition the difference between potential and actual vegetation is suggested to explain some of this gap^13^. Dynamic global vegetation models predict individual plant processes well but fail to produce reliable forecasts with a changing environment^44^. Deciphering at which spatial and temporal scale, or conditions, actual vegetation is representative of potential vegetation may advance our understanding of community assembly and necessary model complexity.

Trait–environment correlations identified in our study should not be confounded with causality. Yet, the ubiquitous importance of climate variables for explaining current differences in trait expression at ecoregion scale, suggests that trait shifts will occur with climate change. Trait shifts are constrained by available trait combinations in addition to other constraints such as species dispersal. For example, our results indicate that plant size increases with temperature so long as sufficient water is available (Fig. 4 and Supplementary Figs. 19, 20 and 21), in line with the finding that species become larger and large species are more prevalent at warmer and wetter sites in the tundra^19^. Global change is also reflected by soil degradation. Changes in soil parameters can be considered to also correspond with trait shifts, especially for economics traits. Human-induced soil degradation has many facets: often fertile topsoil is lost or toxic substances accumulate; rooting is impeded and altered by artificial fertilizers; while soil formation takes millenia^57^. The trait shifts may thus be similarly complex and depend on the extent and type of soil degradation. For example, in areas of wind and water erosion, species that tolerate lower nutrient availability may be more successful and this may be reflected in lower leaf nutrient contents (Fig. 4 and Supplementary Fig. 30). The fertilization of nutrient-poor grasslands, for example resulting from agricultural run-off, may shift these areas from more conservative to more competitive species with higher leaf nutrient contents.

Plants as a whole need to balance both size and economics traits. To sustain human livelihoods, it may be important to understand the local expression of trait shifts and their global consequences for biodiversity when viable trait combinations change.

In conclusion, the insights extracted here advance our understanding of broad-scale plant functional patterns. In particular, we highlight the combination of independent and particularly joint effects of climate and soil on trait variation, an interaction that has to date been neglected because few studies include both in a single analysis, at the global scale as we have done here. In doing so, we identify an important gap in knowledge: what is the nature of climate–soil interactions that drive whole-plant trait variation and what distinguishes the majority of climate and soil factors having joint effects on plant traits from those with independent effects? These are the sorts of questions that require answers to increase our capacity to predict plant functional diversity in a changing environment. Such predictive power would contribute to a sound basis for assessing long-term feedbacks between global environmental change and the terrestrial biosphere, helping to constrain parameters of global coupled climate–vegetation models. Humans are currently modifying both climatic and edaphic conditions at the global scale. Climate envelope models used to predict vegetation shifts must be complemented by drivers related to large-scale anthropogenic alterations of soil conditions resulting, for example, from land-use change, atmospheric nitrogen deposition, fertilization, liming and salinization. Our global analysis provides an essential context for finer-scale studies to directly tackle questions of biological processes and mechanisms at landscape and community scales.

## Methods

We extracted data on 17 plant functional traits from a gap-filled version of TRY database^9^ (Supplementary Table 5; www.try-db.org, accession date July 2017, request no. 3282) which includes published literature^11,58–101,101–310^. Quality control was conducted according to the published protocol of TRY.^9^, ^311^ Traits with *z*-score > 4 were excluded and those with *z*-score > 3 were checked for plausibility. Before this, missing data were imputed using a Bayesian hierarchical probabilistic matrix factorization (BHPMF) algorithm^8,312^ for an extended dataset, derived from TRY (Supplementary Table 6). Imputation was done to be able to include the maximum number of species in our analyses. Then the 17 traits were selected among the traits with the largest total number of entries. The data were attributed to ecoregions^30^ (Supplementary Table 7 and Extended Data Fig. 1) and aggregated to species median values. The imputed values were calculated using the whole dataset at the individual record level. BHPMF calculates the imputations from 1,000 Gibbs sampler (Markov chain Monte Carlo) imputations by taking the mean of every twentieth imputation of these 1,000 ‘versions’, after the first 200 are removed. Then the species median was calculated at the ecoregion level. We excluded observations that were not georeferenced because we could not attribute them to ecoregions. According to TRY regulations, data from experimental treatments (for example, fertilization) or from botanic gardens were also excluded. In total, we included 225,206 observations from 20,655 global unique species (36,197 unique species to ecoregion combinations). Throughout this study we used one of two aggregation levels: either species median per ecoregion (ER)^30^ resulting in unique species values per ecoregion (termed A1, *n* = 36,197 with *n* = 20,655 globally unique species) or the aggregation to median ecoregions calculated from median species per ecoregion (termed A2, *n* = 220). R was used for all analyses and figures^313^.

### Hierarchical probabilistic matrix factorization

#### Description

BHPMF decomposes or factorizes probabilistically a matrix (probabilistic matrix factorization, PMF^314^) using information contained within different hierarchical levels (here, taxonomy) within a Bayesian framework^8^. The underlying premise of BHPMF is to gap-fill (or more accurately, to predict) traits of an individual plant using trait–trait correlations as well as intraspecific and interspecific trait variability.^8^. Using a Gibbs sampler (a Markov Chain Monte Carlo algorithm), BHPMF also provides a prediction confidence in the form of standard deviations which is a per-value estimate of uncertainty in trait predictions^8^. BHPMF can fill gaps if there is at least one value per row (species) and column (trait).

#### Implementation

The largest possible dataset was retrieved at the time when study was conducted, including 172 traits of 652,957 individuals (Supplementary Table 6). For data preparation before BHPMF, all individual-level trait data were firstly log-transformed and secondly normalized via zlog transformation ($z=\frac{x-\bar{x}}{\mathrm{s.d.}}$). Log transformation was chosen to achieve a closer-to-normal distribution of values per trait^311^,^313^. This transformation is considered necessary because a given difference for small trait values (absolute value) is likely to be physiologically more relevant than the same difference (absolute value) for large trait values.

BHPMF internally splits the datasets randomly into a training dataset (80%), a test dataset (10%) and a validation dataset (10%).

The training dataset is used during training of latent vectors, while the test data are tested against to improve the latent vectors, and finally the validation dataset serves as the basis for calculation of the root mean square error (RMSE) and stopping the optimization of latent vectors within BHPMF^8^. The validation dataset ensures ongoing amelioration of the model performance during the training process and stops the process after five consecutive iterations with stable RMSE. The test dataset is used only on the lowest taxonomic level (individuals × traits). BHPMF was run with a maximum of 1,000 iterations, whereas the first 200 were discarded during the ‘burn-in’ phase, as predictions of these iterations are likely to be influenced by the initialization of BHPMF rather than being part of the probability density distribution to be sampled by BHPMF. To avoid autocorrelation, only every twentieth iteration was used to calculate the resulting trait values. The mean of these predictions result in the final trait values used as the output. Compared to the original data, the imputed values are similar in terms of trait–trait correlation, according to the Procrustes test provided in ref. ^10^.

### Trait clustering

To define groups of correlated traits, we clustered species’ traits (species median per ecoregion, A1) on the basis of absolute pairwise Pearson correlation coefficients using a hierarchical clustering algorithm (‘complete linkage clustering’). Variables were transformed into distances previous to the clustering. Hierarchical clustering then attributes variables (here, traits) to groups of least distance and highest similarity. Traits were more like each other if they exhibited similar correlation patterns with all other traits. We set a distance between traits of 1 as the threshold for defining trait clusters. We used the R package ‘stats’ function ‘hclust’ included in R (ref. ^315^).

### PCA

Values for all 17 traits (unique species per ecoregion, A1) were natural log-transformed and then projected onto components (PCA). We used the R package FactoMineR^316^ that scales data internally. After the PCA (A1), we extracted the variance explained (Fig. 1b and Supplementary Fig. 5) and respective loadings for the first five principle components (Fig. 1b and Supplementary Fig. 6), which are significant according to the number of axes to keep estimated using a sequential Bonferroni procedure (R package ade4 (refs. ^317–321^), function testdim). For the analysis (ridge regression package ‘glmnet’^322,323^) for Fig. 3, all environmental variables (climate and soil) were first reduced with this package to 20 PCs.

### Environmental variables

To represent climate conditions we used 21 variables derived from WorldClim at a resolution of 1 km for temperature, precipitation, vapour pressure, solar radiation and wind (Supplementary Table 1). To characterize soil conditions we used 107 variables derived from the ISRIC data product ‘SoilGrids’^324–326^ (https://soilgrids.org/ through ISRIC—WDC Soils). ‘SoilGrids’ provides global predictions of 17 fundamentally different soil characteristics (some for seven depths, that is 0, 5, 15, 30, 60, 100, 200 cm; Supplementary Table 2) at a resolution of 1 km. SoilGrids are publicly accessible environmental data (Creative Commons Attribution 4.0 International), with a collection of georeferenced soil profile data and are managed in World Soil Information Service^324^.

### Aggregation of traits and environmental variables to ecoregions

To determine trait–environment relationships, we aggregated trait as well as environmental data to regions, here ecoregions^30^ (ecoregion aggregation A2, see also above; Supplementary Table 7). Ecoregions are environmentally homogeneous areas, nested within biogeographic realms (defined by refs. ^327,328^) and biomes (modified after refs. ^329,330^ but see ref. ^30^). As a first estimate, ecoregions are distinct biotas^328,331^ defined by the physiognomy of the prevailing climatic climax vegetation^30^. These areas of distinct biotas, areas of relatively uniform flora or fauna, are next subset into provinces with substantial differences of vegetation on the basis of a selection of plants and animals, maps and expert knowledge^30,331^. At global scale Olson et al.^30^ defined 867 ecoregions. Ecoregions were chosen as the scale of aggregation for their high signal-to-noise ratio and the ability to correct for sampling bias. While the grid scale has higher spatial resolution, it lacks estimates of species richness (equivalent of Kier species richness^174^) and is not as well explained by the climate and soil (Supplementary Fig. 3) and distribution of grids is globally uneven (Extended Data Fig. 1) in comparison to ecoregions. The global sampling distribution is recognized to show a bias towards Europe^9^, which is even more pronounced in the lower level data (grid scale) than in the more aggregated one (Extended Data Fig. 1). Our method accounts for this oversampling and reproduces a stable pattern, even when species in oversampled ecoregions are deleted (Supplementary Fig. 2).

For each of the 867 ecoregions, we calculated the median ecoregion aggregate trait value from the median trait values of all species identified in each region. For further analyses, we only used regions with >20 species and a representation of >1% of the estimated species richness of the ecoregion^30^. Preliminary tests with different selection criteria (for example, number of species and inclusion or exclusion of 1% of species richness estimate by Kier et al.^174^) showed that lower numbers of species per ecoregion result in weaker explained variance, while stricter rules reduced the number of ecoregions. These selection criteria serve as a quality control because ecoregions with poor representation of species richness are excluded, as we can expect the regression to the mean to be stronger with more species data. A total of 220 ecoregions met these criteria and were included in the analysis. On average, these ecoregion-level trait values were based on 164 species-level trait medians (with a maximum of 1,245 species in Tapajós-Xingu moist forests; Supplementary Table 7). In total, we aggregated 36,197 median species trait values to ecoregions. These ecoregions cover the global latitudinal gradient (Fig. 2) as well as a substantial fraction of the geographic space (Extended Data Fig. 1). To aggregate environmental variables to ecoregions, we associated each trait observation with its corresponding values of climate and soil variables. Then, we averaged over all values within one ecoregion. Thus, the selected environmental variables represent averages that are weighted by the number and locations of trait observations within ecoregions.

### Model building

Ecoregion trait values (natural log-transformed, A2) were related to all environmental variables using ridge regression^31^, which is a well-established linear regression method that is suitable to deal with a large number of collinear predictors and uneven numbers of predictors for climate and soil. We used the R package ‘glmnet’^322,323^. From aggregating trait values to ecoregion medians we obtain 220 samples for each trait. The environmental predictors of climate and soil were reduced to 20 each by means of a PCA. In addition, the environmental predictors show relatively high collinearity, thus duplicated information. Ridge regression addresses collinearity among predictors by shrinking (regularizing) regression coefficients according to a penalty on the L2 norm of the vector of regression coefficients. The regularization parameter lambda was obtained via tenfold cross validation. The variance explained was derived from an iterative holdout set (tenfold cross validation), that is, prediction of 90% of randomly sampled ecoregions for inclusion in model building and then predicting the remaining 10% of the data to evaluate the quality of the models. The final model predicts the remaining 10% of unused ecoregions. This prediction-loop was repeated until all ER trait values are predicted, that is, resulting in different linear models. Repeated *r*^2^: the *r*^2^ is the squared correlation of predicted versus original ER trait values. This procedure was repeated 50 times and the explained variances’ (*r*^2^) mean, minimum and maximum were calculated. For the purpose of defining how much of the explained variance is due to independent and joint information in the data streams, we used hierarchical partitioning^32^. Model outputs (*r*^2^) of all repetitions (*n* = 50, if not indicated differently) were used as input (ridge regression, partial least squares (PLS) with and without PCA, random forest).

### Redundancy analysis

To relate trait–trait covariation to trait–environment covariation, we performed a redundancy analysis (R package ‘vegan’). Ecoregion-aggregated traits (A2) were normalized and natural log-transformed. Scaled climate and soil variables were used as predictors. To decrease the factor that quantifies collinearity (variance inflation factor, vif), only the topsoil layer was selected (Fig. 4). For Supplementary Fig. 39, additional model tuning based on vif, with the exclusion of two variables with vif > 20, led to a model with vif < 10, which can be considered low cocorrelation.

### Reporting Summary

Further information on research design is available in the Nature Research Reporting Summary linked to this article.

## Supplementary information

Supplementary Information Supplementary Figs. 1–42 and Tables 1–8.

Reporting Summary

Peer Review Information

## Data Availability

Plant trait data were accessed from the TRY database (https://try-db.org, request no. 3282, date accessed July 2017, see also Extended Data Fig. 1). All TRY data required to reproduce this analysis, and the corresponding R scripts, are provided in an open TRY File Archive (https://www.try-db.org/TryWeb/Data.php). Climate data WorldClim are publicly available via https://www.worldclim.org/ (accessed May 2018). Soil data, namely SoilGrids (https://soilgrids.org/, accessed June 2018) are publicly available. Ecoregion information^30^ shapefiles are publicly available (accessed January 2014, Sciencebase.gov), The estimate of species richness per ecoregion^174^ is publicly available (accessed January 2014, databasin.org. Data for this study can be accessed on Github (https://github.com/juliajoswig/ Repo_ClimateSoil_TraitSpectrum). For Extended Data Fig. 1 and Supplementary Fig. 7, the Geodata product of the Missions Database ‘ArcWorld Supplement’ (GMI) was used.

## Code availability

The code is available on Github (https://github.com/juliajoswig/Repo_ClimateSoil_TraitSpectrum).
