# Supplementary material for: Climatic and soil factors explain the two-dimensional spectrum of global plant trait variation
Source: Nat Ecol Evol. 2021 Dec 23;6(1):36–50. doi: 10.1038/s41559-021-01616-8 (PMC8752441; doi:10.1038/s41559-021-01616-8)
Supplement: Supplementary file 1 — Supplementary Figs. 1–42 and Tables 1–8. [file 41559_2021_1616_MOESM1_ESM.pdf]

nature portfolio

https://doi.org/10.1038/s41559-021-01616-8

Supplementary information

Climatic and soil factors explain the two-dimensional spectrum of global plant trait variation

In the format provided by the
authors and unedited

nature portfolio

https://doi.org/10.1038/s41559-021-01616-8

Supplementary information

Climatic and soil factors explain the two-dimensional spectrum of global plant trait variation

In the format provided by the
authors and unedited

Supplementary material

Contents

1 Method robustness **4**

1.1 Independent effect of random noise 4

1.2 Bias test 5

1.3 Comparison to other Models 6

1.4 Selection criterion 6

2 Trait PCA **9**

3 Woody and non-woody subset **10**

4 Independent data **15**

5 Independent and joint effect of latitude and climate or soil **15**

6 Pattern robustness **17**

7 Pattern robustness **18**

7.1 Single trait-environment relationship patterns 18

7.2 Observed data 21

7.2.1 Trait clusters 21

7.2.2 Climate and soil: joint and independent effects 21

7.3 PCA models from ridge regression 22

8 Single trait analyses **23**

8.1 Redundancy analysis (RDA) with forward selection 37

9 The origin of the dip at the latitudinal gradient of PC2 **37**

9.1 Biomes at the Dip 38

9.2 Combined effect of climate and soil at the dip 39

10 Explained variance of size and economics traits depend on their coefficient of variation. 41

11 Data information 43

11.1 Climate data 43

11.2 Soil data 43

11.3 Trait data 43

11.4 Trait data for gap-filling 44

11.5 Ecoregion 44

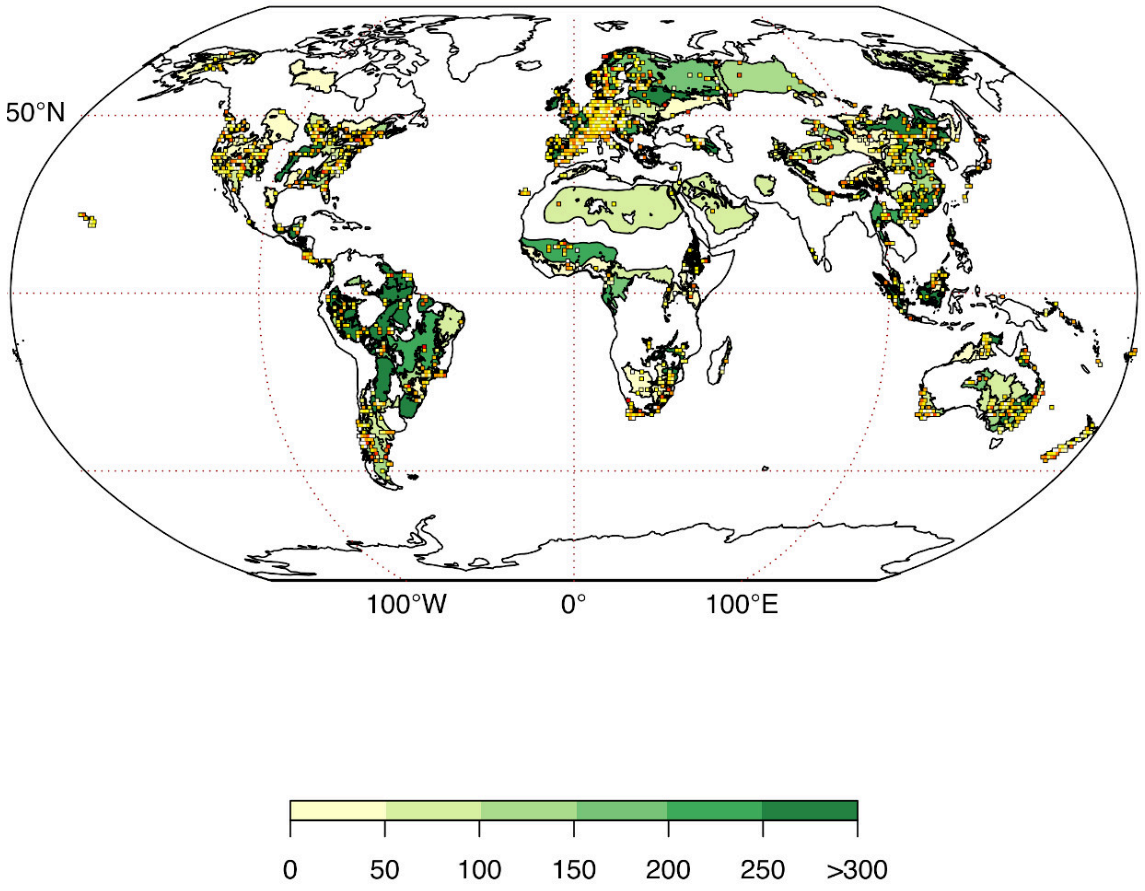

Figure 1 – Map of ecoregions[6] included in this study (n=220). The number of species per ecoregion is colour-coded (see Supplementary Tab. 7). Points indicate location of sampling aggregated to 1°; number of measurements are colour-coded from white=few, yellow= medium to red=many measurements. This map was created using the Geodata product of the Missions Database “ArcWorld Supplement” (GMI) available in the ArcGIS©software by Esri (and R). ArcGIS©and ArcMap^{TM} are the intellectual property of Esri and are used herein under license. Copyright ©Esri. All rights reserved. For more information about Esri®software, please visit www.esri.com.

1 Method robustness

The robustness of our model outputs was tested in four ways. First, we calculated how much variation can be explained by random noise, instead of real data on climate or soil variables (Supplementary Fig. 1). Second, we tested the method robustness for oversampled regions by reducing the number of species traits (Supplementary Fig. 2). Third, we tested the ridge regression results by comparison to results from other models: partial least squares (with and without PCA) and random forest (17). Fourth, we tested a different aggregation scale (grids of $1° * 1°$ in order to compare the signal-to-noise ratio with the ecoregion scale. Finally, we compared results of the analysis with and without our selection criteria (Supplementary Fig. 3), and of the ecoregion and grid scale aggregation. In all of these tests, the procedures we used in the main analysis performed as well as, or better than, the tested alternatives.

1.1 Independent effect of random noise

To estimate how much of the trait variation (r^2, ecoregion aggregation) is explained by random environmental variables (noise), we performed the ridge regression[7] + hierarchical partitioning[2] as described above (methods „ridge regression“ and “hierarchical partitioning”) and paired soil or climate with noise. The noise data set comprised randomly sampled values for a variable set as large as the soil variable set (n=107). We performed ridge regression[7] analysis with noise data, together with soil or climate. Then we calculated the independent effect of noise from soil or climate data. The independent effect of randomized data (noise) is always 0 or even negative, due to large differences between r^2_total and r^2_noise, and model variability. Overall, noise never has an independent effect greater than zero, and the joint effect can be as large as 9%.

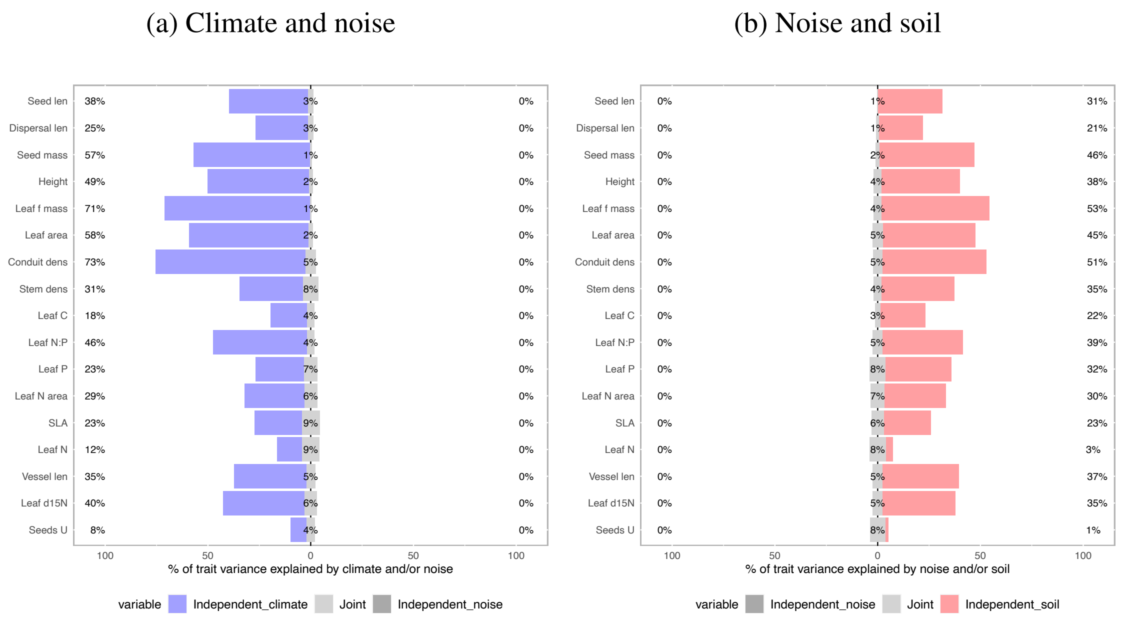

Figure 1 – Proportion variance in each trait explained by noise, climate or soil variables (ecoregion median trait, blue=size, red=economics, yellow=other). (Left) Variance of each trait explained by noise and climate variables (sorted according to trait groups: size, economics, other). Total bar length=total r^2 explained by climate and noise. Purple fraction=explained by the independent climate effect, gray bars=fraction that is explained by climate and noise joint effect, and thus symmetric. Trait with the highest joint effect are Leaf N and SLA for which the joint effect explains 9%. (Right) Variance of each trait explained by noise and soil (sorted according to traitgroups: size, economics, other). Total bar length=total r^2 explained by noise and soil. Purple fraction=explained by the independent soil effect, gray bars=fraction that is explained by soil and noise joint effect, and thus symmetric, purple bars=fraction that is explained by noise alone (max=1%). Trait with the highest joint effect is Leaf P for which the joint effect explains 8%. Values represent the mean of 50 model runs, 220 ecoregions.

1.2 Bias test

We reduced the number of species traits in order to account for oversampling in certain ecoregions. Our approach was to reduce the number of species in those ecoregions which exceeded our selection criteria (>20 species and >1% of species richness accoding to Kier[5]). This allowed us to keep all 220 ecoregions, while changing the data distribution among species. Species trait values were deleted randomly, and only then aggregated to ecoregions (repetitions n=3; termed as bias1, bias2, bias3). This data was then analysed as described in the methods by ridge regression (n=50) and hierarchical partitioning.

(a) Bias test run 1 (b) Bias test run 2 (c) Bias test run 3

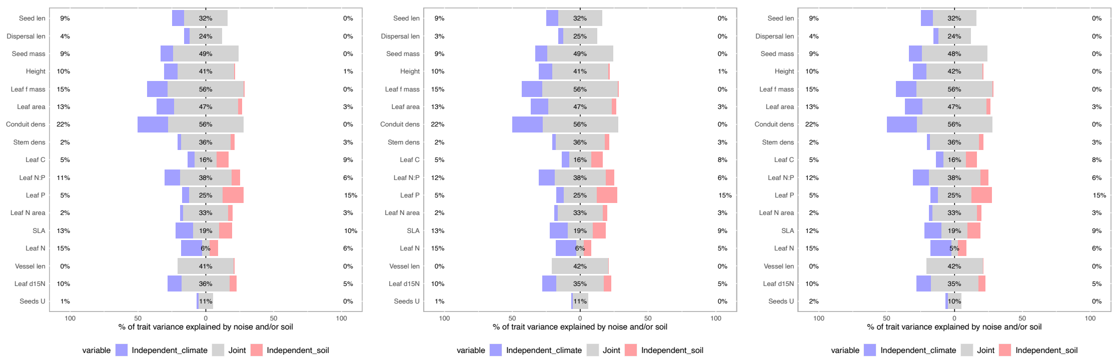

Figure 2 – Bias test results run with the minimum number of species information (trait values per ecoregion above minimum selection criterion). Trait values of species above the selection criterion were randomly deleted. Everything else remained as explained in the methods or as in Figure 3b.

1.3 Comparison to other Models

We compared the model outputs of alternative models of partial least squares and random forest to those of the ridge regression[7]. We modelled all 17 plant traits from soil variables only, climate variables only and soil with climate variables. We thus ran a 10-fold cross-validated partial least squares model (PLS) with 10 repetitions, in addition to a 10-fold cross-validated random forest with 2 repetitions. Afterwards we subjected the r^2 of soil only, climate only and soil with climate to hierarchical partitioning[2]. We find similar explained variances (17).

1.4 Selection criterion

(a) (b)

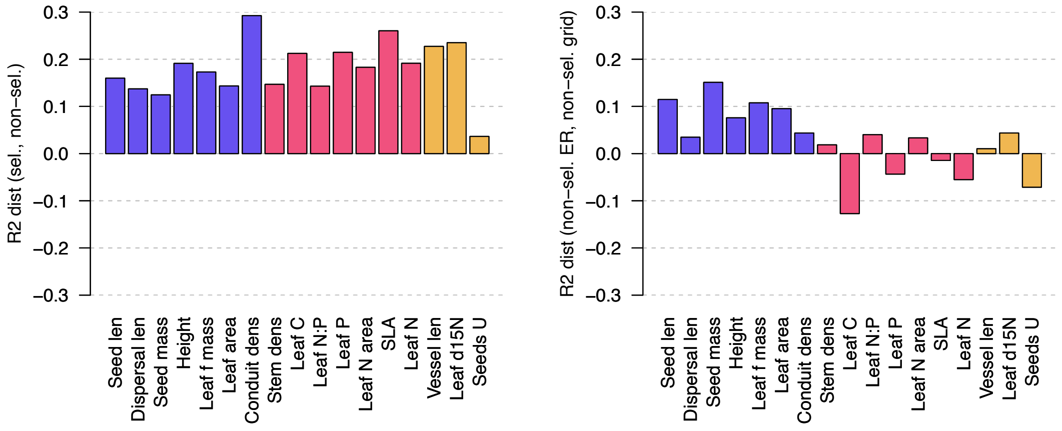

Figure 3 – Comparison of explained variance by ridge regression models from climate and soil variables, with focus on the level of aggregation and selection criteria. Due to the missing information of an estimate of species richness (equivalent of Kier[5] species richness) for grid level, all grids entered the analysis, independent of the number of observations. Ecoregions were selected based on the selection criterion as in the main analysis (details see methods). (a): Distance between the data on ecoregions with and without selection criterion ($n_{no-selection}$=422, $n_{selected}$=220), positive values mean a higher explained variance for models with the selection criterion. (b): Distance between data on ecoregions versus grid scale (without selection criterion, n=1,542). Bars colored according to size (blue), economics (red) and other traits (yellow).

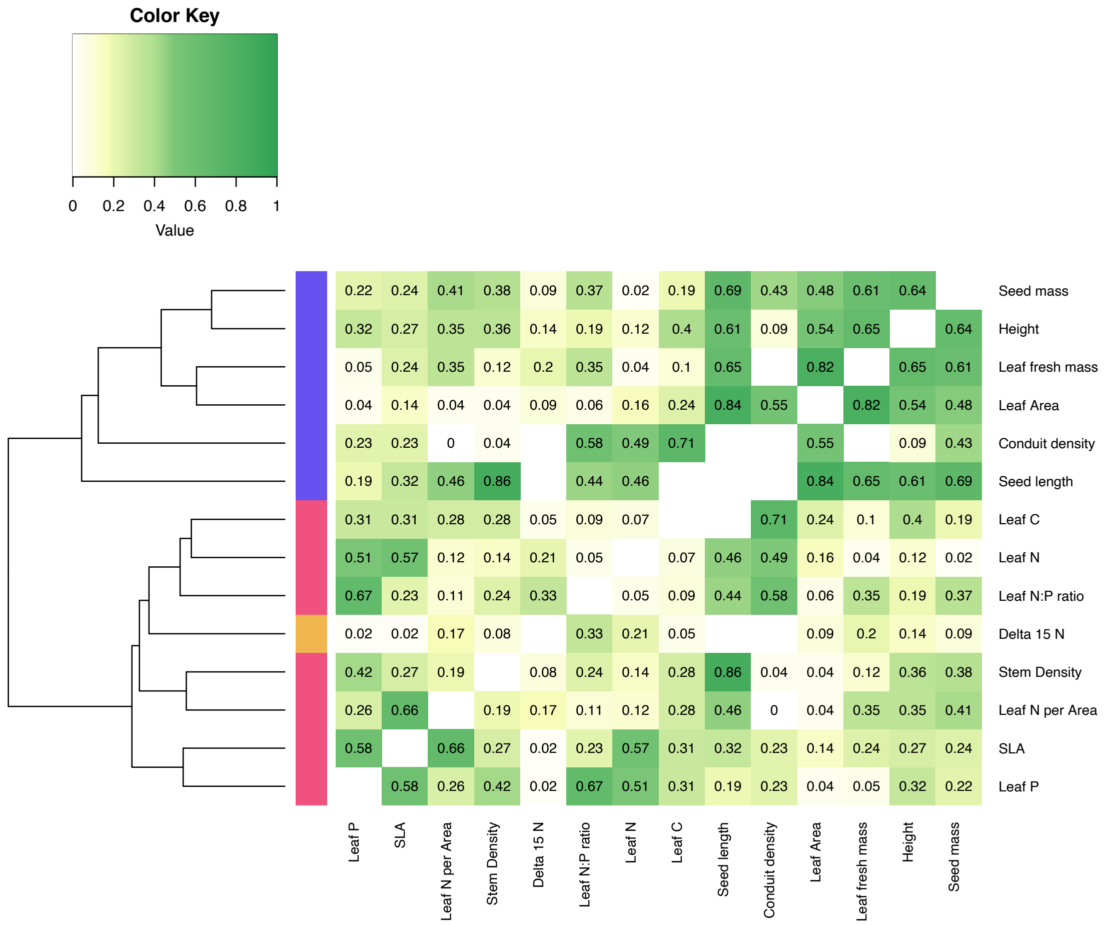

Figure 4 – Covariance of plant functional traits from observed trait values only ($n_{Seed.mass}$=89, n_{Height}=277, $n_{Leaf.fresh.mass}$=, $n_{Leafarea}$= 244, $n_{Seed.length}$=, n_{LeafC}=74, n_{LeafN}=262, $n_{Leaf.N:P.ratio}$=147, $n_{Delta.15N}$=88, $n_{Stem.Density}$=88, $n_{Leaf.N.per.Area}$=156, n_{SLA}=212, n_{LeafP}=201; species per ecoregion aggregation; see Methods). Traits were analysed by cluster analysis (hierarchical clustering) based on absolute pairwise Pearson correlation coefficient. Green shades indicate high absolute correlation and yellow shades indicate low absolute correlation. On the left, the distance tree of traits derived from hierarchical clustering is illustrated. Two resulting groups are: 1. size-related traits (blue) consisting of conduit density, leaf area, leaf fresh mass, height, seed mass; 2. a mixture of economics (red) and one other trait (yellow) comprising specific leaf area (SLA), leaf N content per area, leaf N, P and C concentrations, leaf N/P ratio, stem specific density (stem dens) and Delta 15N.

2 Trait PCA

We computed PCAs based on traits of single species per ecoregion (n=36,197). The variance explained by each component is shown in (Supplementary Fig. 5). The first two axes each explain more than 10% of variation (left, PC3=9.36%) and together almost 50% of the overall variation of the 17 plant traits (right). The trait loadings onto the first 5 principle components (PCs) are presented in Supplementary Fig. 6. Size traits load most onto PC1, economics traits onto PC2 and PC3. Overall the loadings decrease with increasing PCs.

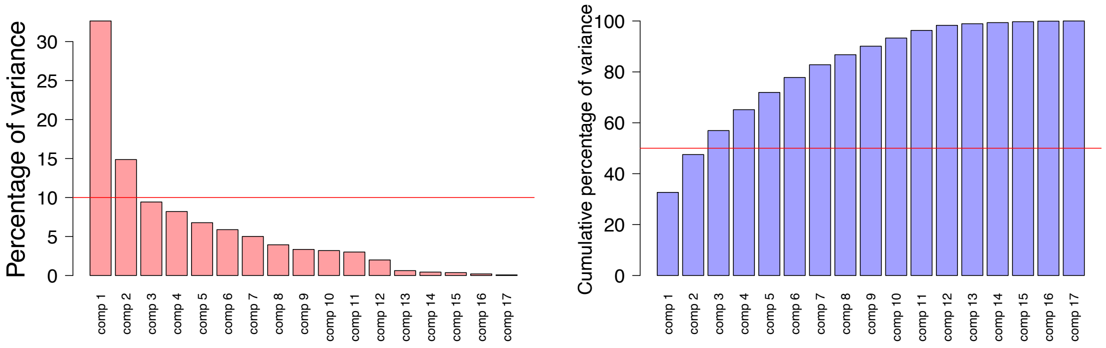

Figure 5 – Variance of the trait PCA axes (17 traits, n=36,197 single ecoregion species). (Left) Red lines refer to 10% of variance, and (right) 50% of variance.

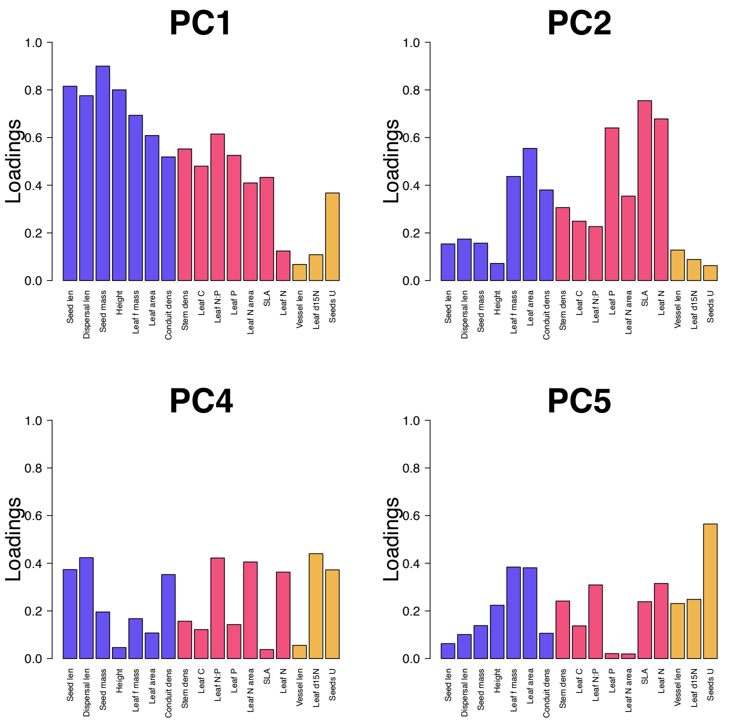

Figure 6 – Absolute loadings of the first 5 trait PCA axes (17 traits, n=36,197 single ecoregion species), trait bars colored according to trait group (blue=size, red=economics, yellow=other)

3 Woody and non-woody subset

Diaz and colleagues (2016)[3] depict the global distribution of 6 traits to include two data-rich hotspots of species with similar trait correlations: one set of woody and one set of non-woody species, and thus we look in closer detail at these two groups. The distributions of resulting ecoregions are shown in Supplementary Fig. 7. As we retain our quality control criteria of at least 20 species per ecoregion representing at least 1% of estimated species richness[5], dividing our analysis into these two plant groups results in a decreased total number of species (n_{woody}=14,534, $n_{nonwoody}$=13,042) and ecoregions (n_{woody}=86, $n_{nonwoody}$=84) in comparison to our main analysis ($n_{species}$=36,197, ecoregions $n_{ecoregions}$=220). Which ecoregions are selected also differs between non-woody (7a) and woody species (7b) The trait-trait relationships are shown in 8a, 8b in the form of correlations and as PCAs (8c, 8d). Also in these subgroups (woody and non-woody),

we recover similar trait clustering, mostly into groups of size and economics traits (Supplementary Fig. 8); for woody traits, there are additional clusters of hydraulic and seed traits. The size traits again load primarily onto the first axis, and economics traits primarily onto the second axis of the PCA. Latitudinal gradients of these PCs are shown in Supplementary Fig. 9. The ridge regression plus hierarchical partitioning are shown in Supplementary Fig. 9. Woody species traits appear to be more influenced by climate in comparison to non-woody species traits, which appear to be influenced more by soil variables. All analyses were carried out exactly as in the original study. Please note all negative values from hierarchical partitioning[2] were removed by replacement with zeros. Negative values can result from model instabilities that shift average $r^2_{climate\_and\_soil}$ to being smaller than $r^2_{climate}$ or r^2_{soil}.

(a) Non-woody species (b) Woody species

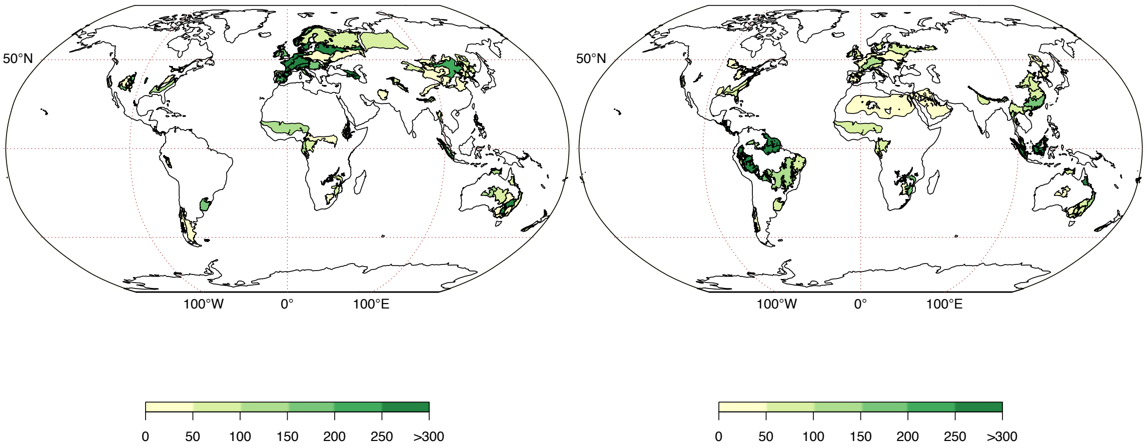

Figure 7 – Results of subsetting the total plant data into a woody and a non-woody subset; (a) non-woody and and (b) woody species: Geographic distribution of selected ecoregions[6] (n_{woody}=86 and $n_{non-woody}$ =84, from selection criterion (>1% of estimated Kier[5] species richness and >20 species) with number of species for woody and non-woody subsets. These maps were created using the Geodata product of the Missions Database "ArcWorld Supplement" (GMI) available in the ArcGIS©software by Esri (and R). ArcGIS©and ArcMap^{TM} are the intellectual property of Esri and are used herein under license. Copyright ©Esri. All rights reserved. For more information about Esri®software, please visit www.esri.com.

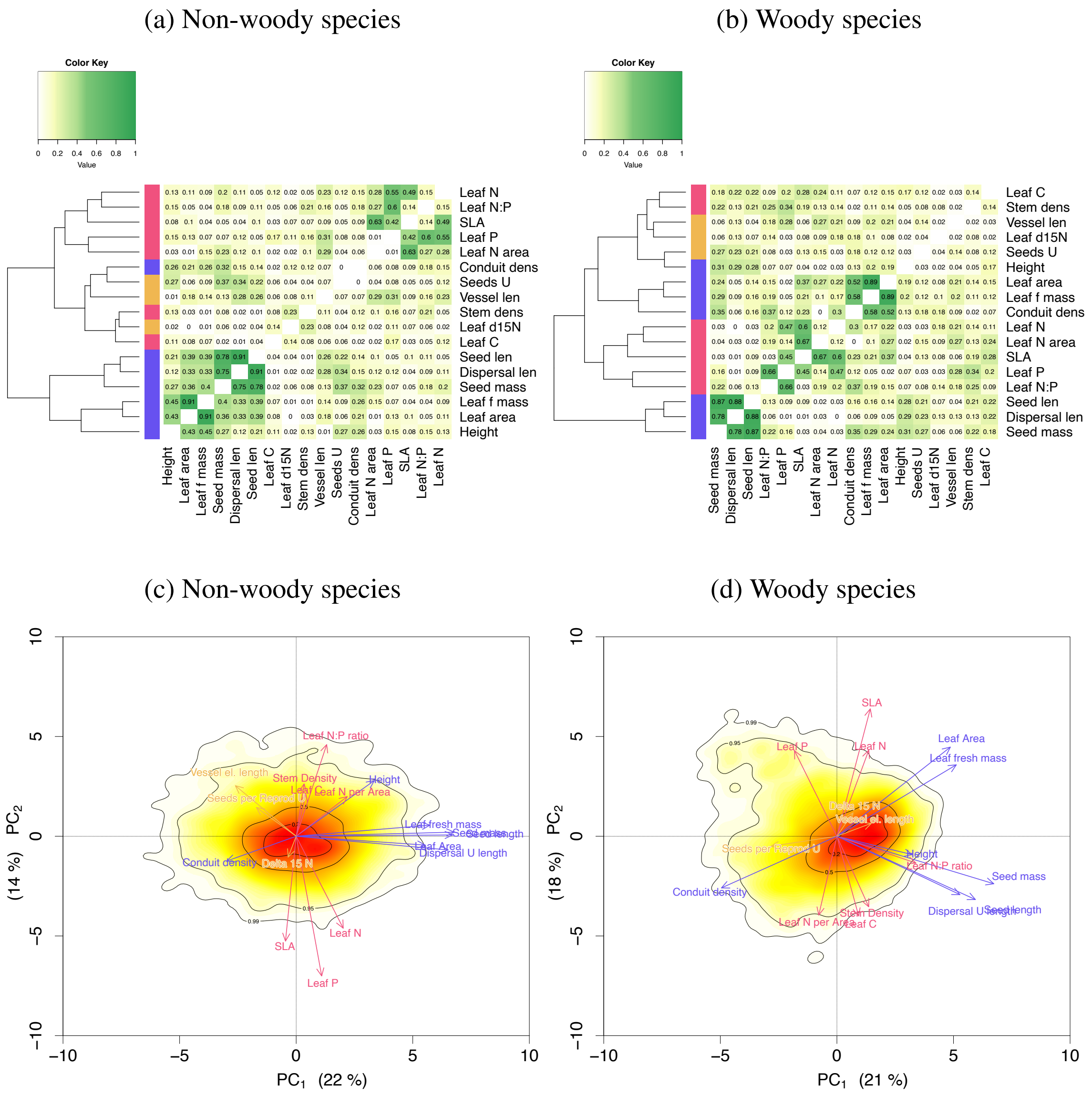

Figure 8 – Trait groups for woody and non-woody plants derived from covariance of plant functional traits (a, non-woody and and b, woody species; species median aggregation; n_{woody}=14,534. $n_{non-woody}$=13,042; gap-filled, see Methods) from Pearson correlations coefficients (absolute) with trait groups from hierarchical clustering and (c+d) Principal Components Analysis (PCA). Trait row box colored according to the trait group (blue=size, red=economics, yellow=other).

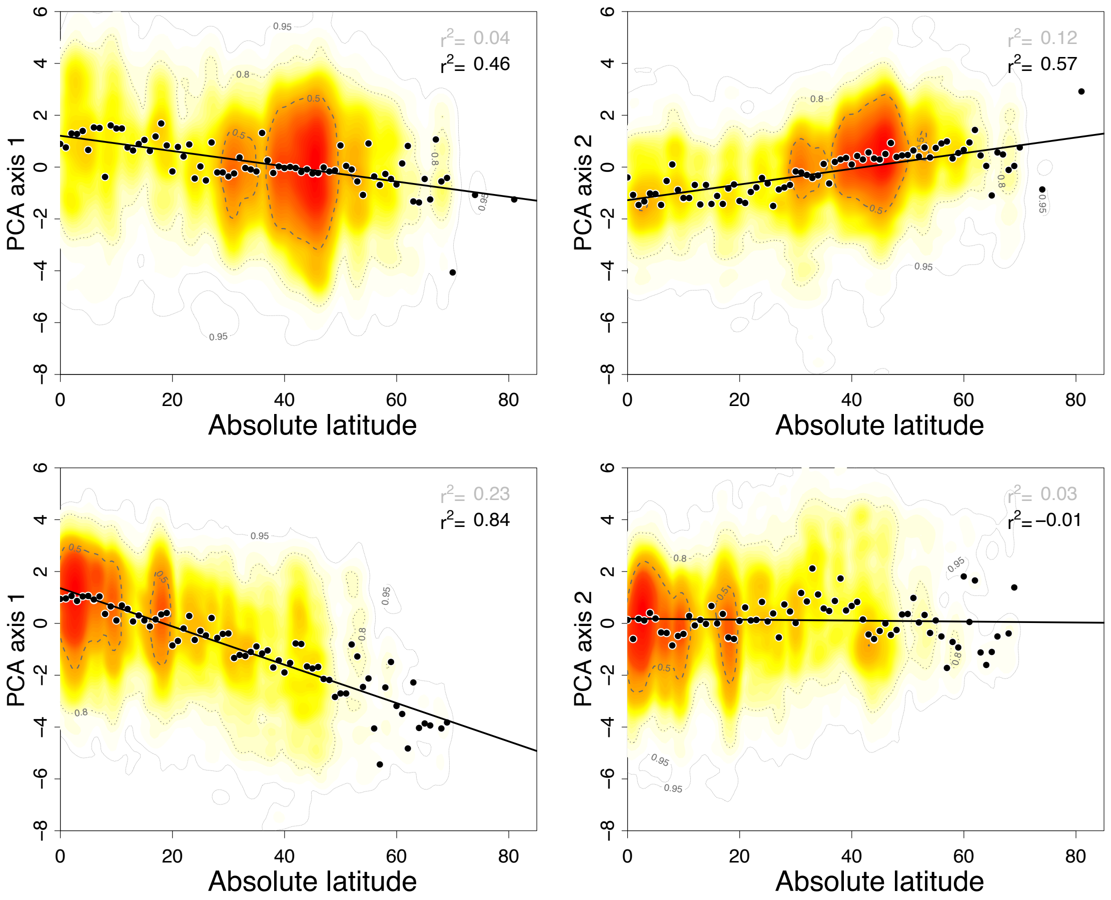

Figure 9 – Latitudinal gradients of the first two principal components of the PCA on the 17 ecoregion species median plant traits; woody (top, n_{woody}=14,534) and non-woody species (bottom, $n_{non-woody}$=13,042). The analysis of woody species is shown at the top, and of non-woody species at the bottom; on the left, PC1 (most influenced by size traits) and on the right, PC2 (most influenced by economics traits) are regressed against absolute latitude. Colors are according to density of species (unique species per ecoregion[269]). Mean estimates aggregated at 1° absolute latitude are indicated as black dots. Lines refer to the linear models for PC1 (explaining 21% for woody and 22% for non-woody of trait variation) scores binned to absolute latitudes against latitude (r^2_{woody}=84% or $r^2_{non-woody}$=46%; compared to $r^2_{non-woody}$=23% or $r^2_{non-woody}$=4% without bins); and PC2 (explains trait variation by 18% for woody and 14% for non-woody) scores binned to absolute latitudes against latitude (r^2_{woody}=3% or $r^2_{non-woody}$=57%; compared to r^2_{woody}=0% or $r^2_{non-woody}$=12% without bins). The density of points indicates that the species richness[5] hotspot in our data for woody plants is situated closer to the equator (top), for non-woody plants more in the temperate regions (bottom), and this reproduces the data basis (Figure 3a and Figure 3b) and the general finding of a shift from tree to herbaceous species richness from low to high latitudes Moles2009. The second PC, representing mainly economics traits, shows strong non-linear effects. This can be seen for both subsets with species-level aggregation, yet only for woody plants with latitudinal bins(b,d).

4 Independent data

Because our dataset lacked data points from high latitudes, we next determined whether a publicly available, independent data set from the tundra would support different relationship of size and economics traits with latitudinal gradients.

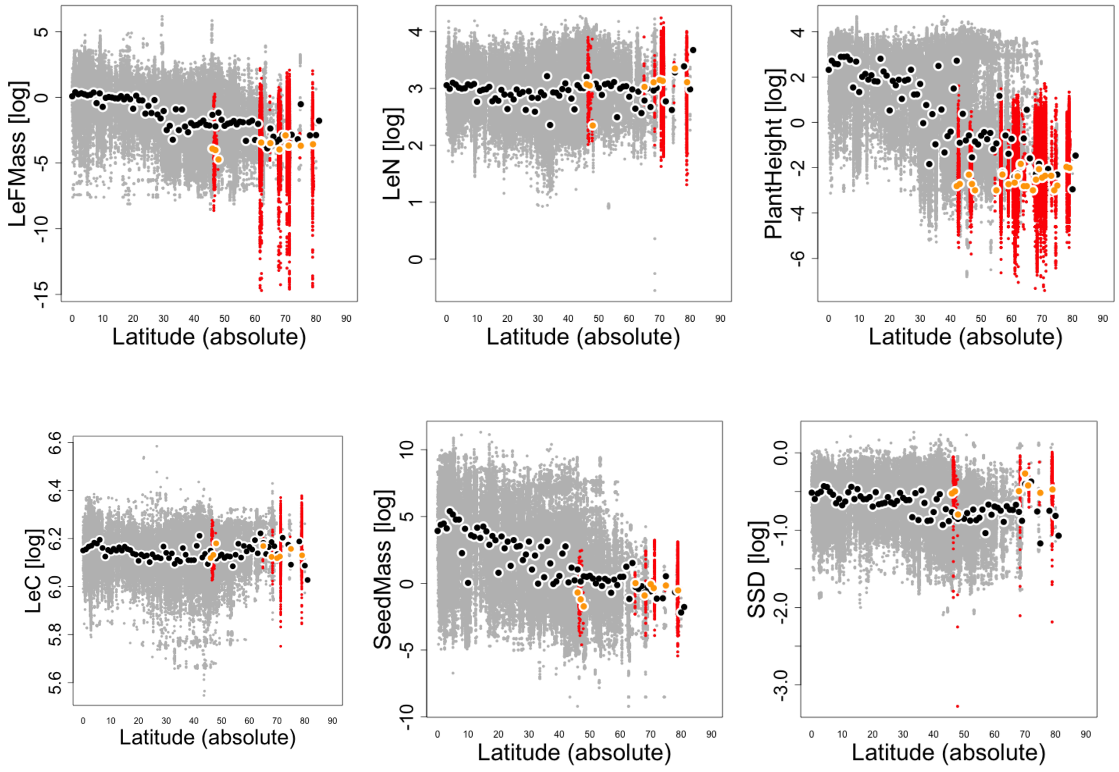

Figure 10 – Latitudinal trait gradients of data used in this study (gray), wth the addition of data from the tundra trait team[1] (red). Points circled in white (black, orange) refer to binned trait values (absolute latitudinal degree median) of the data used in this study and the tundra data, respectively.

5 Independent and joint effect of latitude and climate or soil

The analysis was conducted in the same way as the original analysis (see Methods), only replacing one of the variable types (climate or soil) with latitude. Latitude was represented

by the median, maximum and minimum of the ecoregion. The comparison of latitude and ecoregions demanded an aggregation to ecoregions, also for latitude.

(a) (b)

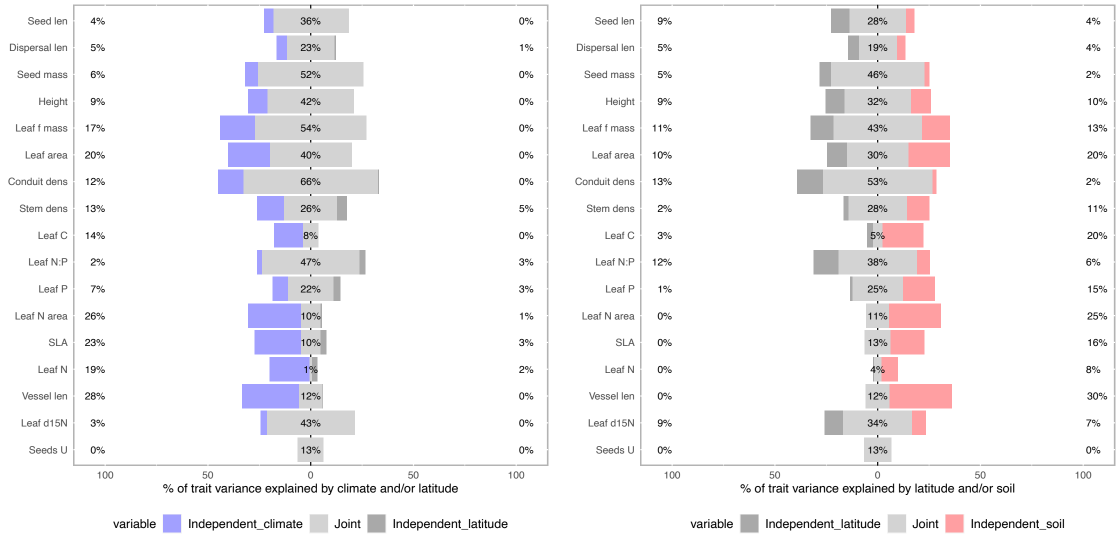

Figure 11 – Latitude (gray) and soil (peach) or climate (purple) explain traits. (a) Ridge regression (RR) and hierarchical partitioning result of climate versus latitude. (a) RR and hierarchical partitioning result of latitude versus soil. Traits are ordered according to trait groups (economics, size, other).

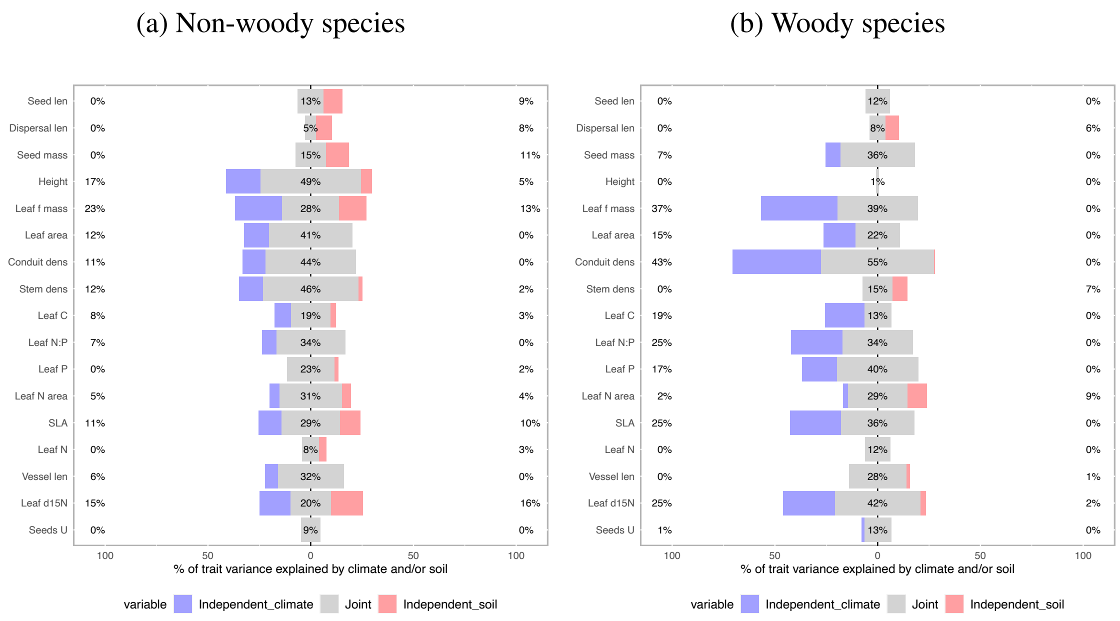

Figure 12 – Trait variation explained by climate and soil variables for (a) non-woody and and (b) woody species (ecoregion[6] median trait; sorted according to trait group, repetitions n=50). Total bar=total r^2 explained by climate (purple) and soil (peach). Red fraction=explained by the independent soil effect, blue fraction=explained by the independent climate effect. Gray bars=fraction that is explained by climate and soil joint effect. Variance explained for total, woody and non-woody subset by climate and soil. Bars are split into the independent and joint (gray) effect by climate (blue) and soil (red) to explain each trait. Traits are ordered according to trait groups (economics, size, other).

6 Pattern robustness

We tested if the patterns (large joint effect, climate relevant for all traits, soil with additional information for economics traits) are reflected in higher or lower levels. We had three approaches. First, we analysed single variable-trait relationships by using explained variance from linear models (r^2) and their resulting pattern by hierarchical clustering (Supplementary Fig. 13). Second, we conducted a meta-analysis for the first 5 principle components (14), which were explained in a pattern similar to that for the traits which load on these axes. Third, we reduced the number of variables going into the RDA by forward selection, which again reveals unique aspects of climate and soil for size or economics traits (Supplementary Fig. 39).

7 Pattern robustness

We tested if the patterns (large joint effect, climate relevant for all traits, soil with additional information for economics traits) are reflected in higher or lower levels. We had three approaches. First, we analysed single variable-trait relationships by using explained variance from linear models (r^2) and their resulting pattern by hierarchical clustering (Supplementary Fig. 13). Second, we conducted a meta-analysis for the first 5 principle components (14), which were explained in a pattern similar to that for the traits which load on these axes. Third, we reduced the number of variables going into the RDA by forward selection, which again reveals unique aspects of climate and soil for size or economics traits (Supplementary Fig. 39).

7.1 Single trait-environment relationship patterns

In this analysis we tested the pattern of explained variance of traits from linear models using one environmental variable only. Variance explained (r^2) from linear models were calculated with 10-fold cross validation (only unseen trait values were predicted) with 10 repetitions that were averaged (mean) for this figure. Hierarchical clustering shows similar groups of traits, based on their r^2 pattern.

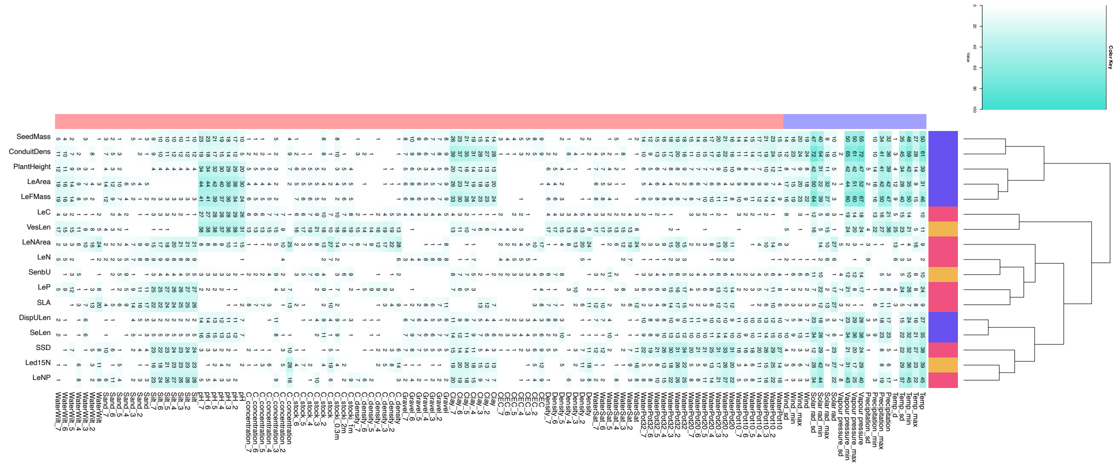

Figure 13 – Overview of trait-environment relationships plus the relative similarity of traits. This figure is based on the explained variance of traits from linear models with one environmental variable only (ecoregion aggregation). The top bar color indicates variable type: blue=climate, red=soil. Colors within the plot show an increase in explained variance (r^2) from white to turquoise; empty squares indicate zero values for the purpose of better visualization (zero values entered the analysis). The right bar color indicates the trait group (blue=size, red=economics, yellow=other). The dendrogram shows the hierarchical clustering result.

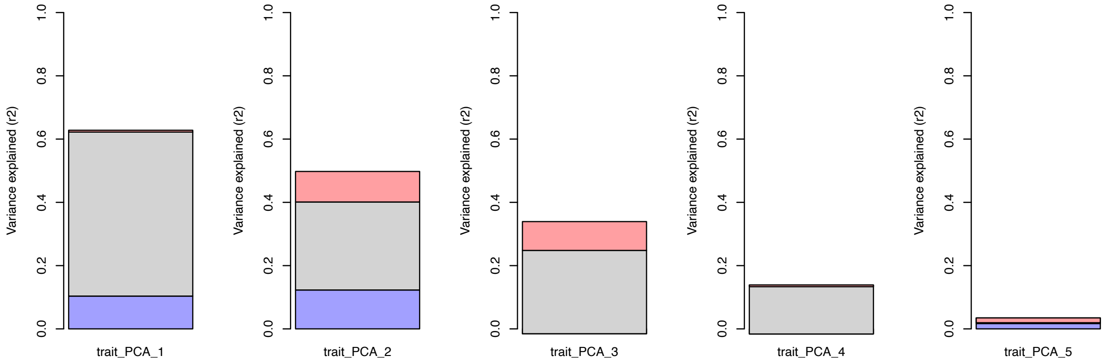

Figure 14 – Variance explained by ridge regression for first and second PC (ecoregion[6] aggregation), with independent effect of climate (blue) and soil (red) and their joint effect (gray). Ridge regression repetitions n=50, averaged.

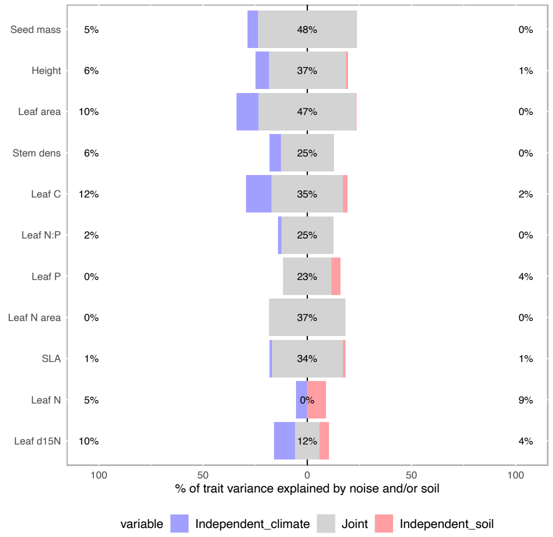

Figure 15 – Ridge regression and hierarchical partitioning results with observed data only. All traits with more than 50 ecoregions were included. No selection criteria to select for ecoregions (repetitions n=50, $n_{Seed.mass}$=89, n_{Height}=277, $n_{Leaf.fresh.mass}$=, $n_{Leafarea}$= 244, $n_{Seed.length}$=, n_{LeafC}=74, n_{LeafN}=262, $n_{Leaf.N:P.ratio}$=147, $n_{Delta.15N}$=88, $n_{Stem.Density}$=88, $n_{Leaf.N.per.Area}$=156, n_{SLA}=212, n_{LeafP}=201). Colored according to size (blue), economics (red) and other traits (yellow).

7.2 Observed data

We next wanted to learn if the pattern with observed (not gap-filled) data is similar to the pattern observed from gap-filled data. We used two approaches: First, we clustered the observed traits into trait groups (Supplementary Fig. 4), second, we retrieved simple trait variance explained from single environmental variables and show results in a heatmap F(igure Supplementary Fig. 16). Third, we ran the analysis as for Figure 3.

7.2.1 Trait clusters

Instead of using the complete 615,349 trait values spread over 17 traits (species aggregation), we performed this analysis with 96,055 trait values, 6.4 times less than the gap-filled version. The traits cluster into very similar trait groups (Supplementary Fig. 4). The correlation coefficients are higher in the gap-filled data, but the pattern remains the same. With observed data only, the analysis for Figure 3 (ridge regression in combination with hierarchical partitioning) is hard to reproduce due to data shortage. Yet for the traits available, Supplementary Fig. 15 tends to reproduce the pattern of joint and independent effects. All data were included in this analysis. Only those traits were admitted to the analysis with more than 50 ecoregion values. No selection criterion was applied

7.2.2 Climate and soil: joint and independent effects

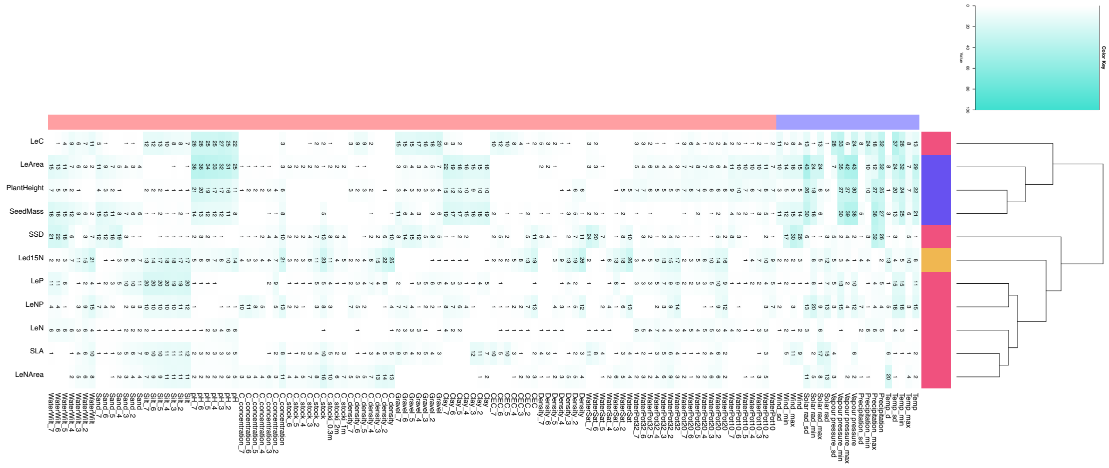

Figure 16 – Overview of trait-environment relationships plus the relative similarity of traits, from observed trait values only. This figure is based on the explained variance of observed traits from linear models with one environmental variable only (ecoregion aggregation, only traits with >50 ecoregions were included, no selection criterion for ecoregions). The top bar color indicates variable type: blue=climate, red=soil. Colors within the plot show an increase in explained variance (r^2) from white to turquoise; empty squares indicate zero values. The right bar color indicates the trait group (blue=size, red=economics, yellow=other). The dendrogram shows the hierarchical clustering result.

7.3 PCA models from ridge regression

Instead of traits, we used the first 5 principle components as response variables. The analysis was performed as described in the Methods section. In sum we find that PC1 is best explained, and most by climate, as is the case for size traits; PC2 is explained by both climate and soil. The third axis is mainly explained by soil variables only. These results are consistent with our finding of a strong joint effect, and of size traits loading strongly on the first principle component being better explained by climate, while economics traits that load mainly on the second principle component are explained by both climate and soil independently in addition to their strong joint effect.

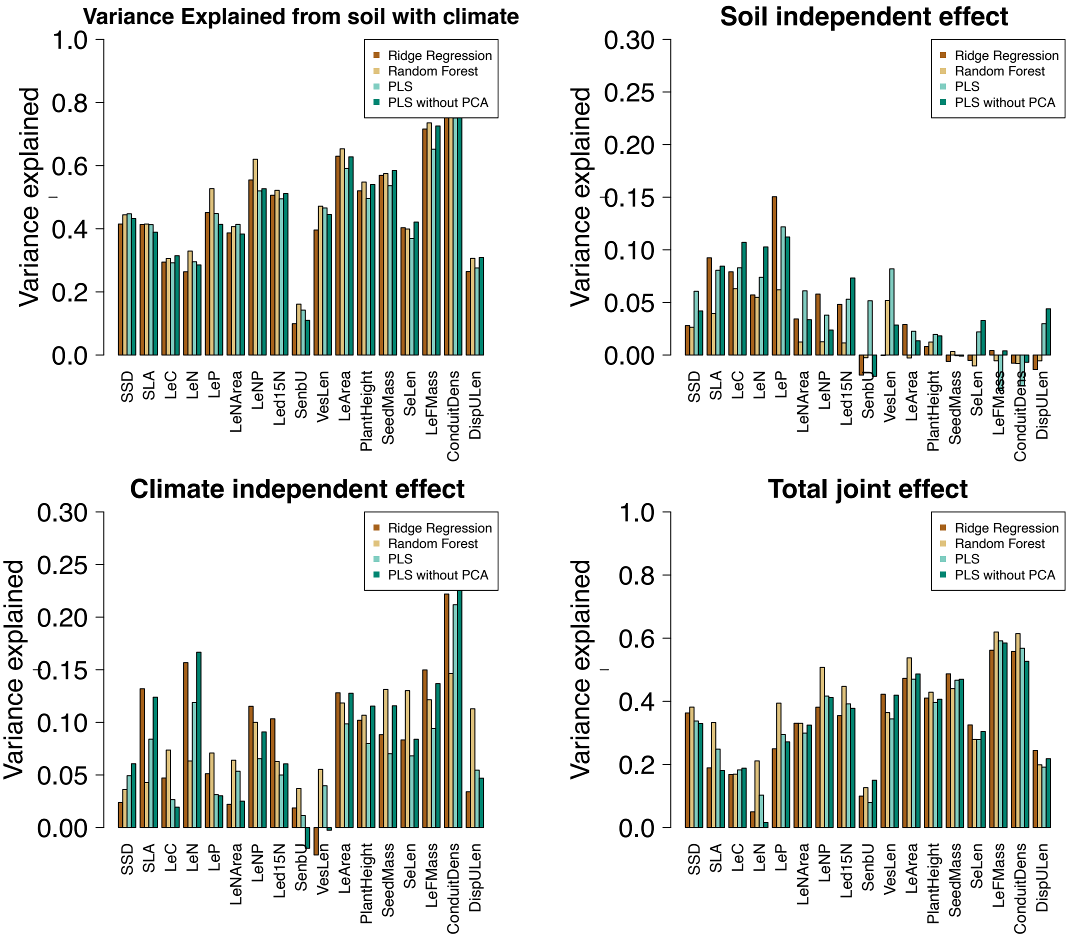

Figure 17 – Comparison of results from different models to explain the 17 plant traits, namely ridge regression[7], random forest, PLS and PLS without prior dimensionality reduction of soil and climate variables to 20 PCA axes (PLS without PCA): (top) Variance explained per trait (r^2). (second) Soil independet effect (excluding any joint effect), calculated from hierarchical partitioning[2], to explain the 17 traits. (third) Climate independent effect (excluding any joint effect), calculated from hierarchical partitioning[2], to explain the 17 traits. (bottom) Total joint effect (Fraction explained by both soil and climate variables) to explain the 17 traits. Please note the different y-axes.

8 Single trait analyses

We investigated relationships to soil and climate variables for individual traits. We present in Supplementary Figs. 19-35 an individual characterization of each trait in terms of covariance with other traits, latitudinal gradient, and climate and soil contributions to explain trait variation.

We provide an overview of trait-environment relationships plus the relative similarity

of traits (Template: Supplementary Fig. 18). We show in (a) the correlation pattern colored to absolute strength (Pearson correlation coefficient). This traits' latitudinal gradient (species per ecoregion[6] median aggregation n=36,197) is shown in panel(b). Mean estimates aggregated at 1° absolute latitude are indicated as thick dots. The barplots in graph (c) include r^2 of three models (climate and soil, climate only soil only) additionally to the climate and soil subgroups contributions to make up r^2 (for subgroup attribution see Supplementary Tab. 3). These barplots are the result of hierarchical partitioning[2], i.e., the independent and joint effects add up to the r^2 in the respective model. For instance if 50% of a trait is explained by climate and soil variables (left barplot), then the sum of independent (climate or soil) and joint (climate and soil) effects equals 50%. Figure (d) displays a riverplot including the relative contribution of climate and soil with variable subsets to explain a trait. The sum of independent effects on the lowest level (subset of climate and soil variables) were scaled to independent effects on respective higher level (climate or soil). The joint effect is omitted so that the plot only shows the independent effect without any joint share. Therefore only variable sets are shown that add information in comparison to the counterpart. The independent effect is then scaled to the higher-level independent effect. E.g. if independent effect_1=0, independent effect_2=0.2 joint $\text{effect}_{1\&2}$=.1, then variable 1 is not shown. With variable set 1 and 2 belonging to soil, then the independent effect_2 =0.2 is for this case equal to the independent effect of soil. Figure (e) displays the variance explained of simple linear models (ecoregion aggregation, 10 fold cross validation average of 50 repetitions as in Supplementary Fig. 13). Colors refer to the Pearson correlation coefficients of the same data (blue= negative, white=low, red=positive). For abbreviations of climate and soil variables see Supplementary Tab. 1 and Supplementary Tab. 2.

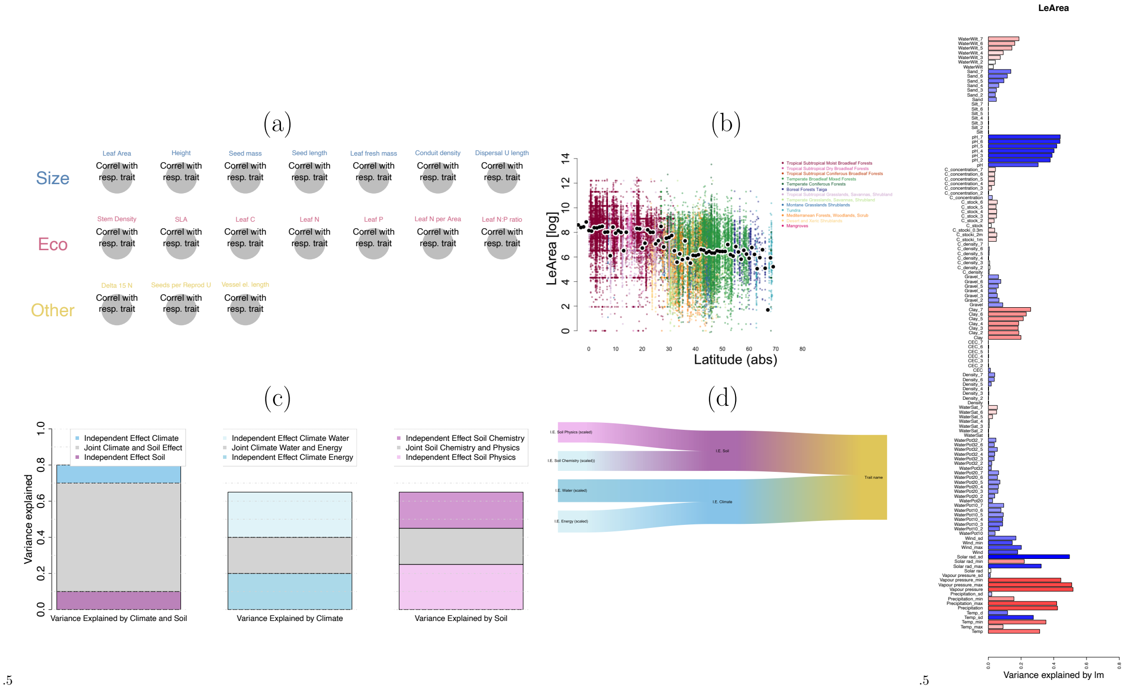

Figure 18 – Template explaining the way to read the single trait figures.

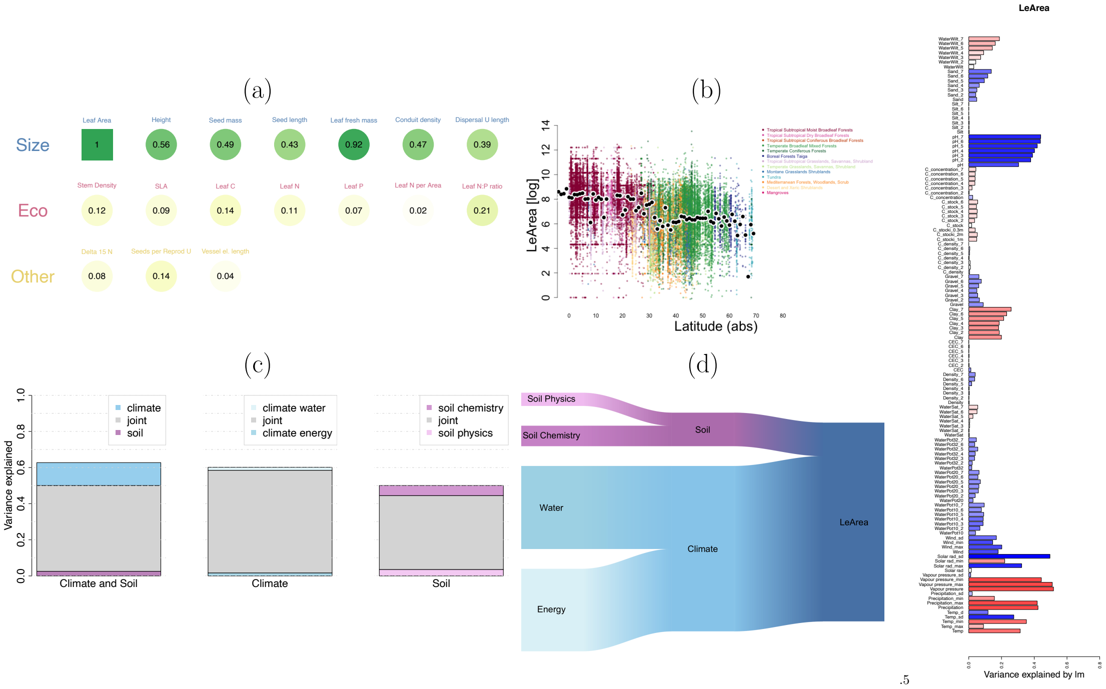

.5 .5

Figure 19 – Leaf area, size trait.

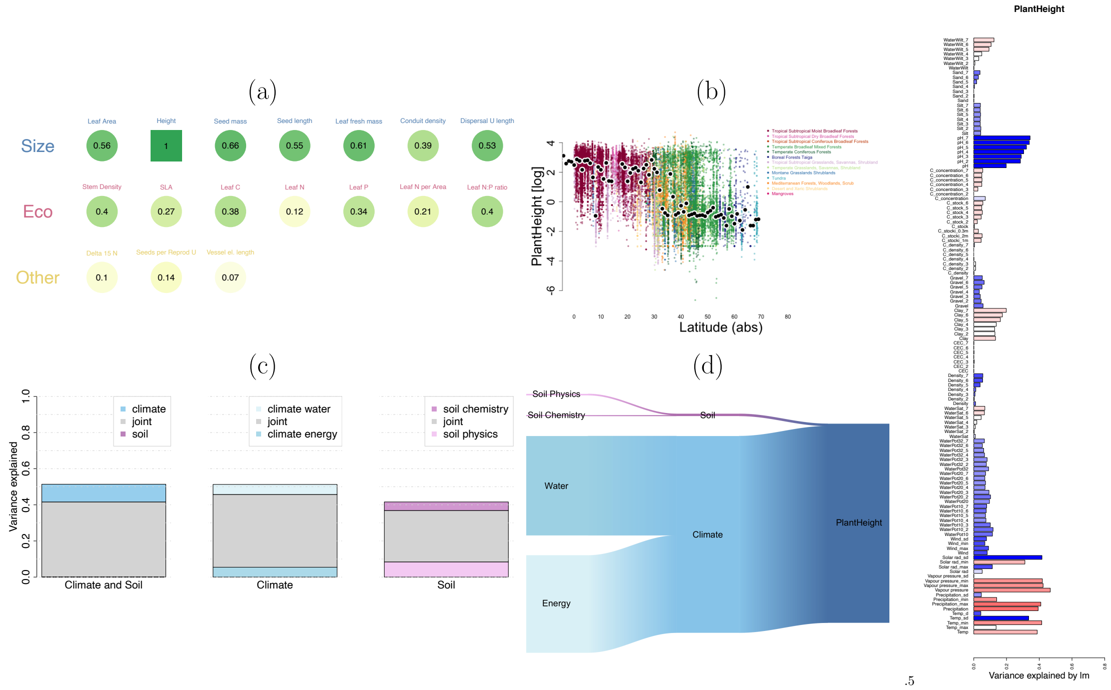

.5 .5

Figure 20 – Plant height, size trait.

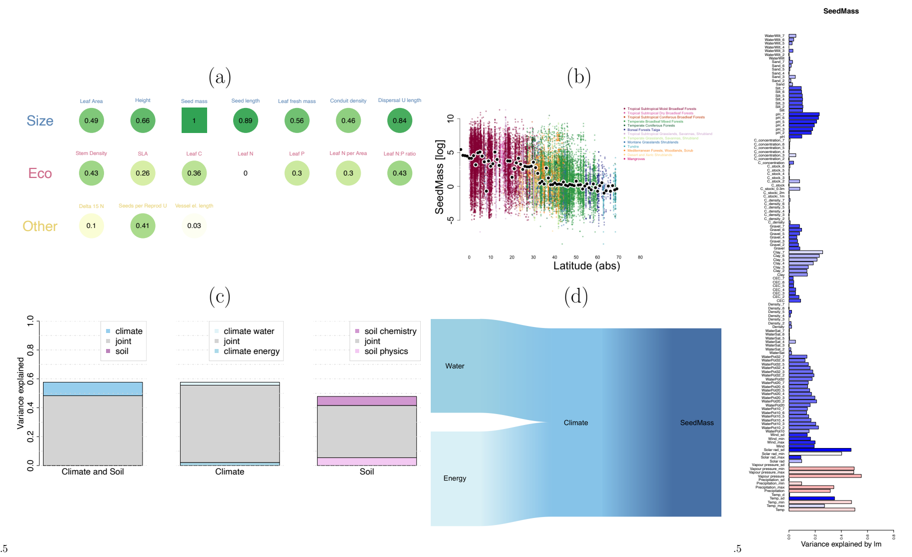

.5 .5

Figure 21 – Seed mass, size trait.

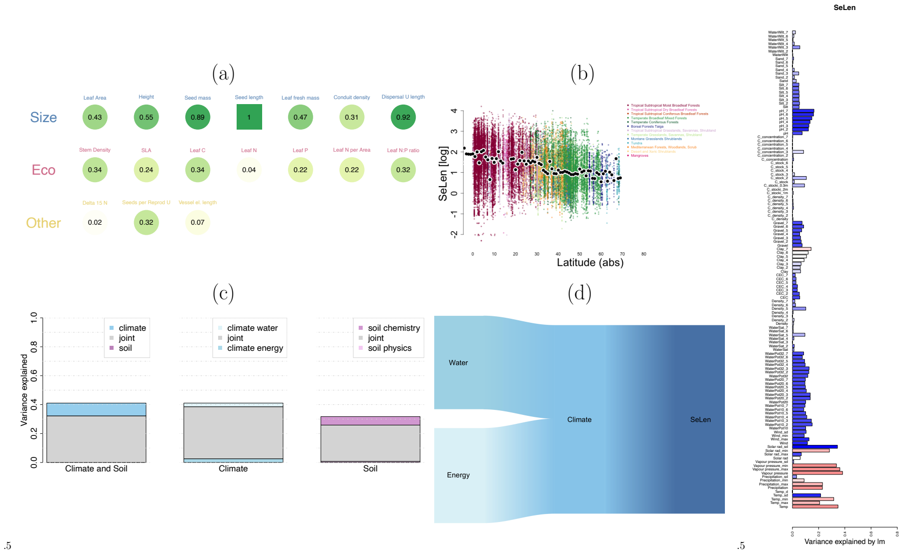

Figure 22 – Seed length, size trait.

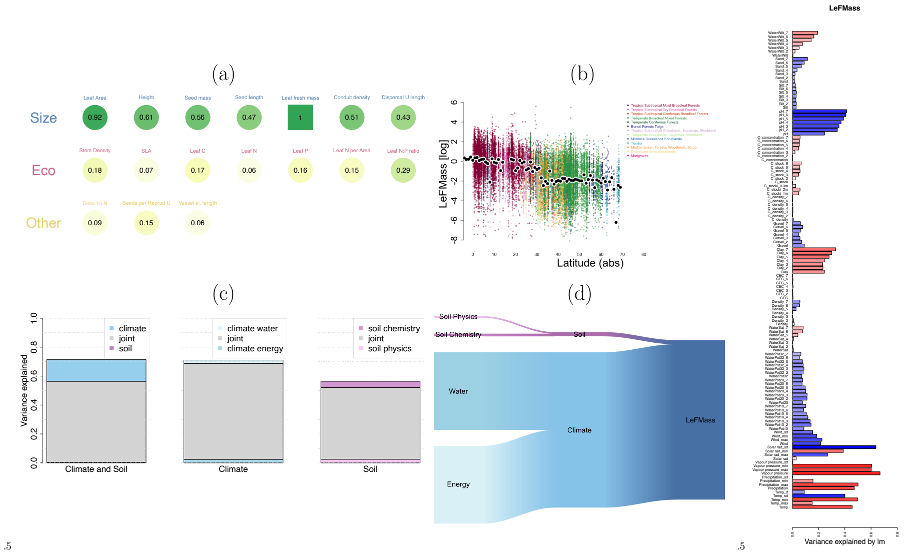

Figure 23 – Leaf fresh mass, size trait.

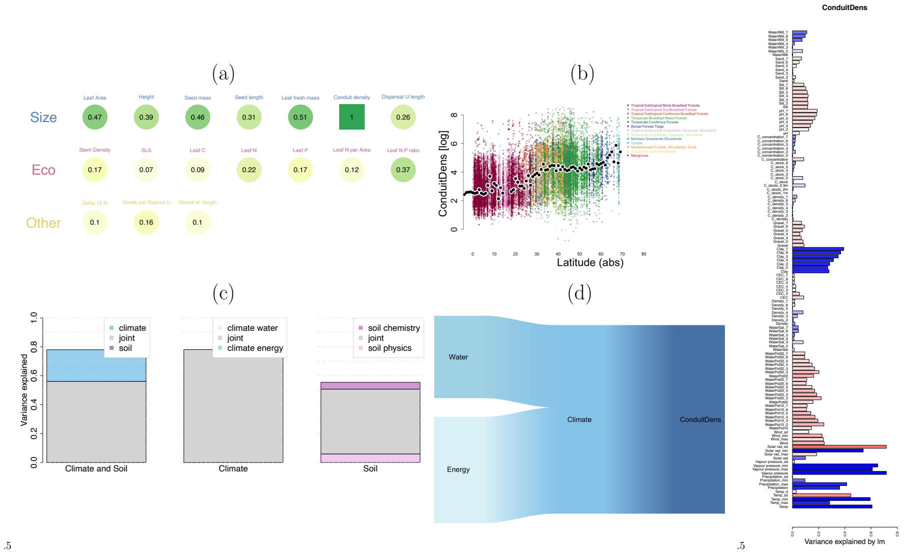

Figure 24 – Conduit density, size trait.

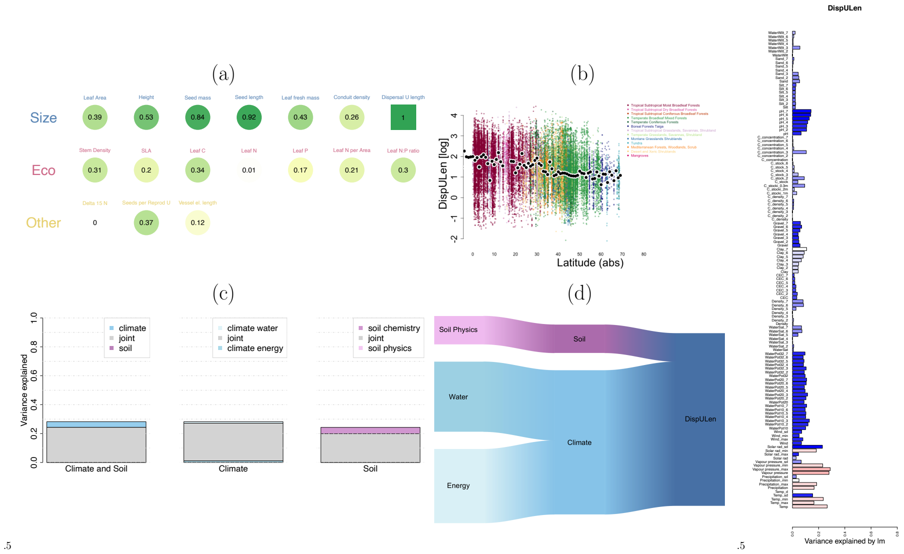

Figure 25 – Dispersal unit length (Dispersal U length), size trait.

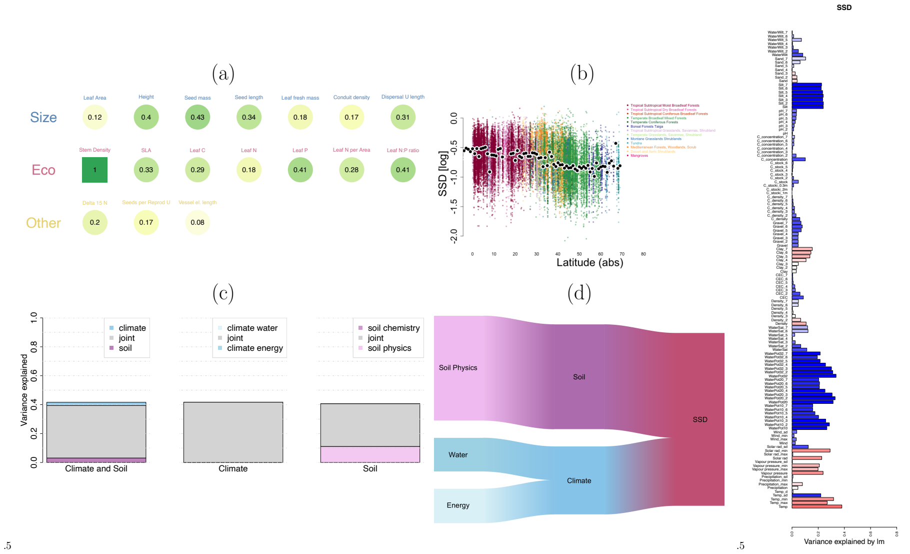

Figure 26 – Stem specific density, economics trait.

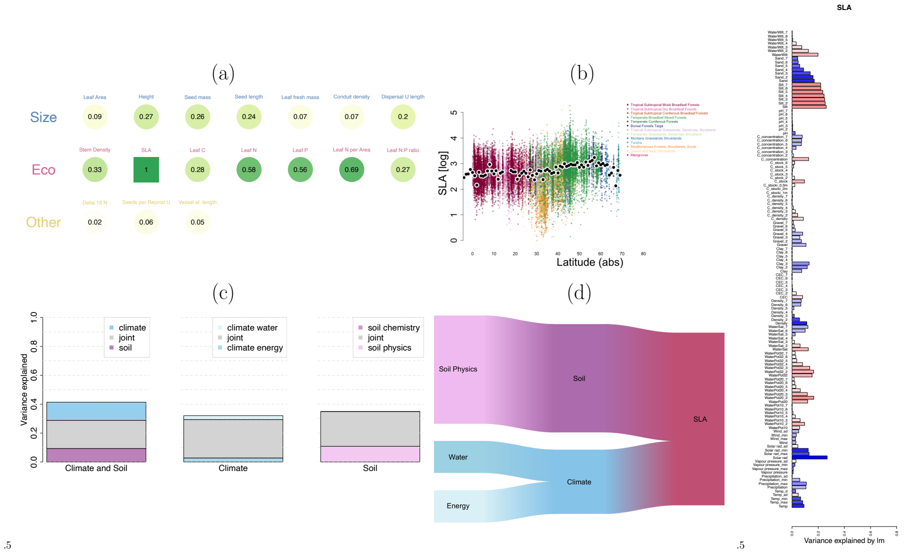

Figure 27 – Specific leaf area (SLA), economics trait.

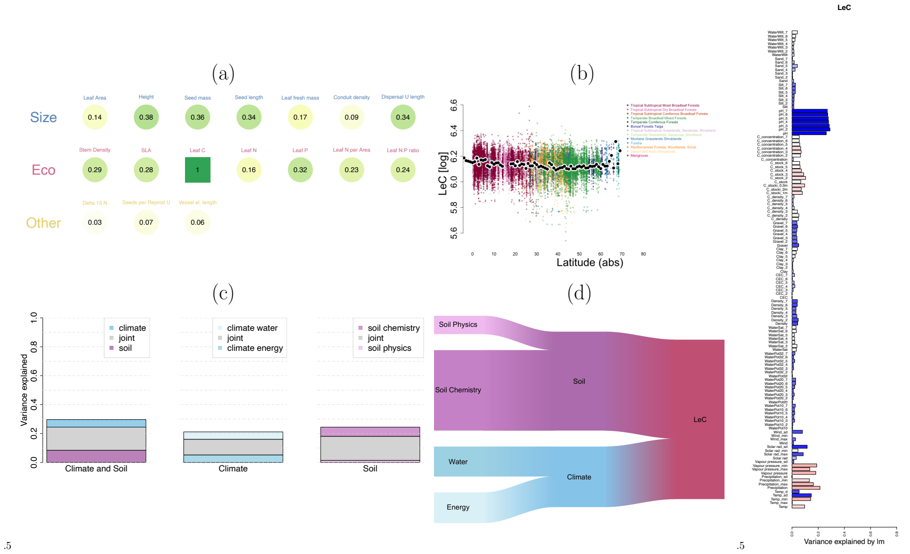

Figure 28 – Leaf carbon per leaf dry mass (leaf C), economics trait.

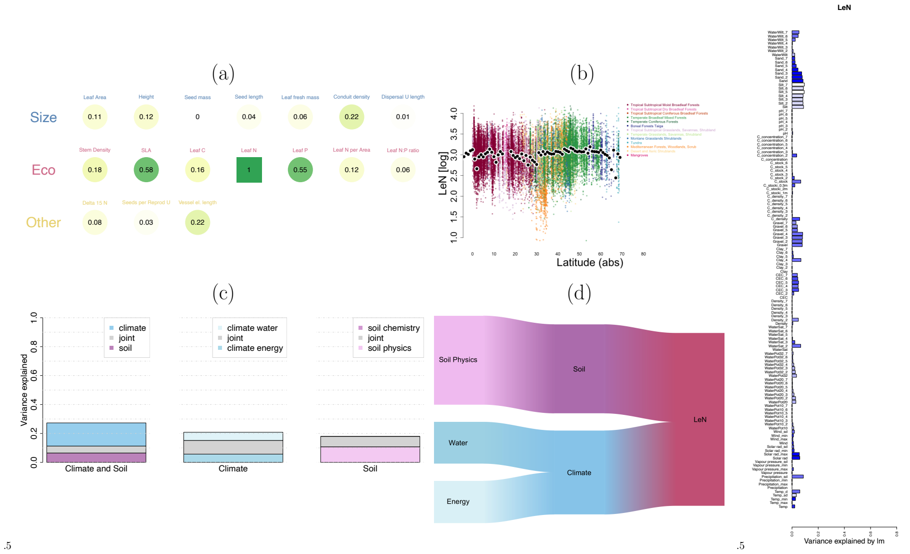

Figure 29 – Leaf nitrogen per leaf dry mass (leaf N), economics trait.

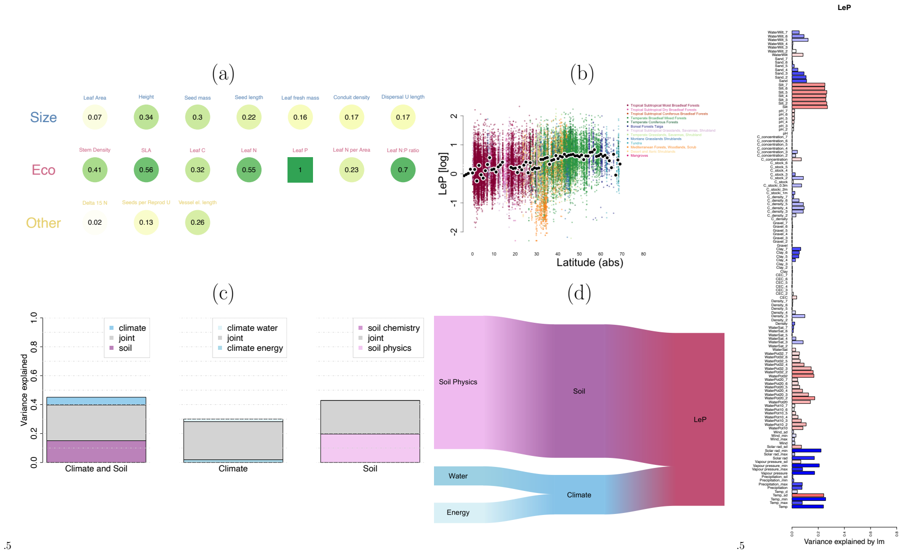

Figure 30 – Leaf phosphorous per leaf dry mass (leaf P), economics trait.

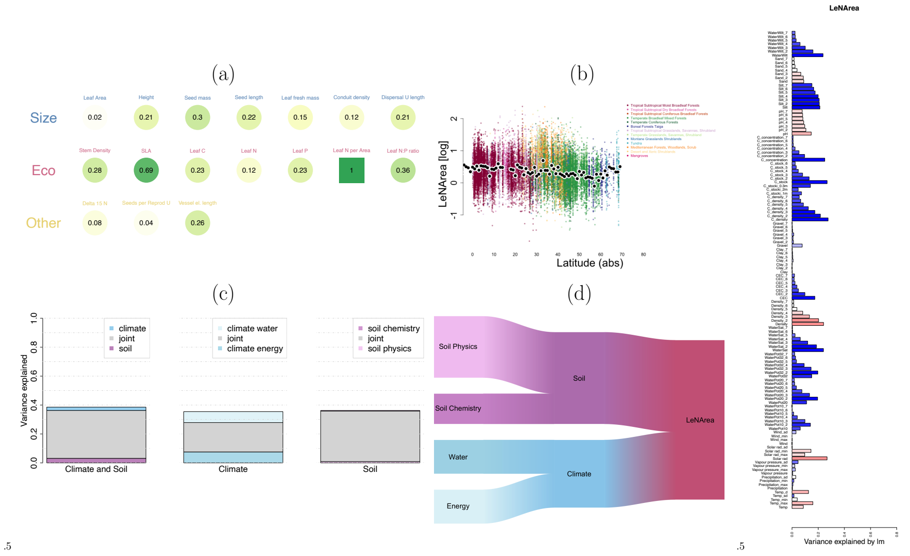

Figure 31 – Leaf nitrogen per leaf area (leaf N per Area), economics trait.

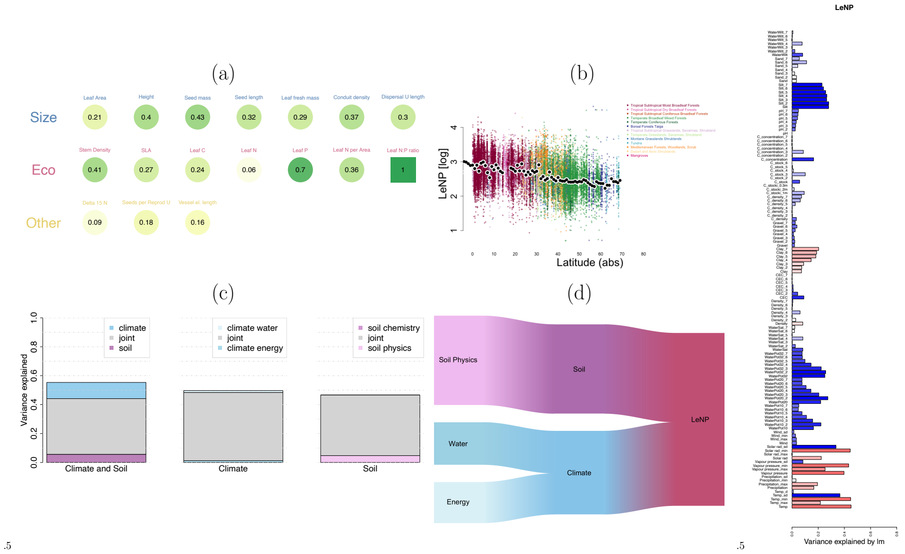

Figure 32 – Leaf nitrogen to phosphorous ratio (leaf N:P ratio), economics trait.

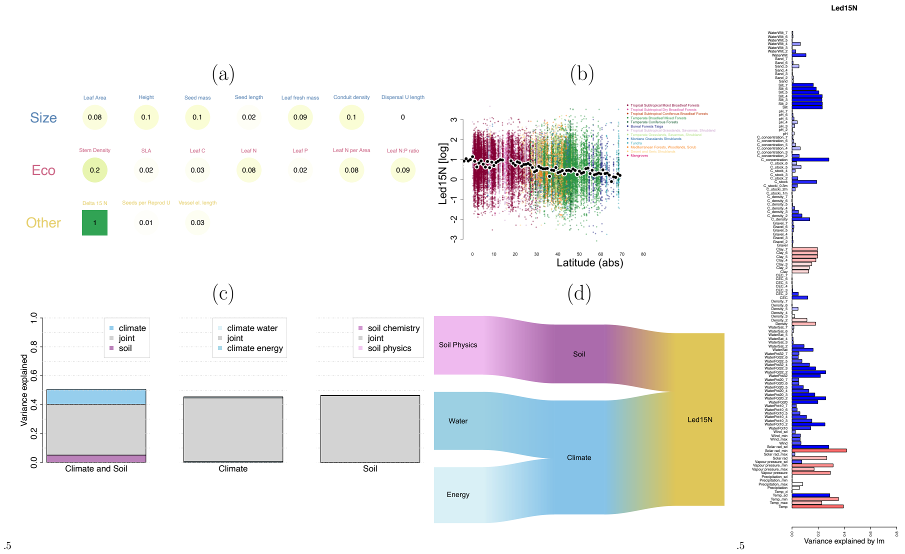

Figure 33 – Leaf delta 15N (Delta 15 N), other trait.

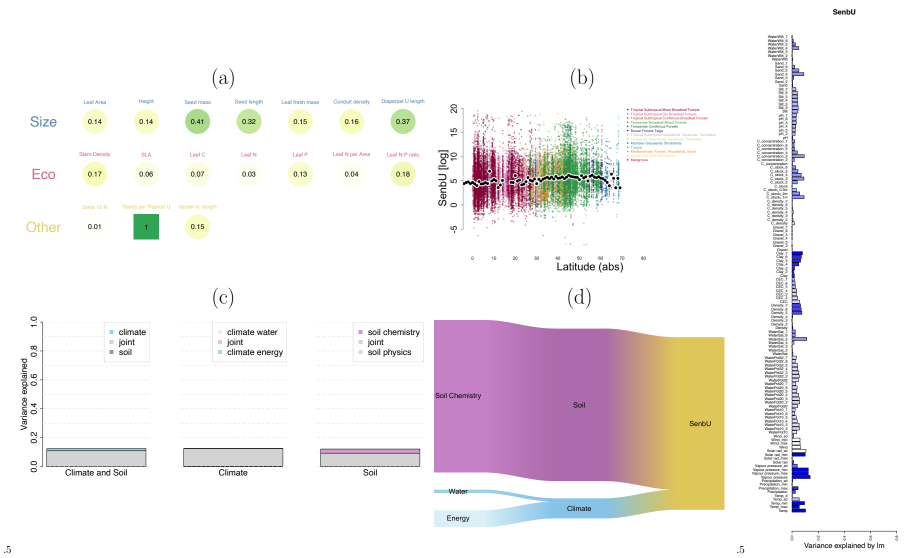

Figure 34 – Seeds per reproduction unit (Seeds per Reprod U), other trait.

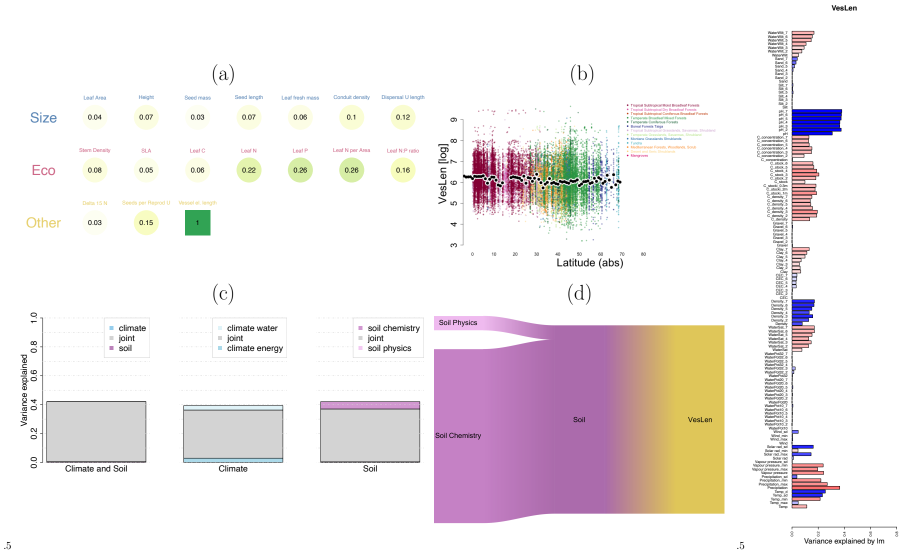

Figure 35 – Vessel element length (Vessel el. length), other trait.

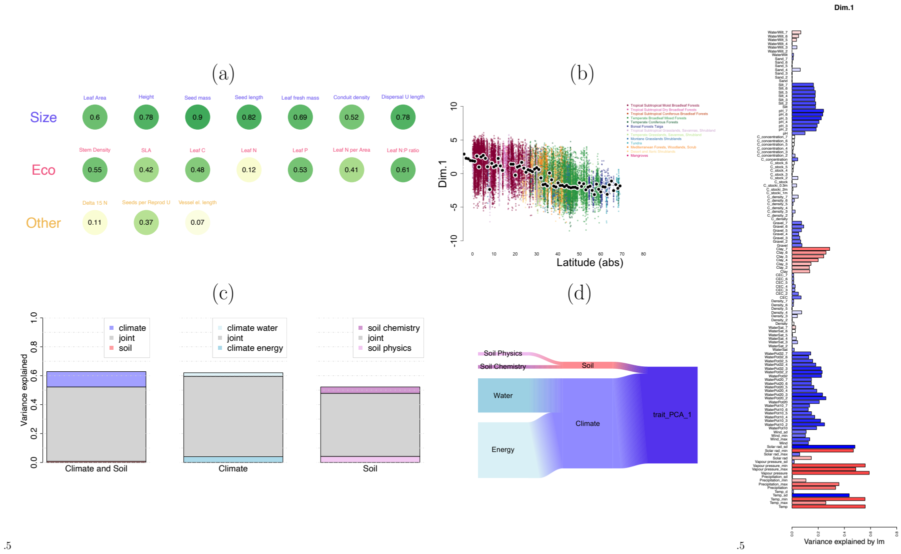

Figure 36 – PC1, referred to as size trait bundle.

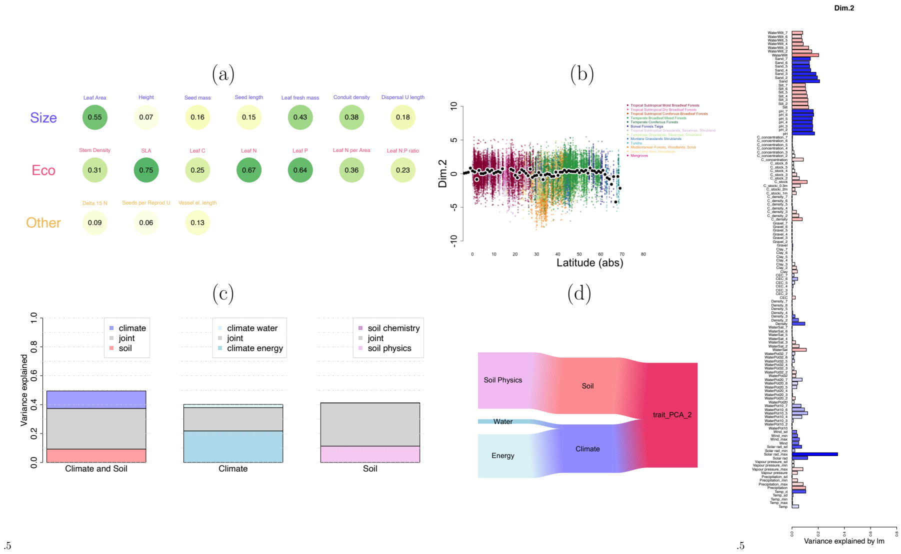

Figure 37 – PC2, referred to as economics trait bundle.

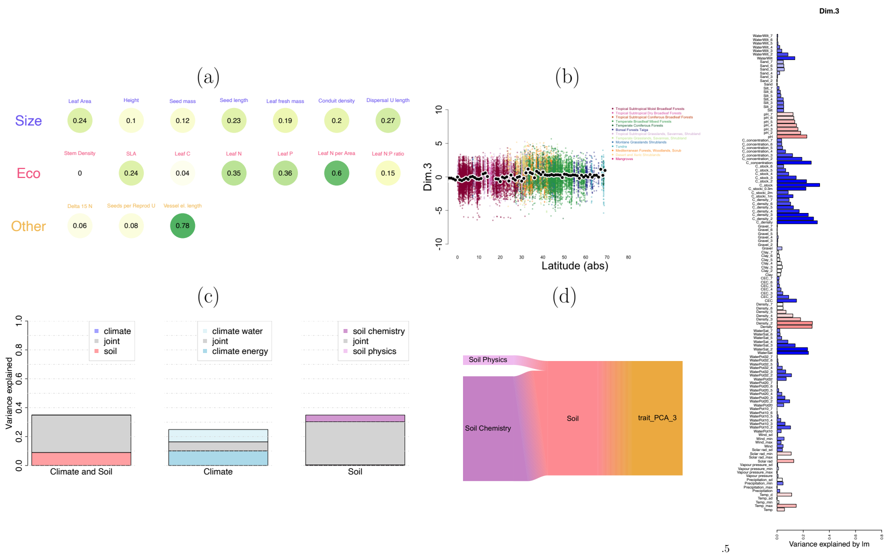

.5 .5

Figure 38 – PC3, explains 9.36% of 17 plant traits (species median scale).

8.1 Redundancy analysis (RDA) with forward selection

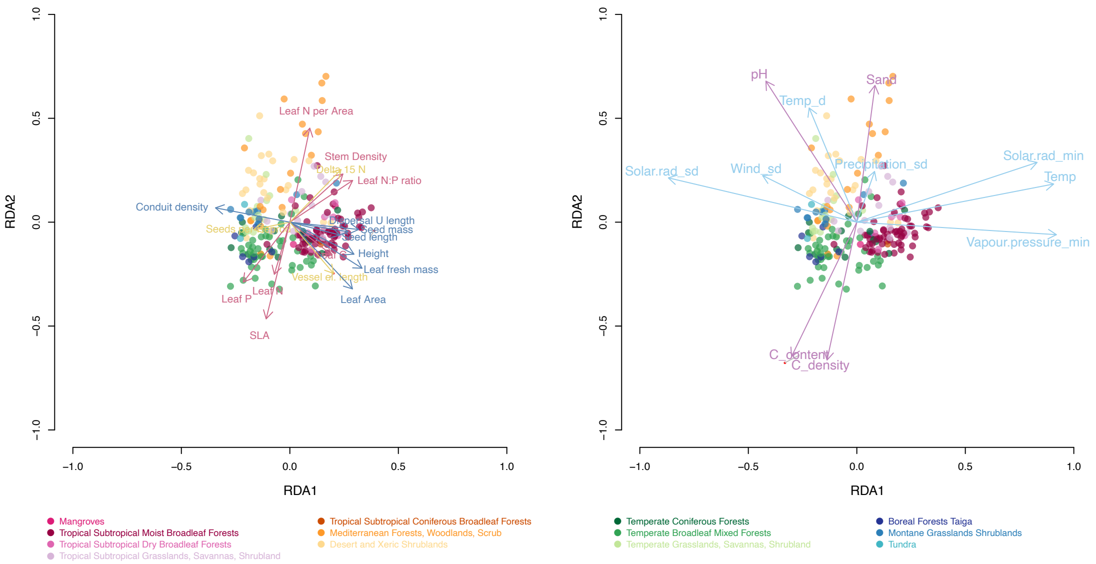

Figure 39 – Redundancy analysis (RDA) of traits (n=220, ecoregional median, only top soil layer variables included). Environmental variables were selected based on an RDA model stepwise forward selection with the Akaike criterion. The output of the RDA is split into two plots: (left) traits, colored according to trait groups (blue=size traits, red=economics traits, yellow=other traits), and (right) environmental factors (climate=blue and soil=red variables); points represent ecoregions, colored according to biome (red=tropics, green=temperate, yellow=desert, orange=mediterranean, dark blue=tundra). Climate variable abbreviations: Solar.rad_sd=seasonality of solar radiation, Wind_sd=seasonality of wind speed, Temp_d=diurnal temperature range, Precipitation_sd=seasonality of precipitation, Solar.rad_min=minimum annual solar radiation, Tem=mean annual temperature, Vapour.pressure_min=minimum annual vapour pressure. Soil variable abbreviations: pH=topsoil pH, Sand=topsoil sand fraction [vol%], C_content=topsoil carbon content, C_density=topsoil carbon density.

9 The origin of the dip at the latitudinal gradient of PC2

First, we investigated whether only certain biomes (and thus climatic conditions) were most affected by the apparent deviation from a linear relationship between PC2 and latitude (Figure 2). We find Mediterranean, Desert, Tundra and Montane grassland biomes to drive the dip (Supplementary Fig. 40). We then investigated environment - PC2 relationships at the relevant latitudes (Supplementary Fig. 41), and found a combined effect

of climate and soil causing low water availability and thus possibly a change in PC2 and economics traits.

9.1 Biomes at the Dip

PC2 was subset to the latitudes which show the strongest deviation (29° to 36°). In Supplementary Fig. 40, a boxplot of each biome and its median (blue line) are compared to the median of this latitudinal PC2 subset (black dashed line). The Mediterranean biome shows particularly large (negative) divergence to the mean, both in spread and in median.

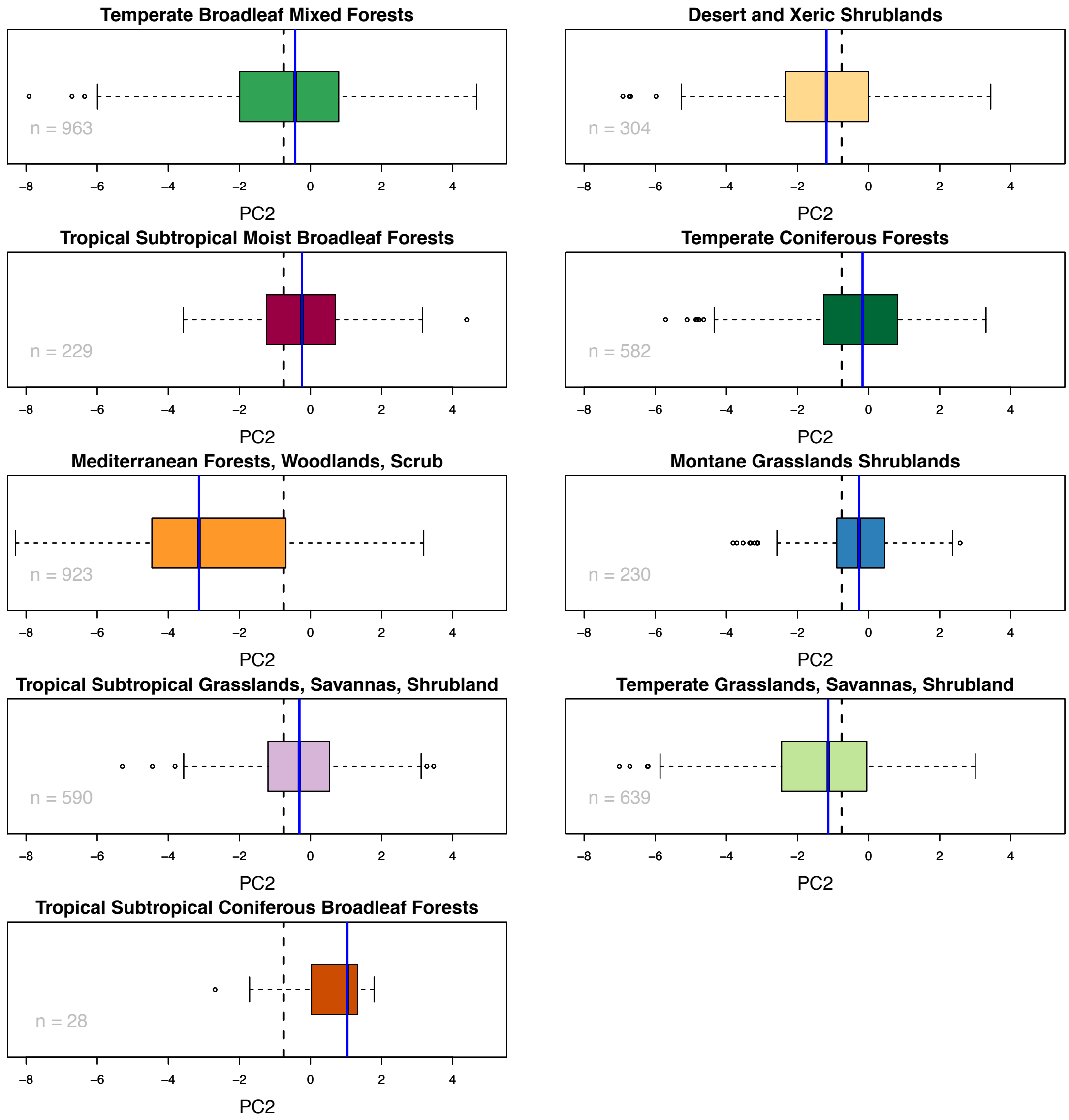

Figure 40 – Boxplots of PC2 values of the latitude subset of the dip (29° to 36°). Biome median in blue, median of this latitudinal PC2 subset in black (dashed line).

9.2 Combined effect of climate and soil at the dip

To attribute the effect to environmental variables, we searched qualitatively for PC2-environment gradients. A pre-study showed the sand fraction and precipitation of the driest month to be relevant variables for PC2. Supplementary Fig. 41 shows the combined effect of the high sand fraction and low precipitation, both reducing the water availability. With a sand fraction above 55%, relative values of PC2 decrease. This dip appears to be buffered by high minimum precipitation. As a comparison we show the same analysis for PC1, which lacks this gradient.

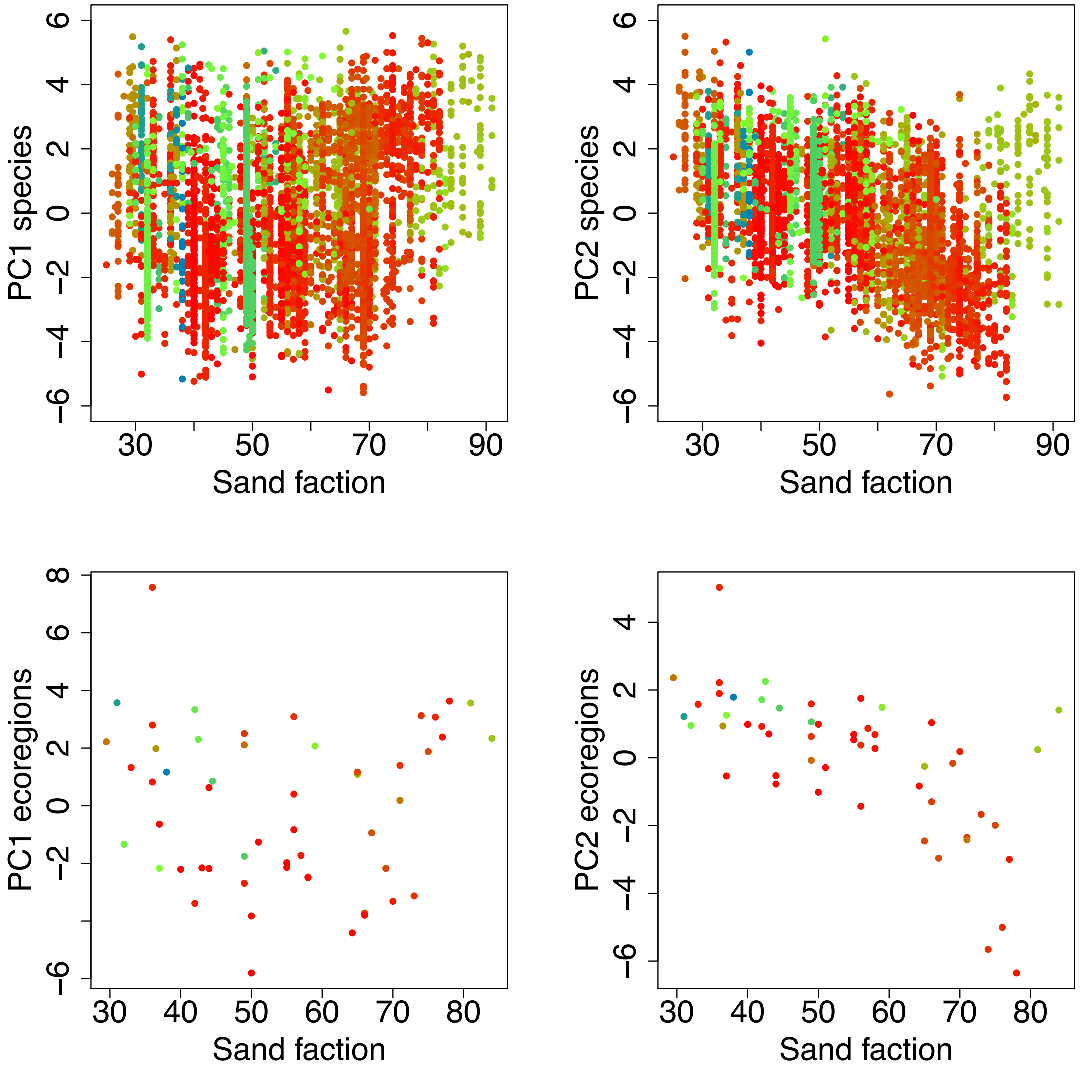

Figure 41 – A combined effect of climate and soil as one possible reason for variation in economics traits (towards long-lived species). The figure displays a subset of the trait PC1 and PC2 from latitudes 29° to 36° against the sand fraction on the aggregation levels of unique ecoregion species (n=4,488) and ecoregions ($n_{ecoregions}$=53). Colors refer to minimum precipitation (red=low precipitation, green=high precipitation). The subset degrees refer to the dip of PC2, onto which mainly the economics traits load (Figure 2).

10 Explained variance of size and economics traits depend on their coefficient of variation.

We evaluated the intrinsic difference of size vs economics trait variation within ecoregions, and found economics traits to vary more internally than size traits (Supplementary Fig. 42).

The coefficient of variation was calculated from the mean of trait standard deviations per ecoregion (log 10 transformed)) against standard deviation of trait means per ecoregion (log 10 transformed)).

$$\frac{mean(sd(trait_{ecoregion}))}{sd(mean(trait_{ecoregion})}$$

Supplementary Fig. 42 shows a negative relationship between variance explained by all environmental variable inputs and the coefficient of variation, yet when looking at the size traits alone the inverse is true. Overall, economics traits vary more than size traits.

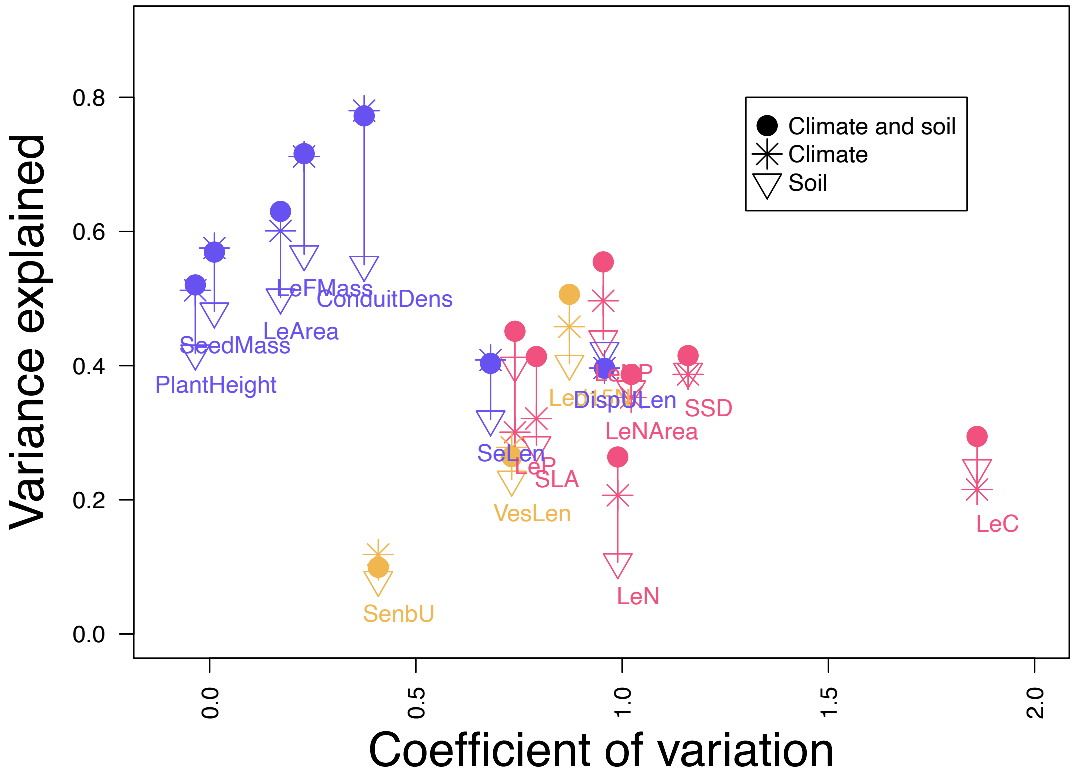

Figure 42 – Explained variance from climate and soil versus coefficient of variation.

11 Data information

11.1 Climate data

Table 1 – Climate variables used for the analysis (n=21). The data were derived from WorldClim at the scale of 1km^2 (worldclim.org).

11.2 Soil data

Table 2 – Soil variables (most with 7 depths) used for the analysis (n=107). The data were derived from SoilGrids[4] at the scale of 1km2, hosted at the ISRIC - World Soil Information (isric.org).

11.3 Trait data

Table 3 – Attribution of soil and climate variables to subgroups for the single trait analyses (Supplementary Fig. 19 to Supplementary Fig. 38).

Table 4 – Independent and joint effect of latitude and climate or soil derived from ridge regression and hierarchical partitioning. Median for all traits, and split into size or economics traits. Analysis based on ridge regression and hierarchical partitioning. Unit is % of explained variance (r^2) by ridge regression model.

Table 5 – Table describing the trait information of the data on 17 traits used for this study. This data has been extracted from a BHPMF gap-filled version of a larger trait set data (Supplementary Tab. 6,Supplementary Tab. 6). The table includes the original trait name and ID as used in the TRY data set (try-db.org), the abbreviation as used in this study, trait units, and the original number of observations in the data prior to gap-filling.

11.4 Trait data for gap-filling

Table 6 – Trait information of the trait data set used for BHPMF gap-filling (for details see methods). This data set contains observed values of 172 traits and 652,957 individuals. The table includes trait name and number of individual samples. Traits that entered the analysis of this study appear in bold and in Supplementary Tab. 5.

11.5 Ecoregion

Table 7 – Ecoregion[6] information including ecoregions' name and ID, the number of species and the number of observations used in this study, an estimate of plant species richness[5] , location of the ecoregions' central point (longitude and latitude) with maximum and minimum latitude of observation locations included, additionally to the ecoregions' extent as area.

Table 8 – Table showing for each trait the variance explained (r^2) by ridge regression models for 220 ecoregions as in Table 1. Moreover the independent effects for climate and soil are listed from hierarchical partitioning that respectively add up with the joint effect to the variance explained by climate or soil. Mean values including minimum and maximum values from different cross validation runs in brackets.

References

[1] Bjorkman, A. D. *et al.* Plant functional trait change across a warming tundra biome. *Nature* **562** (2018).

[2] Chevan, A. & Sutherland, M. Hierarchical Partitioning. *The American Statistician* **45**, 90–96 (1991). URL `http://www.tandfonline.com/doi/abs/10.1080/00031305.1991.10475776`.

[3] Díaz, S. *et al.* The global spectrum of plant form and function. *Nature* **529**, 1–17 (2015). URL `http://dx.doi.org/10.1038/nature16489`.

[4] Hengl, T. *et al.* SoilGrids1km â Global Soil Information Based on Automated Mapping. *PLoS ONE* **9**, e105992 (2014).

[5] Kier, G. *et al.* Global patterns of plant diversity and floristic knowledge. *Journal of Biogeography* **32**, 1107–1116 (2005).

[6] Olson, D. M. *et al.* Terrestrial Ecoregions of the World : A New Map of Life on Earth. *BioScience* **51**, 933–938 (2001).

[7] Hastie,T & Tibshirani, R. & Friedman, J.. *The Elements of Statistical Learning* (Springer, 2008), 2nd edn.
