# Supplementary material for: Climatic and soil factors explain the two-dimensional spectrum of global plant trait variation
Source: Nat Ecol Evol. 2021 Dec 23;6(1):36–50. doi: 10.1038/s41559-021-01616-8 (PMC8752441; doi:10.1038/s41559-021-01616-8)
Supplement: Supplementary file 2 — Reporting Summary [file 41559_2021_1616_MOESM2_ESM.pdf]

nature portfolio

Corresponding author(s): Julia Joswig

Last updated by author(s): Nov 1, 2021

Reporting Summary

Nature Portfolio wishes to improve the reproducibility of the work that we publish. This form provides structure for consistency and transparency in reporting. For further information on Nature Portfolio policies, see our Editorial Policies and the Editorial Policy Checklist.

Statistics

For all statistical analyses, confirm that the following items are present in the figure legend, table legend, main text, or Methods section.

n/a	Confirmed	
☐	☒	The exact sample size (*n*) for each experimental group/condition, given as a discrete number and unit of measurement
☐	☒	A statement on whether measurements were taken from distinct samples or whether the same sample was measured repeatedly
☐	☒	The statistical test(s) used AND whether they are one- or two-sided<br>*Only common tests should be described solely by name; describe more complex techniques in the Methods section.*
☐	☒	A description of all covariates tested
☐	☒	A description of any assumptions or corrections, such as tests of normality and adjustment for multiple comparisons
☐	☒	A full description of the statistical parameters including central tendency (e.g. means) or other basic estimates (e.g. regression coefficient) AND variation (e.g. standard deviation) or associated estimates of uncertainty (e.g. confidence intervals)
☐	☒	For null hypothesis testing, the test statistic (e.g. F, t, r) with confidence intervals, effect sizes, degrees of freedom and P value noted<br>*Give P values as exact values whenever suitable.*
☐	☒	For Bayesian analysis, information on the choice of priors and Markov chain Monte Carlo settings
☐	☒	For hierarchical and complex designs, identification of the appropriate level for tests and full reporting of outcomes
☐	☒	Estimates of effect sizes (e.g. Cohen's d, Pearson's r), indicating how they were calculated

Our web collection on statistics for biologists contains articles on many of the points above.

Software and code

Policy information about availability of computer code

Data collection	Trait data: TRY (www.try-db.org, accession date July 2017, request nb.3282)<br>Climate data: WorldClim is publicly available via https://www.worldclim.org/ (accession date: May 2018),<br>Soil data: SoilGrids (soilgrids.org, accession date: June 2018) is publicly available<br>Ecoregions: of Olson et al. 2001 Shapefiles are publicly available (accession date January 2014, https://www.sciencebase.gov/catalog/item/508fece8e4b0a1b43c29ca22)<br>Estimate of species richness per ecoregion: Kier species richness (accession date January 2014, https://databasin.org/datasets/43478f840ac84173979b22631c2ed672/)<br>Spatial data (licenced, see third party rights form)
Data analysis	R, R-Studio, Microsoft Excel

For manuscripts utilizing custom algorithms or software that are central to the research but not yet described in published literature, software must be made available to editors and reviewers. We strongly encourage code deposition in a community repository (e.g. GitHub). See the Nature Portfolio guidelines for submitting code & software for further information.

Data

Policy information about availability of data

All manuscripts must include a data availability statement. This statement should provide the following information, where applicable:

- Accession codes, unique identifiers, or web links for publicly available datasets
- A description of any restrictions on data availability
- For clinical datasets or third party data, please ensure that the statement adheres to our policy

Plant trait data were accessed from the TRY data base (try-db.org, request number: 3282, accession date: July 2017, see also Figure \ref{fig_extDat}). All TRY data required to reproduce this analysis, and the corresponding R-scripts, are provided in an open TRY File Archive (https://www.try-db.org/TryWeb/Data.php). Climate data WorldClim is publicly available via https://www.worldclim.org/ (accession date: May 2018), Soil data, namely SoilGrids (soilgrids.org, accession date: June 2018) is publicly available. Ecoregion information \cite{Olson2001} shapefiles are publicly available (accession date January 2014, https://www.sciencebase.gov/catalog/item/508fece8e4b0a1b43c29ca22), The estimate of species richness per ecoregion (Kier et al. 2005) is publicly available (accession date January 2014, https://databasin.org/datasets/43478f840ac84173979b22631c2ed672/. Data for this study can be accessed on Github (https://github.com/juliajoswig/ Repo\ClimateSoil\TraitSpectrum).
For the extended data figure 1, Supplementary Fig.7 , the Geodata product of the Missions Database "ArcWorld Supplement" (GMI) was used, published by Global Mapping International and originated from Global Mapping International for producing and available in ArcGIS software by Esri. ArcGIS and ArcMapTM are the intellectual property of Esri and are used herein under license. Copyright Esri. All rights reserved. For more information about Esri software, please visit www.esri.com.

Field-specific reporting

Please select the one below that is the best fit for your research. If you are not sure, read the appropriate sections before making your selection.

☐ Life sciences ☐ Behavioural & social sciences ☒ Ecological, evolutionary & environmental sciences

For a reference copy of the document with all sections, see nature.com/documents/nr-reporting-summary-flat.pdf

Ecological, evolutionary & environmental sciences study design

All studies must disclose on these points even when the disclosure is negative.

Study description

The overarching objective of this study is to test whether the major dimensions underpinning the global spectrum of plant variation, such as the size and leaf economics spectra, are the result of joint and/or independent variation of climate and soil.
For this study, we compiled and analyze a dataset of 17 functional traits for 225,206 geo-referenced observations comprising records of 20,655 global unique species (36,197 unique species to ecoregion combinations) covering the main worlds' ecoregions – environmentally homogeneous areas characterized by distinct plant assemblages (Methods, Extended Data Figure 1). The trait data were complemented with 21 climate variables (Supplementary Tab. 1) as well as 107 soil variables (Supplementary Tab. 2). Trait-environment relationships were analyzed on the basis of ecoregions using a combination of a regression technique and hierarchical partitioning (for details see Methods).

Research sample

We extracted data of 17 plant functional traits from the TRY data base1 (Supplementary Tab. 5, Supplementary Tab. 6, www.try-db.org, accession date July 2017, request nb.3282) including published literature (see manuscript). To be able to include the maximum number of species in our analyses, we used the gap-filled version of TRY, with missing data were imputed using a Bayesian Hierarchical Probabilistic Matrix Factorization (BHPMF) algorithm, but observed values were kept. We excluded observations that were not geo-referenced. In total, we included 225,206 observations from 20,655 global unique species (36,197 unique species to ecoregion combinations). The data were stratified by ecoregions (see below), aggregated to species median values per ecoregion and log-transformed.

To represent climate conditions we used 21 variables derived from the WorldClim reanalysis product at a resolution of 1 km (accession date May 2018). We extracted values for temperature (annual average, diurnal range, max of warmest month, min of coldest month, sd), precipitation (annual average, min, max, sd), vapour pressure (annual average, min, max, sd), solar radiation (annual average, min, max, sd) and wind (annual average, min, max, sd; see Supplementary Tab. 1).

To characterise soil conditions we used 107 variables derived from the ISRIC data product 'SoilGrids' (soilgrids.org through ISRIC - WDC Soils, Supplementary Tab. 2). 'SoilGrids' provides global predictions of soil characteristics for seven depths, i.e. 0, 5, 15, 30, 60, 100, 200 cm at a resolution of 1 km.
In total, we included 128 environmental variables. Before performing the ridge regression, climate and soil variables were reduced to 20 variables each by means of a principle component analysis (PCA).

Sampling strategy

The data were stratified by ecoregions, aggregated to species median values per ecoregion and log-transformed. To determine trait-environment relationships, we aggregated trait as well as environmental data to ecoregions (Supplementary Tab.7) Ecoregions are environmentally homogeneous areas characterized by distinct plant assemblages. At global scale Olson et al. defined 867 ecoregions. For each of the 867 ecoregions, we calculated the median of all species median trait values. For further analyses we only included regions with >20 species and a representation of >1% of the estimated species richness of the ecoregion.
To aggregate environmental variables to ecoregion level, we associated each geo-referenced trait observation with its corresponding values of climate and soil variables. Then, we averaged over all values within one ecoregion. Thus, the selected environmental

	variables represent averages that are weighted by the number and location of trait observations within ecoregions.
Data collection	Trait data was collected in the framework of TRY (Kattge et al. 2011).
Timing and spatial scale	Trait-data: geo-referenced global point data<br>Climate- data: gridded data (1km resolution)<br>Soil: gridded data of up to 7 different depths (1km resolution)
Data exclusions	For trait observations, we only included geo-referenced data.<br>For environmental data, we only included the grid-level information if a trait data point was located there.<br>For ecoregions we only included regions with >20 species and a representation of >1% of the estimated species richness of the ecoregion.
Reproducibility	All attempts to repeat the results were successful.<br>Per default, the ridge regression was repeated 50 times for each trait. The mean, minimum and maximum of r2 and independent effect give an estimate of model spreads (Table 1, Supplementary Tab. 9).<br>All TRY data required to reproduce this analysis, and the corresponding R-scripts, are provided in an open TRY File Archive (https://www.try-db.org/TryWeb/Data.php).
Randomization	To estimate how much of the ecoregion trait variation (r2) is explained by random environmental variables, we performed the ridge regression followed by hierarchical partitioning (see methods „ridge regression" and "hierarchical partitioning") and paired climate or soil with noise. The noise data set comprised randomly sampled values for a variable set, as large as the soil variable set (n=107). We performed ridge regression analysis with noise data, together with climate or soil. Then we calculated the independent effect of noise from climate or soil data.<br>The independent effect of randomized data (noise) is never above 0 (negative values due to large difference between r2_total and r2_noise and model variabiliy; Supplement Fig 2).<br><br>Moreover, we sampled for each ecoregion the minimum requirement for selection (Kier species richness of 1% and 20 species) and erased randomly (n=3) the rest of the species observations. See Supplement Fig. 2.
Blinding	*Describe the extent of blinding used during data acquisition and analysis. If blinding was not possible, describe why OR explain why blinding was not relevant to your study.*

Did the study involve field work? ☐ Yes ☒ No

Reporting for specific materials, systems and methods

We require information from authors about some types of materials, experimental systems and methods used in many studies. Here, indicate whether each material, system or method listed is relevant to your study. If you are not sure if a list item applies to your research, read the appropriate section before selecting a response.

Materials & experimental systems

n/a	Involved in the study
☒	☐ Antibodies
☒	☐ Eukaryotic cell lines
☒	☐ Palaeontology and archaeology
☒	☐ Animals and other organisms
☒	☐ Human research participants
☒	☐ Clinical data
☒	☐ Dual use research of concern

Methods

n/a	Involved in the study
☒	☐ ChIP-seq
☒	☐ Flow cytometry
☒	☐ MRI-based neuroimaging
