# Supplementary material for: Climatic and soil factors explain the two-dimensional spectrum of global plant trait variation
Source: Nat Ecol Evol. 2021 Dec 23;6(1):36–50. doi: 10.1038/s41559-021-01616-8 (PMC8752441; doi:10.1038/s41559-021-01616-8)
Supplement: Supplementary file 3 — Peer Review Information [file 41559_2021_1616_MOESM3_ESM.pdf]

natureresearch

Peer Review Information

Journal: Nature Ecology & Evolution
Manuscript Title: Climatic and soil factors explain the two-dimensional spectrum of global plant trait variation
Corresponding author name(s): Julia S. Joswig

Editorial Notes:

Reviewer Comments & Decisions:

Decision Letter, initial version:

17th March 2021

*Please ensure you delete the link to your author homepage in this e-mail if you wish to forward it to your co-authors.

Dear Ms Joswig,

Your manuscript entitled "Climatic and soil factors explain the two-dimensional spectrum of global plant trait variation" has now been seen by three reviewers, whose comments are attached. The reviewers have raised a number of concerns which will need to be addressed before we can offer publication in Nature Ecology & Evolution. We will therefore need to see your responses to the criticisms raised and to some editorial concerns, along with a revised manuscript, before we can reach a final decision regarding publication.

We therefore invite you to revise your manuscript taking into account all reviewer and editor comments. Please highlight all changes in the manuscript text file.

We are committed to providing a fair and constructive peer-review process. Do not hesitate to contact us if there are specific requests from the reviewers that you believe are technically impossible or unlikely to yield a meaningful outcome.

When revising your manuscript:

* Include a “Response to reviewers” document detailing, point-by-point, how you addressed each reviewer comment. If no action was taken to address a point, you must provide a compelling argument. This response will be sent back to the reviewers along with the revised manuscript.

* If you have not done so already please begin to revise your manuscript so that it conforms to our Article format instructions at http://www.nature.com/natecolevol/info/final-submission. Refer also to any guidelines provided in this letter.

* Include a revised version of any required reporting checklist. It will be available to referees (and, potentially, statisticians) to aid in their evaluation if the manuscript goes back for peer review. A revised checklist is essential for re-review of the paper.

Please use the link below to submit your revised manuscript and related files:

[REDACTED]

Note: This URL links to your confidential home page and associated information about manuscripts you may have submitted, or that you are reviewing for us. If you wish to forward this email to co-authors, please delete the link to your homepage.

We hope to receive your revised manuscript within four to eight weeks. If you cannot send it within this time, please let us know. We will be happy to consider your revision so long as nothing similar has been accepted for publication at Nature Ecology & Evolution or published elsewhere.

Nature Ecology & Evolution is committed to improving transparency in authorship. As part of our efforts in this direction, we are now requesting that all authors identified as 'corresponding author' on published papers create and link their Open Researcher and Contributor Identifier (ORCID) with their account on the Manuscript Tracking System (MTS), prior to acceptance. ORCID helps the scientific community achieve unambiguous attribution of all scholarly contributions. You can create and link your ORCID from the home page of the MTS by clicking on 'Modify my Springer Nature account'. For more information please visit please visit www.springernature.com/orcid.

Please do not hesitate to contact me if you have any questions or would like to discuss these revisions further.

We look forward to seeing the revised manuscript and thank you for the opportunity to review your work.

[REDACTED]

Reviewer expertise:

Reviewer #1: Plant traits

Reviewer #2: Biogeography of plant traits

Reviewer #3: Biogeography methods, gap-filling

Reviewers' comments:

Reviewer #1 (Remarks to the Author):

The manuscript 'Climatic and soil factors explain the two-dimensional spectrum of global plant trait variation' continues and brings together recent work to interrogate global plant trait variation.

Since the publication of Dıaz and colleagues 2015 paper identifying two major axes of global trait space (usually simplified to plant size and plant economics), a range of authors have sought to test and apply the conclusions, particularly in order to better understand how global change drivers may restructure ecosystems around the globe.

This manuscript neatly addresses a key unknown within this field of endeavour: whether axes of trait variation correspond to environmental gradients at the global scale, either independently or jointly. This is the first instance I am aware of that brings together both multiple traits and multiple environmental drivers within a global framework. This is critical to better understand how changes to environmental variables, either among ecosystems or over time, drive changes in multivariate trait space – and by extension plant community composition, and ecosystem function.

Joswig and colleagues find that the two main axes of trait variation are influenced by different environmental drivers. Traits relating to plant size exhibit a clear latitudinal gradient, which is well explained by climatic factors such as energy and water limitation. In contrast, economic traits do not exhibit a latitudinal gradient, and are better explained by soil characteristics. Together, these interacting drivers of trait expression present a framework to bring together individual trait-environment relationships and provide insight into plant life-history strategies within and among ecosystems.

On the whole, I feel that this is a well written paper, based on a substantial global trait database, with a robust set of analyses to support findings. Nevertheless, I have a number of concerns that primarily relate to the clarity and novelty of the findings, which at present limit the significance of these findings, particularly to those from other disciplines. My major concerns are set out below.

1. Questions and hypotheses.

I did not find that the paper set out clearly enough what is was aiming to test. For example, the overall hypothesis, we hypothesize that global patterns of trait correlation should closely follow gradients of climate and soil properties, is neither immediately intuitive nor particularly testable, particularly to those unfamiliar with this field.

In contrast, the questions underpinning this hypothesis statement are clearer: (1) to what extent the major dimensions underpinning the global spectrum of plant form and function can be attributed to global gradients of climate and soil conditions, and (2) to what extent these factors can jointly or independently explain the global spectrum of form and function (though I feel this is just an extension of the first question).

In my view, the manuscript also tests a second question in testing the robustness of the observed relationships using multiple traits, across multiple ecosystems, and for woody and non-woody species.

In essence, this analysis therefore brings together two major areas of study within plant trait literature, in a way that is currently underplayed.

a. Testing the two axes of global trait space (synthesis). The two axes of trait space have been tested and challenged in a number of ways since 2015. Those that I have seen (there may be others) include:
• That relationships do not hold for different traits (trait-dependent)
• That relationships do not hold for different plant types (group-dependent)
• That relationships do not hold in different ecosystems (ecosystem-dependent)
• That relationships do not hold at different scales (scale-dependent)

The authors demonstrate that the two axes are retained when analysis is increased from 6 to 17 traits, when performed for woody and non-woody species, and when performed with different combinations of species and ecoregions. Together, these analyses could offer a strong defence of the two axes, in a way that brings together key criticisms and potentially synthesises a number of individual previous studies using this strong global dataset. While that potential exists within the findings presented (including many in the supplementary materials), the wider relevance and implications of these tests is not well articulated. In part this may stem from weak recognition of the body of work performed since 2015 (see point 2).

b. Testing the utility of the two axes of global trait space, via relationship with environmental drivers (novel framework and analysis). If the two axes of traits variation are considered robust, a key question is therefore how this understanding may be applied; in the words of the authors, they have the potential to be "powerful predictors of plant community assembly and ecosystem functioning", particularly for global models of vegetation dynamics and land-climate feedbacks.

The authors note that there is a long history of study of the relationships between individual traits and environmental variables (p7). While not mentioned in the text, there has also been growing evidence that the two axes of trait variation are driven by different environmental variables, in some cases leading to unexpected results (see point 2 below). However, the authors are correct in noting that this is the first instance I am aware of that brings together both multiple traits and multiple environmental drivers within one framework.

This aspect of the study is well developed and relatively clearly articulated (in line with the question above), but would be strengthened by (1) a clearer hypothesis statement that specifically articulates a prediction, and (2) clearer articulation of why this question is of value – particularly to other disciplines.

2. Acknowledgement of previous work.

My second major criticism is that the manuscript currently does not sufficiently acknowledge and build on recent work to interrogate plant trait variation in the last five years. At times this reads as if the study is being published as an immediate follow-up to the 2015 work, rather than following 5-6 years of intense scrutiny and further investigation by numerous teams. This is relatively easy to address, and would build a stronger case for a need for the 'overall framework' set out in this manuscript. To name a few examples,

• Bruelheide and colleagues (NEE, 2017 Global trait–environment relationships of plant communities)

perform a global, plot-level analysis of trait–environment relationships, finding strong filtering of 17 functional traits but emphasising local filtering of traits
• Bjorkman and colleagues (Nature, Plant functional trait change across a warming tundra biome, 2018), examine biome-wide relationships between temperature, soil moisture and the same key traits as the Diaz (2015) study), empahsising the differential roles played by temperature and moisture on each trait
• Thomas and colleagues (NatComms, Global plant trait relationships extend to the climatic extremes of the tundra biome, 2020) perform a test of the two trait axes within tundra environments, finding that size traits but not economic traits are constrained by environmental conditions, "indicating that two of the major axes of global trait variation may be differentially selected by environmental conditions, and could thus respond differently to environmental change".
• A large number of other studies have tested trait axes at different scales, with different traits (e.g. root traits), in different ecosystems and sites, and with different functional groups.

I note this, not to take away from the novelty of findings, but to put them in context of the large body of work underway, that already points to the framework outlined here. I would disagree that "we lack general narratives describing these fundamental relationships at global scale" (for example, I would say warm and wet = big and tall; cold and/ or dry = small and hardy is a reasonably general narrative). However, I would agree that "examples have accumulated without an overall framework in which to place them". To me this is where the real strength of this study lies – an opportunity to place the emerging but disparate evidence of the findings articulated here into a robust and consistent framework.

3. Broader utility of findings.

Given the ambition of this analysis, I feel that it misses the opportunity to go further and be of greater interest and use to those working beyond plant ecology. The analysis provides quantitative relationships between PCA axes and latitude (which is interesting), but largely descriptive investigations of the relationships between PCA axes and environmental drivers, beyond their relative importance. However, if this work is to truly help to 'constrain parameters of global coupled climate-vegetation models ', I suspect this needs to go beyond latitude (which does not change) to quantify relationships between PCA axes and (interacting) environmental drivers.

Instead, the link with water and energy limitation is currently indirect: a relationship between trait PCA axes and latitude, and a relationship between latitude and energy / moisture, therefore a relationship between trait PCA axes and energy / moisture. A powerful next step would be to test – and quantify – the relationship between trait PCA axes and energy / moisture.

As examples, the variation explained by environmental drivers is set out in the manuscript both for PCA axes (main findings) and for individual traits (table 1), and to some extent the direction in PCAs, but these are not quantified in the same way as for latitude. The relationship between PCA axes and latitude is set out (Figure 2), but with not for key environmental drivers (or combinations of environmental drivers).
Although this may beyond the scope of this analysis, one suggestion may be to develop the multi-panel figures used for single traits (S20-37), but applied to PCA axes, with additional information provided for relationships with key environmental variables.

I would invite the authors to consider how this further step could be investigated and communicated in

this study, or at very least to set out how it could be developed further in future analyses.

4. Bias towards temperate / forest ecosystems

The authors set out concerns that using grid cells to scale results would be globally uneven in comparison to ecoregions, and a global sampling bias towards Europe. However, I am concerned that the filtering process applied to ecoregions to ensure sufficient data quality also results in a similar sampling bias, since ecoregions with sufficient data and more likely to come from well sampled regions. This is visible in Figure 4 and extended data fig 1.

Given very environmental conditions between ecoregions, the authors should do more to demonstrate that the relationships found here between traits environmental drivers are also consistent within ecoregions, and do not result from strong patterns once data has been aggregated at the ecoregion level or within the two-three most common ecoregion types (which appear to be Tropical Subtropical Moist Broadleaf Forests and Temperate Broadleaf Mixed Forests). A short metadata table to accompany the long list of ecoregions would also be helpful here.

5. General clarity

Given the scope and impact of the paper, the discussion would warrant significant shortening to focus on the key messages. At times the text becomes somewhat repetitive of similar overarching themes, and also focuses on certain detailed points which I am not sure are essential (e.g. aeolian and fluviatile sorting processes of glacial moraines).

Instead, the discussion could more succinctly could draw out the specific implications of findings. For example, might it be true to infer that climate warming might be expected to predominantly affect size-related traits, while anthropogenic alterations to soil conditions may affect economic traits? This is hinted at in the text, but this hypothesis would provide a great number of avenues for future work.

Specific comments:

"…environmental controls contributing to these two main axes. There is ample evidence that…"
Suggest you start a new paragraph here for clarity

"…climate and soils together shape plant form and function…"
Would it be possible to add a very short example here for the wider audience?

"we still lack general narratives describing these fundamental relationships at global scale"
I would disagree with this. I think we have general narratives, but lack a strong framework to explain these narratives.

"Rather, examples have accumulated without an overall framework in which to place them"
I agree with this, think this is the important point - we know the overall trends, but don't necessarily have a functional framework within to place these trends.

In other words, we have broad correlations at the global scale, and we have specific relationships for a multiple individual traits, but we do not necessarily have a framework to bring those two elements together within global multivariate trait space.

“Many of these traits show latitudinal patterns”
True, though it is not the latitudinal patterns we are interested in, per se, but rather the causal relationships behind these latitudinal patterns.

“Combining the insights that the global spectrum of plant traits comprises two internally correlated, orthogonal groups, and that many plant traits are individually linked to environmental gradients, we hypothesize that global patterns of trait correlation should closely follow gradients of climate and soil properties”
This is very confusing hypothesis sentence. I am not quite sure what the hypothesis that “patterns of trait correlation follow climate and soil gradients” means, nor immediately how you say it has been met / falsified.

“Therefore, we hypothesize that those climate (and soil) aspects that covary with latitude consistently determine size traits, while they have little effect on economics traits, which are more strongly affected by latitude-independent soil (and climate) effects.”
Ref Thomas et al, 2020 for a real world example of this.

“We investigate the power of climate and soil variables for predicting each of these traits”.
Given the potential importance of microclimate / local soil effects for economic traits identified in this study (but also Bruelheide et al, 2017), is there a risk that you cannot draw meaningful relationships between economic traits and soil variables at this relatively macro scale.

“Overall, size traits are better explained (r2=0.55; maximum r2=0.78 for conduit density) than are economics traits (r2=0.40; maximum r2=0.55 for Leaf N:P ratio).”
Neat

“The independent climate effects are ubiquitous across traits, but size traits tend to be better explained by the independent climate effects than are economics traits. In contrast, independent soil predictors are relevant for all economics traits - but not size traits (apart from a small contribution to leaf area).”
Ref Bjorkman et al, 2018 for a real-world example of this across climate gradients using plot-level data

“These two main trait groups remain clearly identifiable when the analysis is conducted separately for woody and non-woody species”
This is a very nice sensitivity analysis, though not clear to the general reader why this is needed or why it is relevant.

Discussion in general – suggest you think about how you break this up into paragraphs. Some key points are somewhat lost in the mass of text.

“Additional traits may add relevant axes of trait variation”
“I think this comment is too broad - after stating that the two axes hold for 17 traits, it doesn’t follow

to simply say that more traits may add more information. Rather, I think the value you can add, based on the analysis conducted in this manuscript, is to draw out what particular drivers of variation may not be picked up by the traits being tested…and therefore what traits you may predict we would wish to target to better develop in trait databases."

"Variation in size traits, represented by PC1 in Figure 1b, shows a clear latitudinal gradient."
While this is a neat finding, I am not sure it is the most important one in the context of this analysis. I am reminded of a comment on another manuscript, which used trait data to identify changes at treelines. The reviewer considered it to be a smart approach, but noted that it was not particularly novel to conclude that there were no trees north of the treeline. In other words, the broad trait trends with latitude identified here are interesting, and the approach (particularly looking across multiple size traits) is very good, but the conclusion – variation by latitude – is not especially groundbreaking.

In many places this analysis is pitched in terms of improving bioclimatic / specie envelope models, which care less about latitude and more about trait-environment relationships. Given that you have specifically looked at the relationship between the PCA axes and climatic variables (e.g. energy+moisture), I think this could provide a more novel outcome, which would have obvious implications for modelling impacts of climate change, for example.

"In addition to a decrease at high latitudes above 60 (absolute) where, however, species data become increasingly limited"
I notice this analysis does not include more recent (open access) high-latitude trait data from Bjorkman et al, 2019 – see Tundra Trait Team: A database of plant traits spanning the tundra biome. That dataset roughly doubled the previously available TRY trait data above 60 degrees, so would be of value here.

"At high latitudes, cold winters and short growing seasons demand more conservative nutrient-use strategies (like evergreen leaves) and protection against frost damage"
I would not fully agree with this, and regardless, this would not be relevant to size traits (which this discussion is focusing on). While tundra species are generally more conservative compared to the global mean, there is still widespread variation. For example, some tundra species (e.g. alpine forbs) also have highly opportunistic strategies to make use of short growing seasons, reflected in a broad range of LES strategies across tundra species (see Pierce et al, 2017, A global method for calculating plant CSR ecological strategies applied across biomes world-wide).

Perhaps it would be better here to focus on size-related traits instead such as low plant height?

"a high fraction given that trait variation is widely known to be deter- mined as well by other factors such as biotic or anthropogenic effects or disturbances"
Please avoid phrases such as widely known without references to back this up

"Our analyses highlight the dominance of the joint effect of climate and soil drivers to explain trait variation - a phenomenon previously little explored or appreciated."
I disagree. E.g. see Ordonez, GEB, 2009; Maire et al, GEB, 2015; Bjorkman et al, Nature, 2018; Dwyer & Laughlin, JVS, 2017 etc…

"On the opposite end of the gradient, sandy soils require adaptations to both water and nutrient limitation"

How does this interact with non-linear relationship with moisture e.g. saturation in peatland soils?

“We excluded observations that were not geo- referenced, because we could not attribute them to ecoregions.”
Did you also exclude data from experimental treatments (e.g. fertilisation), or from botanic gardens?

“These selection criteria serves as a quality control, because ecoregions with poor representation of species richness are excluded, as we can expect the regression to the mean to be stronger with more species data”
Did you test for this?

Reviewer #2 (Remarks to the Author):

Summary. Authors use a robust set of data to assess multiple plant functional traits important for capturing variation in plant trait distributions in response to climate and soil variability at global scales. This work extends understanding of global patterns of plant trait variability and provides insight of the environmental drivers important for understanding the variability. They use the Diaz et al 2016 global spectrum of plant form and function to test the drivers of trait variability, specifically, climate and soil gradients. The findings presented in this manuscript support the global spectrum of plant form and function and highlight the importance of the joint effect of climate and soil in explaining the distribution of size and economic traits, but also underline the importance soil plays in explaining variation in economic traits. I think that work presented here is important for trait-based ecology, but that the manuscript needs additional work. I have highlighted major and minor areas for improvement.
Major Comments:
Overall: This is an important project that identifies the environmental drivers of the global spectrum of plant form and function, but there are areas in the text that could use additional clarity and a shifted focus on how the work presented here extends the work done by Diaz et al 2016 and is not simply redoing it. I think that changing some of the terminology in the sections that focus on the global spectrum. For example, in the introduction “Orthogonal axes and trait cluster” section instead of framing the first sentence as whether or not the 17 traits hold for the previously identified 6 traits. I think this study extends understanding of the global spectrum by assessing the original traits and additional traits related to the two dimensions identified by the global spectrum as well as assesses their environmental drivers. Therefore, the authors which use the same or similar pool of trait data for the original 6 traits should expect the same orthogonal axes of trait clustering for those 6 traits, but the key difference that I think gets lost is that this manuscripts explores 11 new traits that also fall along this same global trait spectrum. The framing of the Diaz et al paper is used in multiple places throughout the manuscript and should be edited to shift to the focus of this paper. For example, in the section highlighted here, changing the first sentence to reflect the aims of this paper to something along the lines of “To identify the axes of variation explained by the 17 functional traits observed in this study, we use methods presented in Diaz et al 2016 and clustered trait-trait correlations (Figure 1a, S13), and further represent these relations based on their principal components (PCA, see Methods). We found additional support of size versus economics traits identified by Diaz and colleagues (10), where all but 3 of the new traits assessed fell along the two dimensions of the global

spectrum.” I understand that the work presented in this study builds upon the work done by Diaz et al, but it is using the framework outlined by Diaz et al to test how additional traits fall along these dimensions as well as how climate and soil variables may help explain the variation observed at global scales and I think this gets lost throughout the manuscript and much of that is related to how the work is explain in relation to the previous study.

Climate and soil: joint and independent effects. This section needs work. I understand that the authors would like to highlight the shift in importance of soil on economic traits, but the current text over emphasizes the importance of climate on size traits, when the results suggest that climate is important across all traits, but that soil becomes increasingly important for economic traits. In the current state it is also difficult to link which analysis are being referred to throughout the text and even in the figure/table descriptions. For example, in Figure 3, does both (a) and (b) refer to hierarchical partitioning? If so, it may be helpful to change the first sentence to reflect that “Hierarchical partitioning identifies climate and soil variables’ contribution to explain each trait (ecoregional median trait, blue=size, red=economics, yellow=other).” And change (b) to reduce redundancy and state what is present there. For example, “Percent variation explained by climate (purple), soil (peach), and joint (grey) assorted according to trait groups: size, economics, other.”

Discussion: This section is difficult to follow. It needs to be broken up into smaller sections with clear headings to help distill how the key findings relate to the literature and extend our understanding. In its current state, as one long paragraph, it is easy to lose track of the topic and miss the important links made. The main text uses subheaders that help the reader know what is coming and I think this section would really benefit from a similar structure. I also think this section is missing a few citation, especially with respect to the soils – formation, microbes, organic matter, nutrient cycling. Some of the work cited in the introduction can be revisited here, such as Chapin 1980, Vitousek et al 2004, Reich et al 2004 and others. I also think that since the soil characteristic presented in this study focuses on physical and chemical properties, other studies that highlight soil biological characteristics/processes that are not explored here, but also impact plant trait distributions and help to explain variation in economic and even some of the "other" traits. Adding a sentence or two about how soil biology may help explain some of the observed uncertainty.

Figures: Overall, figure descriptions need clarity and consistent terminology used throughout the text, especially for analyses used.

Minor Comments:

Main text:

Page 7: “Early plant biogeographers (12-14)…” move citation to the end of “function (12-14),…”

Page 12: Change “intuition” to “hypothesis”

Page 12: “We investigate the power of climate and soil variables…” change to support what you show in table 1 and fig 3 “We assess the joint and independent effects of climate and soil on trait variability.”

Page 12: “Overall, size traits are better explained…” Better explained by what? Help your reader by specifying where you are pulling these values from. Add “Table 1” to the parenthesis. And clarify in the text which analysis the r2 come from. For example, “Overall, size traits are better explained by climate and soil using ridge regressions? (Table 1; r2=0.55; maximum r2=0.78 for conduit density) than are economics traits (Table 1; r2=0.40; maximum r2=0.55 for Leaf N:P ratio).”

Page 12: Change “specific” to “observed” in the following sentence: “…which reflects strong interactions between specific climate and soil predictors (Figure 3b).”

Page 12: Change “…ubiquitous across traits,…” to “…observed across most traits,..” since climate alone explains 0% of the variation observed in vessel length.

Discussion:

Page 14: “In this study, we find that the global spectrum of plant form and function, divided into size

and economics traits by Dıaz and colleagues based on a 6-trait analysis (10), still holds for our extended database of 17 traits (Figure 1b).” Yes, this is true, but this is expected since this study largely uses the same dataset as Diaz et al 2016. I think it would be a stronger statement is to extend your findings to the reader by changing the focus. For example, “In this study, we identify 9 additional traits that support the global spectrum of plant form and function (10), seven traits that capture the whole plant size spectrum and seven traits that capture the leaf economic spectrum, with only three traits that not fall along these dimensions (Figure 1b).”
Methods:
Page 46: “For each of the 867 regions, we calculated the median of all species median trait values.” Maybe clarify, I think the authors are saying “For each of the 867 ecoregions, we calculated the median ecoregion aggregate trait value from the median trait values of all species identified in each region.” But they could be referring to the median at the species-level from traits measured at the individual-level.
Table 1: Add another header level that identifies the analysis used. For example, above the “Explained Variance by Soil and Climate [r2]” column add another column head above it that states “Ridge Regression Model” and above the “Soil, Climate, Joint” column add a header that states “Hierarchical Partitioning”
Supplemental Data.
Trait data table states that TRY is the source of trait data, however in the text of the main manuscript there is a statement in the Methods that states that the data include published literature “We extracted data on 17 plant functional traits from the TRY database (9) (Table S2, www.trydb.org, accession date July 2017, request nb.3282) including published literature (11, 45–88, 88–298).” How do the data represented in the published literature differ from those from the TRY database, or are those data included in the TRY database and represent a coupled climate/soil/ trait collection comparison? If the data from the published literature differ from the TRY database in their use in this manuscript, please state how. If not, and those data are part of the TRY database, it might be helpful to clarify this. Maybe just changing the word “including” to “representing” or “which include”.
Trait data table “(N/P) ratio/ 56” is repeated.

Reviewer #3 (Remarks to the Author):

Joswig et al. have analysed the variation of two main trait axes across soil and climate gradients globally, a first of its kind in terms of number of traits and coverage as gappiness in trait databases has prevented doing such global comparisons previously. One outstanding issue in this study is the lack of a more detailed explanation/exploration of the use of the gap-filling algorithm. Currently, there are no more than three lines of text describing the imputation method. While the use of imputing methods is conventional, how imputations are carried out has direct repercussions on the final structure of the data and therefore analyses. I think that explaining the following points is necessary for the reader to be fully aware of the impact of the imputing methods on the results:

Raw data and BHPMF thresholds and implementation
How was BHPMF implemented? In the study you say “The data were attributed to ecoregions (26) (Table S5, Figure extended data 1) and aggregated to species median values” does that mean that you calculated the imputed values using the whole dataset and “stop” BHPMF at the individual record level and then calculated species median at the ecoregion level, or something else?
What was the gappiness per traits and across the database? What is BHPMF threshold for gaps?

As in any database, TRY will have some errors. On the other hand, BHPMF reproduces extreme values very accurately. Because of this BHPMF is generally better at capturing the shape of the scatter of observed trait data, but also because of this it is better at reproducing errors in the data. What is the error rate in the TRY version used? What measures were taken to do QC of the data?

Prediction accuracy:
It is known that within global databases, imputation techniques may introduce inaccurate information in the case of traits that are both very plastic and highly influenced by local environmental conditions. Similarly, it is now known that for traits that are mainly determined by phylogeny, imputation methods may increase the tightness of their correlation with other traits. Were any steps taken to account for or describe BHPMF impact on prediction accuracy under these scenarios?

I am not too worried about traits which are well-known and well-represented in global databases, but more suspect of traits that are either less-well represented across the phylogeny and whose variation we know less about globally.
For plastic traits and traits with tight correlations, and for highly conserved traits, how much does the trait-trait correlation change comparing the original data vs imputed values?

Minor comments
Page 18: You mention “Secondly, economics traits show relatively more within-site variation than across-site variation in comparison to size traits (Figure S9), one reason being that economics traits are sensitive to light availability, which often varies strongly at the local scale (41)”
However, leaf traits measurements for most datasets are made using the plant traits handbook (i.e. Cornelissen et al or Perez-Harguindeguy) and are therefore focused on top canopy, fully developed leaves. So, while the statement above is generally true is probably not true for the data you used.

The differences in areas are a problem for the use of ecoregion. This should be controlled for. Also, a term for sampling intensity could be included to account for differences in how much an ecoregion is sampled

Is the explanatory power of latitude preserved after adding the climate and soil variables?

********************END********************

Author Rebuttal to Initial comments

Answer to reviewers

Reviewer expertise:

Reviewer #1: Plant traits

Reviewer #2: Biogeography of plant traits

Reviewer #3: Biogeography methods, gap-filling

Reviewers' comments:

Reviewer #1 (Remarks to the Author):

The manuscript 'Climatic and soil factors explain the two-dimensional spectrum of global plant trait variation' continues and brings together recent work to interrogate global plant trait variation.

Since the publication of Diaz and colleagues 2015 paper identifying two major axes of global trait space (usually simplified to plant size and plant economics), a range of authors have sought to test and apply the conclusions, particularly in order to better understand how global change drivers may restructure ecosystems around the globe.

This manuscript neatly addresses a key unknown within this field of endeavour: whether axes of trait variation correspond to environmental gradients at the global scale, either independently or jointly. This is the first instance I am aware of that brings together both multiple traits and multiple environmental drivers within a global framework. This is critical to better understand how changes to environmental variables, either among ecosystems or over time, drive changes in multivariate trait space – and by extension plant community composition, and ecosystem function.

Joswig and colleagues find that the two main axes of trait variation are influenced by different environmental drivers. Traits relating to plant size exhibit a clear latitudinal gradient, which is well explained by climatic factors such as energy and water limitation. In contrast, economic traits do not exhibit a latitudinal gradient, and are better explained by soil characteristics. Together, these interacting drivers of trait expression present a framework to bring together individual trait-environment relationships and provide insight into plant life-history strategies within and among ecosystems.

On the whole, I feel that this is a well written paper, based on a substantial global trait database, with a robust set of analyses to support findings. Nevertheless, I have a number of concerns that

primarily relate to the clarity and novelty of the findings, which at present limit the significance of these findings, particularly to those from other disciplines. My major concerns are set out below.

We would like to thank the reviewer for the thorough and thoughtful comments which have substantially strengthened the manuscript.

Rev1_1 | 1. Questions and hypotheses.

I did not find that the paper set out clearly enough what is was aiming to test. For example, the overall hypothesis, we hypothesize that global patterns of trait correlation should closely follow gradients of climate and soil properties, is neither immediately intuitive nor particularly testable, particularly to those unfamiliar with this field.

In contrast, the questions underpinning this hypothesis statement are clearer: (1) to what extent the major dimensions underpinning the global spectrum of plant form and function can be attributed to global gradients of climate and soil conditions, and (2) to what extent these factors can jointly or independently explain the global spectrum of form and function (though I feel this is just an extension of the first question).

In my view, the manuscript also tests a second question in testing the robustness of the observed relationships using multiple traits, across multiple ecosystems, and for woody and non-woody species.

We thank the reviewer for this assessment. We would like to emphasize that this paper is essentially a data-driven investigation and we are interested in letting the data speak - writing down too-narrow hypotheses post hoc would not accurately reflect our approach. This is why we would like to retain the general statement of our expectations, but add the specific questions that have guided the analysis. We also rephrased "hypothesize" to "expect" in order to emphasize the data-driven nature of our approach.

We agree that the question of the woody/non-woody species is important and should be mentioned from the beginning.

The corresponding section now reads (ll. 148 to 156):

> *"Combining the insights that the global spectrum of plant traits **reveals** two internally correlated, orthogonal groups, and that many plant traits are individually linked to environmental gradients, we **expect** that global patterns of trait correlation should closely follow gradients of climate and soil properties. **He**re we investigate to what extent the major dimensions underpinning the global spectrum of plant form and function can be attributed to global gradients of climate and soil conditions; and to what extent these factors can jointly or independently explain the global spectrum of form and function. **To address this general question we first need to determine whether the two trait axes persist in a larger dataset including additional traits, and whether the previously identified dominant subsets of woody and non-woody species react alike."***

In essence, this analysis therefore brings together two major areas of study within plant trait literature, in a way that is currently underplayed.

Rev1_1.1 | a. Testing the two axes of global trait space (synthesis). The two axes of trait space have been tested and challenged in a number of ways since 2015. Those that I have seen (there may be others) include:

- That relationships do not hold for different traits (trait-dependent)
- That relationships do not hold for different plant types (group-dependent)
- That relationships do not hold in different ecosystems (ecosystem-dependent)
- That relationships do not hold at different scales (scale-dependent)

The authors demonstrate that the two axes are retained when analysis is increased from 6 to 17 traits, when performed for woody and non-woody species, and when performed with different combinations of species and ecoregions. Together, these analyses could offer a strong defence of the two axes, in a way that brings together key criticisms and potentially synthesises a number of individual previous studies using this strong global dataset. While that potential exists within the findings presented (including many in the supplementary materials), the wider relevance and implications of these tests is not well articulated. In part this may stem from weak recognition of the body of work performed since 2015 (see point 2).

Thank you for this suggestion.

We now added to the introduction (ll. 120-130):

> *"Analysing six fundamental traits, Diaz and colleagues (Diaz et al. 2015) revealed that essential patterns of form and function across the plant kingdom can be captured by two main axes. The first reflects the size spectrum of whole plants and plant organs. The second axis corresponds to the "leaf economics spectrum" (LES) (Wright et al. 2004) emerging from the necessity for plants to balance leaf persistence against plant growth potential.* ***The concept of a global spectrum of plant form and function has since been investigated from various perspectives (Bruelheide et al. 2018, Thomas et al. 2020, Kong et al. 2019). It has been shown, for instance, that orthogonal axes of variation in size and economics traits emerge even in the extreme tundra biome (Thomas et al. 2020) , or at the scale of plant communities (Bruelheide et al. 2018). However, it remains unclear whether the two axes remain dominant for extended sets of traits, or when differentiating among growth forms. A particular knowledge gap is what environmental controls determine these two axes of plant form and function."***

We now revised the first paragraph in the discussion (lines 236-242):

> ***"This study shows that the proposed global spectrum of plant form and function fits well to a substantially extended trait space compared to the original study (Diaz et al. 2015), with seven traits that capture the whole plant size spectrum and seven traits***

that capture the leaf economic spectrum, and only three traits that do not fall along these dimensions (Figure 1b). One explanation could be that the varying fraction of woody and non-woody species would drive these patterns. However, we showed that these two main trait groups remain clearly identifiable when the analysis is conducted separately for woody and non-woody species (see Figures S3 to S5). "

Due to data restrictions, we could not perform analyses on the woody and non-woody groups at the same scale for ecoregions, and the number of ecoregions was cut to 86 and 84, respectively. We thus consider these analyses not to be as robust as our main analysis. Therefore we decided not to include wider implications. Yet, we do emphasize the general support our study provides for a universal trait spectrum more prominently now in the introduction and discussion.

Rev1_1.2 | b. Testing the utility of the two axes of global trait space, via relationship with environmental drivers (novel framework and analysis). If the two axes of traits variation are considered robust, a key question is therefore how this understanding may be applied; in the words of the authors, they have the potential to be "powerful predictors of plant community assembly and ecosystem functioning", particularly for global models of vegetation dynamics and land-climate feedbacks.

Indeed, there are many developments including plant traits and functional diversity into terrestrial biosphere models (Fyllas et al. 2014, Sakschewski et al. 2016, Gaillart et al. 2018, Langan, Higgins & Scheiter 2017). We have now commented on implications in a paragraph regarding how our analysis can be used to calibrate or validate these global models. (lines 342-343)

"Implications
Our analysis can serve as reference for model developments that increasingly consider plant functional traits as part of vegetation dynamics under climate change."

Rev1_1.3 | The authors note that there is a long history of study of the relationships between individual traits and environmental variables (p7). While not mentioned in the text, there has also been growing evidence that the two axes of trait variation are driven by different environmental variables, in some cases leading to unexpected results (see point 2 below). However, the authors are correct in noting that this is the first instance I am aware of that brings together both multiple traits and multiple environmental drivers within one framework.

This aspect of the study is well developed and relatively clearly articulated (in line with the question above), but would be strengthened by (1) a clearer hypothesis statement that specifically articulates a prediction, and (2) clearer articulation of why this question is of value – particularly to other disciplines.

We thank the reviewer for this comment.
Regarding (1) we refer the reviewer to our earlier response (Rev1_1.1).

Regarding (2), we dedicate a whole new paragraph in the discussion to articulate why the approach and question, which environmental variables drive multiple traits are of value, especially with respect to other studies in the future (see below and lines 342-373).

Discussion (lines 342-373):

"Implications

Our analysis can serve as reference for model developments that increasingly consider plant functional traits as part of vegetation dynamics under climate change.

Individual plants and their trait syndromes are considered to be viable only within specific environmental conditions (Kraft et al. 2015). Therefore trait-environment relationships should be scale-independent. However, different plant strategies can be successful under given environmental conditions, which in addition are often confounded by small scale variation. In analyses to date, trait-environment relationships become more apparent for aggregations higher than the community scale (Bruelheide et al. 2018), where most of the small-scale variation is averaged out. In addition the difference between potential and actual vegetation is hypothesized to explain some of this gap (Thomas et al. 2020). Dynamic global vegetation models (DGVMs) predict individual plant processes well, but fail to produce reliable forecasts with a changing environment (Franklin et al. 2020). Deciphering at which spatial and temporal scale, or conditions, actual vegetation is representative of potential vegetation may advance our understanding of community assembly and necessary model complexity.

Trait-environment correlations identified in our study should not be confounded with causality. Yet, the ubiquitous importance of climate variables for traits suggest trait shifts with climate change. Trait shifts are constrained by available trait combinations in addition to other constraints such as species dispersal. For example, our results indicate that plant size increases with temperature so long as sufficient water is available (Figure 4, S13 and S24, S23, S25), in line with the finding that species become larger and large species are more prevalent as temperatures increase in the tundra (Bjorkman et al. 2018).
Global change is also reflected by soil degradation. Changes in soil parameters can be considered to also correspond with trait shifts, especially for economics traits. Human-induced soil degradation has many facets: often fertile topsoil is lost, or toxic substances accumulate; rooting is impeded, and altered by artificial fertilizers, while soil formation takes millenia (Oldeman et al. 1991). The trait shifts may thus be similarly complex, and depend on the extent and type of soil degradation. For example, in areas of wind and water erosion, species that tolerate lower nutrient availability may be more successful, and this may be reflected in lower leaf nutrient contents (Figures 4, S34). The fertilization of nutrient-poor grasslands, e.g. resulting from agricultural run-off, may shift these areas from more conservative to more competitive species with higher leaf nutrient contents.

Plants as a whole need to balance both size and economics traits. To sustain human livelihood, it may be important to understand the local expression of trait shifts and their global consequences for biodiversity when viable trait combinations change. "

In detail we now explicitly refer to growing evidence that the two axes of trait variation are universal and driven by different environmental variables.

Introduction (lines 135 to 136):

"Over the last decades, examples have thus accumulated without an overall framework in which to place them ***(Maire et al. 2015, Bjorkman et al. 2018, Thomas et al. 2020).****"*

Discussion (lines 275 to 280):

"The climate and soil factors used in this analysis explain up to 78% of observed trait variation - a high fraction given that trait variation is widely known to be determined as well by other factors such as biotic or anthropogenic effects or disturbances.

Recent findings on how different trait groups vary with the environment indicate that size and economics traits vary differently (Thomas et al. 2020), affected by climate and soil (Bjorkman et al. 2018).*"*

Rev1_2 | 2. Acknowledgement of previous work.

My second major criticism is that the manuscript currently does not sufficiently acknowledge and build on recent work to interrogate plant trait variation in the last five years. At times this reads as if the study is being published as an immediate follow-up to the 2015 work, rather than following 5-6 years of intense scrutiny and further investigation by numerous teams. This is relatively easy to address, and would build a stronger case for a need for the 'overall framework' set out in this manuscript. To name a few examples,

- Bruelheide and colleagues (NEE, 2017 Global trait–environment relationships of plant communities) perform a global, plot-level analysis of trait–environment relationships, finding strong filtering of 17 functional traits but emphasising local filtering of traits
- Bjorkman and colleagues (Nature, Plant functional trait change across a warming tundra biome, 2018), examine biome-wide relationships between temperature, soil moisture and the same key traits as the Diaz (2015) study), empahsising the differential roles played by temperature and moisture on each trait
- Thomas and colleagues (NatComms, Global plant trait relationships extend to the climatic extremes of the tundra biome, 2020) perform a test of the two trait axes within tundra environments, finding that size traits but not economic traits are constrained by environmental conditions, "indicating that two of the major axes of global trait variation may be differentially selected by environmental conditions, and could thus respond differently to environmental change".
- A large number of other studies have tested trait axes at different scales, with different traits (e.g. root traits), in different ecosystems and sites, and with different functional groups.

I note this, not to take away from the novelty of findings, but to put them in context of the large body of work underway, that already points to the framework outlined here. I would disagree that "we lack general narratives describing these fundamental relationships at global scale" (for example, I would say warm and wet = big and tall; cold and/ or dry = small and hardy is a reasonably general narrative). However, I would agree that "examples have accumulated without an overall framework in which to place them". To me this is where the real strength of this study lies – an opportunity to place the emerging but disparate evidence of the findings articulated here into a robust and consistent framework.

We thank the reviewer for this comment, acknowledge recent work, and refer to our earlier responses in 1.a and 1.b (our responses Rev1_1.1, Rev1_1.2, Rev1_1.3). Additionally, we substitute the formulation of "general narratives" and introduce the recent findings on trait-environment relationships in the introduction (lines 132 to 142):

> *"There is ample evidence that large-scale variation of individual plant traits is related to environmental gradients. Early plant biogeographers suggested that climate and soils together shape plant form and function (Schimper 1903, Warming 1909, Raunkiaær 1934)* ***but could not propose a more precise theoretical framework describing these fundamental relationships. Over the last decades,*** *examples have* ***thus*** *accumulated without an overall framework in which to place them* ***(Maire et al. 2015, Bjorkman et al. 2018, Thomas et al. 2020)****. For instance, tree height depends on water availability (Olson et al. 2018, Moles et al. 2009) while leaf economics traits depend on soil properties, especially soil nutrient supply, as well as on climatic conditions reflected in precipitation (Ordonez et al. 2009, Maire et al. 2015, Simpson et al. 2016). Leaf size, leaf dark respiration rate, specific leaf area (SLA), leaf N and P concentration, seed size and wood density, all show broadscale correlations with climate or soil (Wright et al. 2017, Atkin et al. 2015, Ordonez et al. 2009, Asner et al. 2016, Moles et al. 2007). It has also been reported that many of these traits show latitudinal patterns (Wright et al. 2017, Atkin et al. 2015, Asner et al. 2016, Moles et al. 2007). Generalizing [...]"*

Rev1_3 | 3. Broader utility of findings.

Given the ambition of this analysis, I feel that it misses the opportunity to go further and be of greater interest and use to those working beyond plant ecology. The analysis provides quantitative relationships between PCA axes and latitude (which is interesting), but largely descriptive investigations of the relationships between PCA axes and environmental drivers, beyond their relative importance. However, if this work is to truly help to 'constrain parameters of global coupled climate- vegetation models ', I suspect this needs to go beyond latitude (which does not change) to quantify relationships between PCA axes and (interacting) environmental drivers.

Instead, the link with water and energy limitation is currently indirect: a relationship between trait PCA axes and latitude, and a relationship between latitude and energy / moisture, therefore a

relationship between trait PCA axes and energy / moisture. A powerful next step would be to test – and quantify – the relationship between trait PCA axes and energy / moisture.

As examples, the variation explained by environmental drivers is set out in the manuscript both for PCA axes (main findings) and for individual traits (table 1), and to some extent the direction in PCAs, but these are not quantified in the same way as for latitude. The relationship between PCA axes and latitude is set out (Figure 2), but with not for key environmental drivers (or combinations of environmental drivers).
Although this may beyond the scope of this analysis, one suggestion may be to develop the multi-panel figures used for single traits (S20-37), but applied to PCA axes, with additional information provided for relationships with key environmental variables.

I would invite the authors to consider how this further step could be investigated and communicated in this study, or at very least to set out how it could be developed further in future analyses.

We thank the reviewer for this comment and refer with respect to the implications of this study to answer to 1b (response Rev1_1.3*).*
We quantify the relationships between single traits and single environmental drivers in our supplementary material on single traits (Figures S22-S39; and for PCA S40-S42) and note that the RDA assesses the relationship between the PC axes (since this is the first step of an RDA, shown in figure 4a), and the single environmental variables. However, the multi-panel figures for single traits (Figures S22e-39e), provide estimates of linear trait-environment relationships from explained variance of linear models and correlation coefficients that indicate the direction and importance of environmental variables. We now also provide for the first three PCs multi-panel figures that point to soil physics (texture, gravel, water capacity, soil density) being of most importance for PC2 (Figure S41, but see below this answer the figures).

We have now furthermore added to the discussion (lines 262-264):

> *"**Future studies should quantify how individual stressors, e.g. radiative stress or water stress, relate to global patterns of trait variation**."*

We note (line 189):

> *"**The remaining PCs each account for less than 10% of variance (PC3=9.36%).**"*

We recognize that the latitudinal analysis is not very informative by itself, but we present it as a motivation for our main analysis which explores the relationships between PC axes and environmental drivers using RDA. We now furthermore conduct a hierarchical partitioning analysis to determine the extent to which latitude and climate or soil are redundant explanatory variables for variation in traits (see response Rev3_7.3).

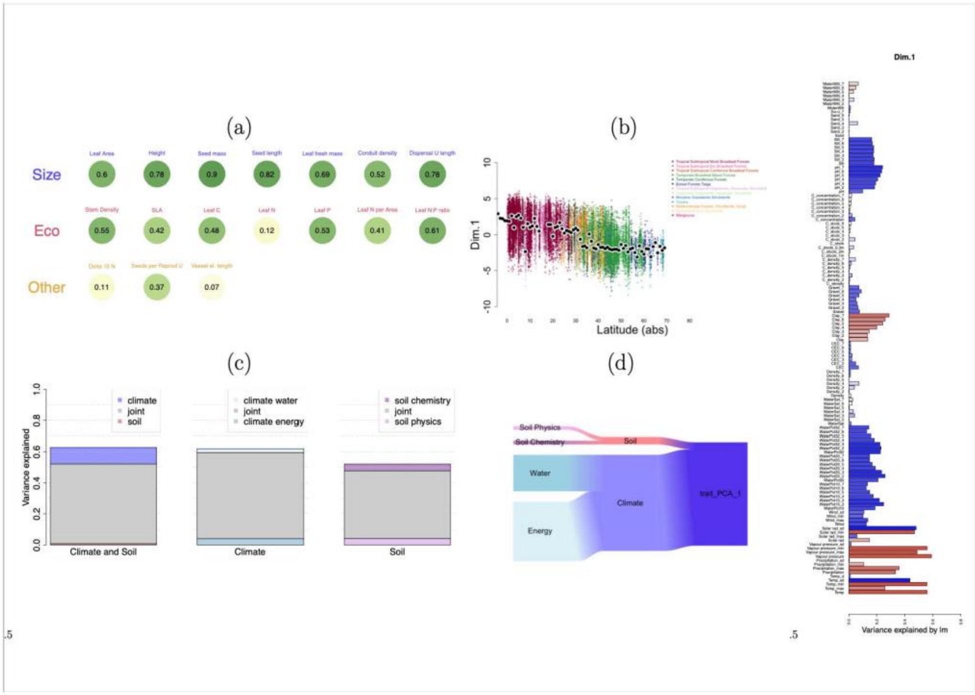
(a)
(b)
(c)
(d)
Size
Eco
Other
Leaf Area
Height
Seed mass
Seed length
Leaf fresh mass
Conduit density
Dispersal U length
0.6
0.78
0.9
0.82
0.69
0.52
0.78
Stem Density
SLA
Leaf C
Leaf N
Leaf P
Leaf N per Area
Leaf N:P ratio
0.55
0.42
0.48
0.12
0.53
0.41
0.61
Delta 15 N
Seeds per Reprod U
Vessel el. length
0.11
0.37
0.07
Dim.1
Latitude (abs)
Variance explained
climate
joint
soil
climate water
climate energy
soil chemistry
soil physics
Climate and Soil
Climate
Soil
Soil Physics
Soil Chemistry
Water
Energy
Climate
trait_PCA_1
Variance explained by lm

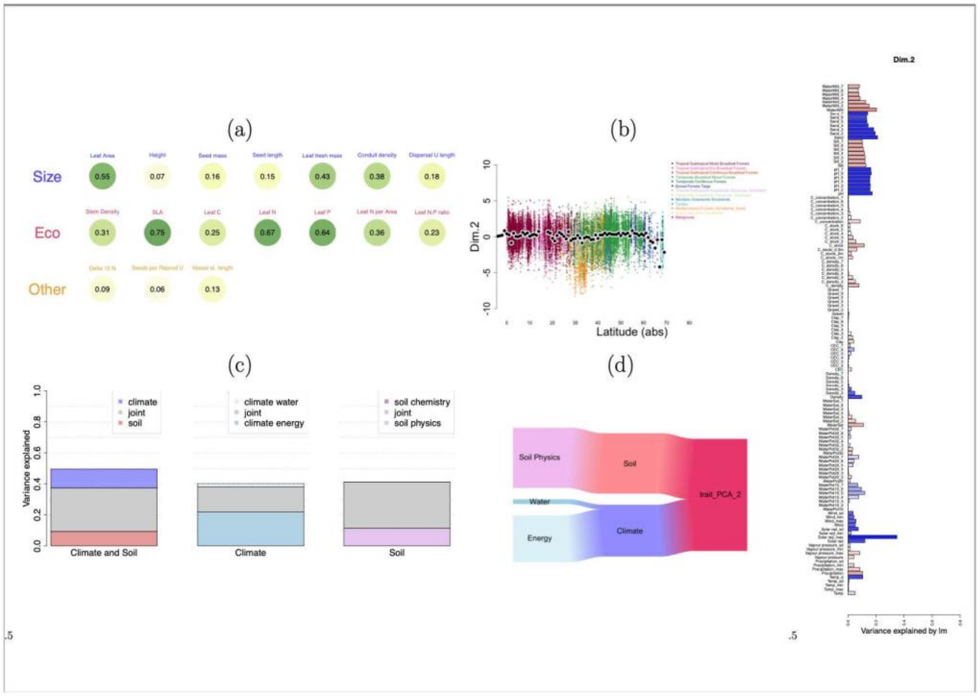
(a)
(b)
(c)
(d)
Size
Eco
Other
Leaf Area
Height
Seed mass
Seed length
Leaf fresh mass
Conduit density
Dispersal U length
0.55
0.07
0.16
0.15
0.43
0.38
0.18
Stem Density
SLA
Leaf C
Leaf N
Leaf P
Leaf N per Area
Leaf N:P ratio
0.31
0.75
0.25
0.67
0.64
0.36
0.23
Delta 15 N
Seeds per Reprod U
Vessel el. length
0.09
0.06
0.13
Dim.2
Latitude (abs)
Variance explained
climate
joint
soil
climate water
climate energy
soil chemistry
soil physics
Climate and Soil
Climate
Soil
Soil Physics
Water
Energy
Climate
trait_PCA_2
Variance explained by lm

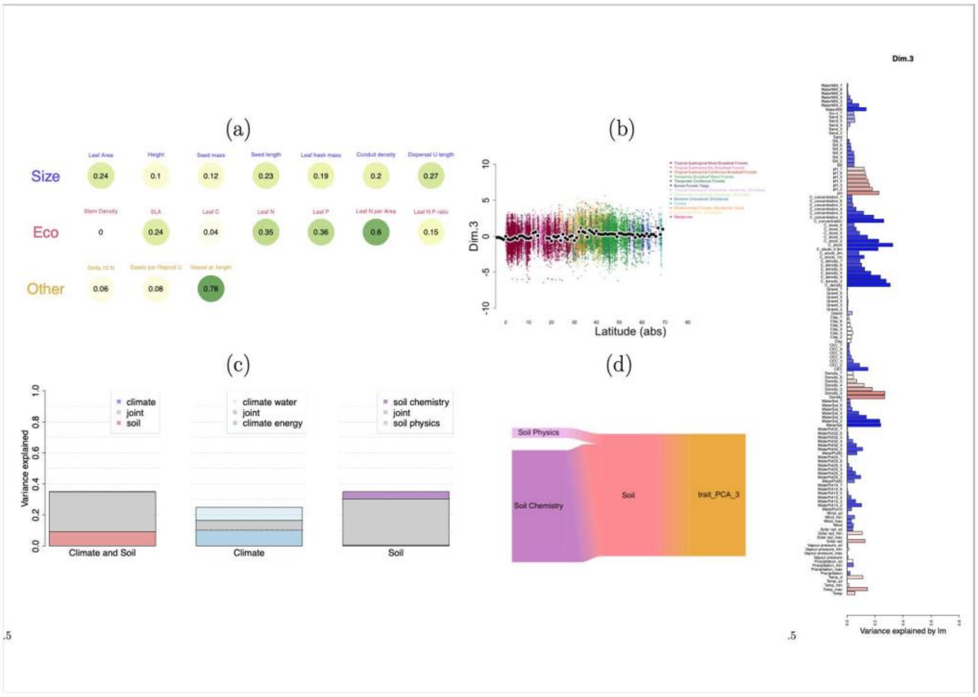

Rev1_4 | 4. Bias towards temperate / forest ecosystems

The authors set out concerns that using grid cells to scale results would be globally uneven in comparison to ecoregions, and a global sampling bias towards Europe. However, I am concerned that the filtering process applied to ecoregions to ensure sufficient data quality also results in a similar sampling bias, since ecoregions with sufficient data and more likely to come from well sampled regions. This is visible in Figure 4 and extended data fig 1.

Given very environmental conditions between ecoregions, the authors should do more to demonstrate that the relationships found here between traits environmental drivers are also consistent within ecoregions, and do not result from strong patterns once data has been aggregated at the ecoregion level or within the two-three most common ecoregion types (which appear to be Tropical Subtropical Moist Broadleaf Forests and Temperate Broadleaf Mixed Forests). A short metadata table to accompany the long list of ecoregions would also be helpful here.

Thank you for raising this concern. We would argue, however, that the bias in sample abundances is accounted for by the selection criterion (>20 species and 1% of expected species

richness, see also answer Rev1_6.22). We commented on this aspect in the discussion (see below). The interpretation we have for the well-explained variation is that actual trait values on the level of ecoregion aggregation are close enough to the potential trait values under given environmental conditions. In other words, we have a theoretical basis for preferring ecoregion-level aggregation and we also think that it allows us to account for some biases in data availability.

Additionally, in order to test for the possibility of a sampling bias, we sampled the minimum number of species, according to the selection criterion. This resulted in oversampled ecoregions to reduce species number, while other ecoregions retained the same samples. We repeated this analysis three times with similar results as in Figure 3b (supplementary analysis 6.2).

We reflect on the issue of scale in the discussion (lines 344 to 354):

> ***"Individual plants and their trait syndromes are considered to be viable only within specific environmental conditions (Kraft et al. 2015). Therefore trait-environment relationships should be scale-independent. However, different plant strategies can be successful under given environmental conditions, which in addition are often confounded by small scale variation. In analyses to date, trait-environment relationships become more apparent for aggregations higher than the community scale (Bruelheide et al. 2018), where most of the small-scale variation is averaged out. In addition the difference between potential and actual vegetation is hypothesized to explain some of this gap (Thomas et al. 2020). Dynamic global vegetation models (DGVMs) predict individual plant processes well, but fail to produce reliable forecasts with a changing environment (Franklin et al. 2020). Deciphering at which spatial and temporal scale, or conditions, actual vegetation is representative of potential vegetation may advance our understanding of community assembly and necessary model complexity."***

Rev1_5 | 5. General clarity

Given the scope and impact of the paper, the discussion would warrant significant shortening to **focus on the key messages**. At times the text becomes somewhat **repetitive** of similar overarching themes, and also focuses on certain detailed points which I am not sure are essential (e.g. aeolian and fluviatile sorting processes of glacial moraines).
Instead, the discussion could more succinctly could draw out the specific implications of findings. For example, might it be true to infer that climate warming might be expected to predominantly affect size-related traits, while anthropogenic alterations to soil conditions may affect economic traits? This is hinted at in the text, but this hypothesis would provide a great number of avenues for future work.

Thank you for motivating us to add more clarity to the discussion. We hope to have increased clarity by

1. *A new paragraph focusing on the implications with connections to recent literature (see Rev1_1.3)*
2. *Cutting out repetitive sentences, e.g. on aeolian and fluviatile sorting processes*
3. *Adding subheaders (Latitudinal gradient, Ecoregion scale, Environmentally explained trait variation, Joint effect, Environmental variables explain plant size, Environmental variables explain plant economics, Explained variation size versus economics, Implications and Conclusions)*

Specific comments:

Rev1_6.1 | "…environmental controls contributing to these two main axes. There is ample evidence that…"
Suggest you start a new paragraph here for clarity
Changed accordingly

Rev1_6.2 | "…climate and soils together shape plant form and function…"
Would it be possible to add a very short example here for the wider audience?
Thank you, but we are not quite sure what is meant here. We give the following examples (lines 136 to 142):

> *" For instance, tree height depends on water availability (Olson et al. 2018, Moles et al. 2009) while leaf economics traits depend on soil properties, especially soil nutrient supply, as well as on climatic conditions reflected in precipitation (Ordoñez et al. 2009, Maire et al. 2015, Simpson et al. 2016). Leaf size, leaf dark respiration rate, SLA, leaf N and P concentration, seed size and wood density all show broad-scale correlations with climate or soil (Wright et al. 2017, Atkin et al. 2015,Ordonez et al. 2009, Asner et al. 2016, Moles et al. 2007). **It has also been reported that** many of these traits show latitudinal patterns (Wright et al. 2017, Atkin et al. 2015, Asner et al. 2016, Moles et al. 2007)."*

Rev1_6.3 | "we still lack general narratives describing these fundamental relationships at global scale"
I would disagree with this. I think we have general narratives, but lack a strong framework to explain these narratives.
We thank the reviewer and refer to the comments above.

Rev1_6.4 | "Rather, examples have accumulated without an overall framework in which to place them"
I agree with this, think this is the important point - we know the overall trends, but don't necessarily have a functional framework within to place these trends.
In other words, we have broad correlations at the global scale, and we have specific relationships for a multiple individual traits, but we do not necessarily have a framework to bring those two elements together within global multivariate trait space.
We thank the reviewer for this clear statement and hope that our revised submission can contribute to filling this gap.

Rev1_6.5 | “Many of these traits show latitudinal patterns”
True, though it is not the latitudinal patterns we are interested in, per se, but rather the causal relationships behind these latitudinal patterns.
Exactly, they are only a first estimate. We indicate this (lines 191 to 193):

> *“As a first investigation of broad scale gradients among size and economics traits, we analyze latitudinal gradients of the first (PC1) and second (PC2) principal components.”*

Further we point to the bundled nature of environmental variables within latitude (lines 199 to 203):

> *“Latitudinal gradients are known to be strongly related to climate, due to the distribution of solar energy and general atmospheric circulation patterns. Therefore, we hypothesize that those climate (and soil) aspects that covary with latitude consistently determine size traits, while they have little effect on economics traits, which are more strongly affected by latitude-independent soil (and climate) effects* ***(Figure S10)****. ”*

Rev1_6.6 | “Combining the insights that the global spectrum of plant traits comprises two internally correlated, orthogonal groups, and that many plant traits are individually linked to environmental gradients, we hypothesize that global patterns of trait correlation should closely follow gradients of climate and soil properties”
This is very confusing hypothesis sentence. I am not quite sure what the hypothesis that “patterns of trait correlation follow climate and soil gradients” means, nor immediately how you say it has been met / falsified.
We appreciate this concern. Please see the response to point 1a (our response Rev1_1.1).

Rev1_6.7 | “Therefore, we hypothesize that those climate (and soil) aspects that covary with latitude consistently determine size traits, while they have little effect on economics traits, which are more strongly affected by latitude-independent soil (and climate) effects.”
Ref Thomas et al, 2020 for a real world example of this.
Done (see above).

Rev1_6.8 | “We investigate the power of climate and soil variables for predicting each of these traits”.
Given the potential importance of microclimate / local soil effects for economic traits identified in this study (but also Bruelheide et al, 2017), is there a risk that you cannot draw meaningful relationships between economic traits and soil variables at this relatively macro scale.
This is an important point. We would argue that the results of our analysis do indicate that the importance of soil characteristics for trait distributions can also be detected at this larger (macro) scale. In the discussion however, we include this consideration when reflecting on the question of why soil is a weaker predictor (lines 326 to 341):

> “ The lower fraction of explained variance for economics traits could have several causes: firstly, data on soil factors which are likely to be very important, such as soil nitrogen and phosphorus availability (Maire et al. 2015, Simpson et al. 2016), are not yet available at a global scale. Secondly, economics traits show relatively more within-site variation than across-site variation in comparison to size traits (Figure S9), *likely because* economics **traits vary within one plant, e.g. leaf N per area and SLA vary with age and light**

availability (Niinemets et al. 2016). Thirdly, soil heterogeneity within ecoregions -- both abiotic and biotic -- may weaken the relationship between economics traits and environmental variables *(Butler et al. 2017, Freschet et al. 2011, Bruelheide et al. 2018).* Reasons for small scale soil variation are e.g. topography, soil age and thus fertility (Yemefack et al. 2005), **but also abundance of microbial communities and mycorrhiza that interact with climate, pH, soil properties and also plant traits (deVries et al. 2012). Trait-environment relationships due to smaller scale variation require well resolved soil data. However, we note that soil physics and chemistry explains a large portion of variance along the trait PC axis three (which itself explains slightly less than 10% of variance in the PCA (9.36%); see Figures S1, S2, S42). We expect that with improved soil data sets and a higher resolution, the joint control of climate and soil on trait variation will most likely appear even stronger and more evenly distributed between the two groups of driver variables."**

Rev1_6.9 | "Overall, size traits are better explained (r2=0.55; maximum r2=0.78 for conduit density) than are economics traits (r2=0.40; maximum r2=0.55 for Leaf N:P ratio)."

Neat

Thank you!

Rev1_6.10 | "The independent climate effects are ubiquitous across traits, but size traits tend to be better explained by the independent climate effects than are economics traits. In contrast, independent soil predictors are relevant for all economics traits - but not size traits (apart from a small contribution to leaf area)."

Ref Bjorkman et al, 2018 for a real-world example of this across climate gradients using plot-level data

Thank you, we now integrate Bjørkman et al. 2018 in the discussion of this result (lines 278 to 280).

"Recent findings on how different trait groups vary with the environment indicate size and economics traits to vary differently (Thomas et al. 2020, Nature communications), and jointly with climate and soil (Bjørkman et al. 2018, Nature)."

Rev1_6.11 | "These two main trait groups remain clearly identifiable when the analysis is conducted separately for woody and non-woody species"

This is a very nice sensitivity analysis, though not clear to the general reader why this is needed or why it is relevant.

Thank you. Because these two groups do dominate different areas in the first published global distribution analysis (Diaz et al. 2015), and according to comments from other reviewers, we do think it is important to include this subset analysis. However, as mentioned elsewhere in this response, we do not wish to place too much emphasis on it since the power we have for this subset is much lower than for the full dataset.

Rev1_6.12 | Discussion in general – suggest you think about how you break this up into paragraphs. Some key points are somewhat lost in the mass of text.

Thank you for this suggestion. Done accordingly. See answer 5 (Rev1_5).

Rev1_6.13 | "Additional traits may add relevant axes of trait variation"

"I think this comment is too broad - after stating that the two axes hold for 17 traits, it doesn't follow to simply say that more traits may add more information. Rather, I think the value you can add, based on the analysis conducted in this manuscript, is to draw out what particular drivers of variation may not be picked up by the traits being tested…and therefore what traits you may predict we would wish to target to better develop in trait databases."

We thank the reviewer for this insight. Our analysis does show lower explained variance for the first two axes than for the 6 traits in the Diaz 2015 study. It is not clear if further axes are meaningful; e.g. for leaf reflectance, higher-dimensional axes may be more important (Mereiles et al. 2020). This is also why hierarchical partitioning in combination with trait-trait correlations (Figure 1b), or trait connections (Flores-Moreno et al. 2018, GEB), adds important information. Still, we added a potentially important set of traits that are neglected so far, and these do generally fall well within the division between size and economics. We also point out three of these traits which do not cluster into one of these two categories.

We furthermore note that (lines 243 to 248):

> *"However, we cannot discard the possibility that additional traits may add relevant axes of trait variation. For example, our study does not include carbon fixation rates\cite{Shipley2005FunctionalModels}* ***or fire adaptation traits (He et al. 2016), nor does it include any root traits - representing an essential gap to be filled at the global scale (Bergmann et al. 2017). The respective data are too scarce to yet be integrated with global data sets. If such data were available they would have the potential to fundamentally change our perception of global plant form and function, and their relation to ecosystem functioning."***

Rev1_6.14 | "Variation in size traits, represented by PC1 in Figure 1b, shows a clear latitudinal gradient."

While this is a neat finding, I am not sure it is the most important one in the context of this analysis. I am reminded of a comment on another manuscript, which used trait data to identify changes at treelines. The reviewer considered it to be a smart approach, but noted that it was not particularly novel to conclude that there were no trees north of the treeline. In other words, the broad trait trends with latitude identified here are interesting, and the approach (particularly looking across multiple size traits) is very good, but the conclusion – variation by latitude – is not especially groundbreaking.

We agree that this in itself is not a groundbreaking finding, but it is necessary for generating the hypothesis that size and economics traits are differently explained. See response 3 (Rev1_3).

Rev1_6.15 | In many places this analysis is pitched in terms of improving bioclimatic / specie envelope models, which care less about latitude and more about trait-environment relationships. Given that you have specifically looked at the relationship between the PCA axes and climatic variables (e.g. energy+moisture), I think this could provide a more novel outcome, which would have obvious implications for modelling impacts of climate change, for example.

Thank you for this suggestion. A more detailed answer can be found in response 3 (Rev1_3).

Rev1_6.16 | "In addition to a decrease at high latitudes above 60 (absolute) where, however, species data become increasingly limited"
I notice this analysis does not include more recent (open access) high-latitude trait data from Bjorkman et al, 2019 – see Tundra Trait Team: A database of plant traits spanning the tundra biome. That dataset roughly doubled the previously available TRY trait data above 60 degrees, so would be of value here.
Thank you, we included the most complete data set at the time when the analysis was conducted. We are aware of the Bjorkman data set and agree it could address this specific limitation, but adding them to latitudinal gradients does not show substantial alterations, we thus do not expect any changes in the main conclusions of the study. Consequently, we have explained this in our text (methods), but also discuss this aspect accordingly to acknowledge this (discussion).

We now add to the Discussion (lines 261 to 262):

> ***"Additional data sets may shed more light on specific conditions, e.g. Bjorkman et al. (Bjorkman et al. 2018). "***

We added to the Methods (lines 886 to 887):

> ***"The largest possible data set was retrieved at the time when study was conducted; including 172 traits of 652,957 individuals (Table S4). "***
>
> *The general pattern are confirmed by independent data, which became available more recently (and are therefore not included in the overall analyses, see Figures S17 and below).*

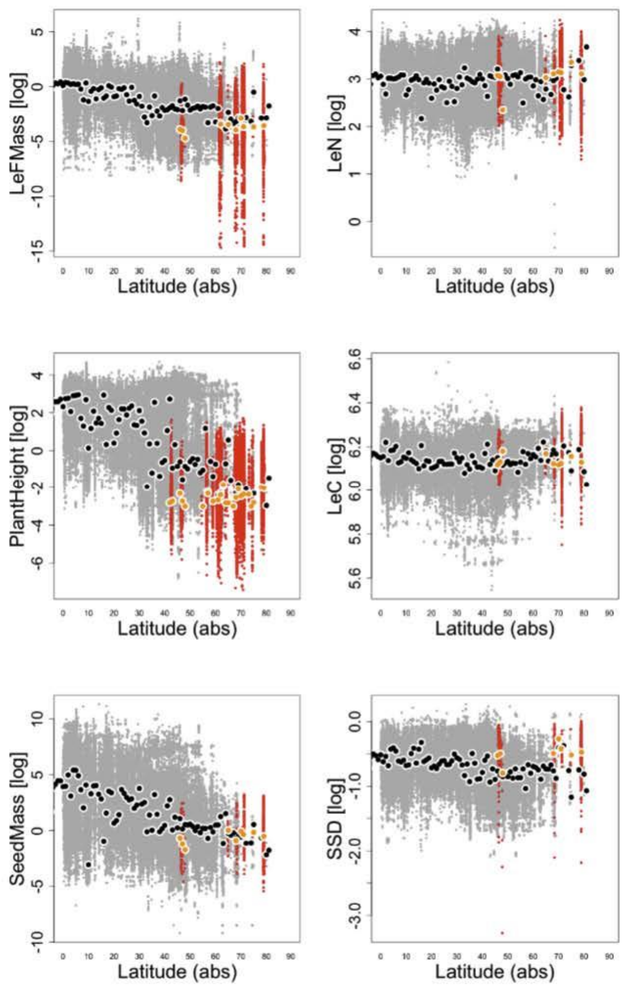

Figure S17: Latitudinal trait gradients of data as used in this study (gray), added with data from the tundra (*27*) (red). Points (black, orange) refer to binned trait values (absolute latitudinal degree) of data used in this study and the tundra data respectively (*27*).

Rev1_6.17 | "At high latitudes, cold winters and short growing seasons demand more conservative nutrient-use strategies (like evergreen leaves) and protection against frost damage" I would not fully agree with this, and regardless, this would not be relevant to size traits (which this discussion is focusing on). While tundra species are generally more conservative compared to the global mean, there is still widespread variation. For example, some tundra species (e.g. alpine forbs) also have highly opportunistic strategies to make use of short growing seasons, reflected in a broad range of LES strategies across tundra species (see Pierce et al, 2017, A global method for calculating plant CSR ecological strategies applied across biomes world-wide).

Perhaps it would be better here to focus on size-related traits instead such as low plant height?

Thank you, we now write (lines 258 to 261):

> *" At high latitudes, cold winters and short growing seasons **constrain plant height (Thomas et al. 2020) and require on average more conservative nutrient-use strategies (like evergreen leaves) and protection against frost damage than the global mean, despite the high functional diversity in economics traits observed at these latitudes (Thomas et al. 2020)."***

Rev1_6.18 | "a high fraction given that trait variation is widely known to be deter- mined as well by other factors such as biotic or anthropogenic effects or disturbances"
Please avoid phrases such as widely known without references to back this up

Agreed. We added (lines 275 to 280):

> *"The climate and soil factors used in this analysis explain up to 78% of observed trait variation - a high fraction given that trait variation is widely known to be determined as well by other factors such as biotic **interactions (e.g. soil biota)** and anthropogenic effects or disturbances,and local effects such as those of micro-climate (**Franklin et al. 2020, Legay et al. 2014,** Grime1974, Bruelheide et al. 2018). **Recent findings on how different trait groups vary with the environment indicate that size and economics traits vary differently (Thomas et al. 2020), and in particular respond differently to climate and soil (Bjorkman et al. 2018)."***

Rev1_6.19 | "Our analyses highlight the dominance of the joint effect of climate and soil drivers to explain trait variation - a phenomenon previously little explored or appreciated."
I disagree. E.g. see Ordonez, GEB, 2009; Maire et al, GEB, 2015; Bjorkman et al, Nature, 2018; Dwyer & Laughlin, JVS, 2017 etc…
We now adjusted this statement to (lines 281 to 283):

> *"Our analyses reveal a dominant joint effect of climate and soil drivers on trait variation **- - as already suggested by a number of earlier studies (Ordoñez et al. 2009, Maire et al. 2015, Bjorkman et al. 2018), but not yet quantified globally. "***

Rev1_6.20 | "On the opposite end of the gradient, sandy soils require adaptations to both water and nutrient limitation"
How does this interact with non-linear relationship with moisture e.g. saturation in peatland soils?

Our analysis looks at ecoregions, for which the peatland soils are too coarse. Acidic, nutrient poor and oxygen deprived peatlands can be found under a wide range of climatic conditions, but are not the dominant ecoregion type. Therefore we do not derive any "peatland" ecoregion.

In the discussion we write (line 266)

> *"Our main analysis is based on median trait values of plant species per ecoregion."*

We now suggest with respect to peatlands smaller-scale soil data (lines 331 to 336). These may also unveil non-linear trait-environment relationships.

"Reasons for small scale soil variation are e.g. topography, soil age and thus fertility (Yemefack et al. 2005), but also abundance of microbial communities and mycorrhiza that interact with climate, pH, soil properties and also plant traits (de Vries et al.). Trait-environment relationships due to smaller scale variation require well resolved soil data."

Further, we comment on the aspect of scaling with respect to non-linear relationships (lines 344 to 351).

"Individual plants and their trait syndromes are considered to be viable only within specific environmental conditions (Kraft et al. 2015). Therefore trait-environment relationships should be scale-independent. However, different plant strategies can be successful under given environmental conditions, which in addition are often confounded by small scale variation. In analyses to date, trait-environment relationships become more apparent for aggregations higher than the community scale (Bruelheide et al. 2018), where most of the small-scale variation is averaged out. In addition the difference between potential and actual vegetation is hypothesized to explain some of this gap (Thomas et al. 2020)."

Rev1_6.21 |"We excluded observations that were not geo- referenced, because we could not attribute them to ecoregions."
Did you also exclude data from experimental treatments (e.g. fertilisation), or from botanic gardens?
Yes, according to the TRY regulations. We clarified this in the text (lines 868 to 870):

*"We excluded observations that were not geo-referenced, because we could not attribute them to ecoregions, **according to TRY regulations, also data from experimental treatments (e.g. fertilisation), or from botanic gardens we excluded.**"*

Rev1_6.22 |"These selection criteria serves as a quality control, because ecoregions with poor representation of species richness are excluded, as we can expect the regression to the mean to be stronger with more species data"
Did you test for this?

We did perform preliminary tests with different selection criteria (e.g. number of species and inclusion or exclusion of 1% of species richness estimate by Kier). Indeed, lower numbers of species per ecoregion showed weaker explained variation, while stricter rules reduced the number of ecoregions.

We now write in the Methods section (lines 956 to 959):

"Preliminary tests with different selection criteria (e.g. number of species and inclusion or exclusion of 1% of species richness estimate by Kier showed lower numbers of species per ecoregion result in weaker explained variation, while stricter rules reduced the number of ecoregions."

Reviewer #2 (Remarks to the Author):

Summary. Authors use a robust set of data to assess multiple plant functional traits important for capturing variation in plant trait distributions in response to climate and soil variability at global scales. This work extends understanding of global patterns of plant trait variability and provides insight of the environmental drivers important for understanding the variability. They use the Diaz et al 2016 global spectrum of plant form and function to test the drivers of trait variability, specifically, climate and soil gradients. The findings presented in this manuscript support the global spectrum of plant form and function and highlight the importance of the joint effect of climate and soil in explaining the distribution of size and economic traits, but also underline the importance soil plays in explaining variation in economic traits. I think that work presented here is important for trait-based ecology, but that the manuscript needs additional work. I have highlighted
major and minor areas for improvement.
We thank the reviewer for the constructive criticism of our study.

Major Comments:
Rev2_1 | Overall: This is an important project that identifies the environmental drivers of the global spectrum of plant form and function, but there are areas in the text that could use additional clarity and a shifted focus on how the work presented here extends the work done by Diaz et al 2016 and is not simply redoing it. I think that changing some of the terminology in the sections that focus on the global spectrum. For example, in the introduction “Orthogonal axes and trait cluster” section instead of framing the first sentence as whether or not the 17 traits hold for the previously identified 6 traits. I think this study extends understanding of the global spectrum by assessing the original traits and additional traits related to the two dimensions identified by the global spectrum as well as assesses their environmental drivers. Therefore, the authors which use the same or similar pool of trait data for the original 6 traits should expect the same orthogonal axes of trait
clustering for those 6 traits, but the key difference that I think gets lost is that this manuscripts explores 11 new traits that also fall along this same global trait spectrum. The framing of the Diaz et al paper is used in multiple places throughout the manuscript and should be edited to shift to the focus of this paper. For example, in the section highlighted here, changing the first sentence to reflect the aims of this paper to something along the lines of “To identify the axes of variation explained by the 17 functional traits observed in this study, we use methods presented in Diaz et al 2016 and clustered trait-trait correlations (Figure 1a, S13), and further represent these relations based on their principal components (PCA, see Methods). We found additional support of size versus economics traits identified by Diaz and colleagues (10), where all but 3 of the new traits assessed fell along the two dimensions of the global spectrum.” I understand that the work presented in this study builds upon the work done by Diaz et al, but it is using the framework outlined by Diaz et al to test how additional traits fall along these dimensions as well as how climate and soil variables may help explain the variation observed at global scales and I think this gets lost throughout the manuscript and much of that is related to how the work is explain in relation to the previous study.

Thank you for this comment. We now bring forward the aspect of testing additional traits and novelty of our approach more generally.
Introduction (lines 124 to 130):

> ***"The concept of a global spectrum of plant form and function has since been investigated from various perspectives (Bruelheide et al. 2018, Thomas et al. 2020, Kong et al. 2019). It has been shown, for instance, that orthogonal axes of variation in size and economics traits emerge even in the extreme tundra biome (Thomas et al. 2020) , or at the scale of plant communities (Bruelheide et al. 2018). However, it remains unclear whether the two axes remain dominant for extended sets of traits, or when differentiating among growth forms. A particular knowledge gap is what environmental controls determine these two axes of plant form and function."***

Discussion (lines 236-242):

> ***"This study shows that the proposed global spectrum of plant form and function fits well to a substantially extended trait space compared to the original study (Diaz et al. 2015), with seven traits that capture the whole plant size spectrum and seven traits that capture the leaf economic spectrum, and only three traits that do not fall along these dimensions (Figure 1b). One explanation could be that the varying fraction of woody and non-woody species would drive these patterns. However, we showed that these two main trait groups remain clearly identifiable when the analysis is conducted separately for woody and non-woody species (see Figures S3 to S5). "***

(lines 281 to 283):

> *"Our analyses reveal a dominant joint effect of climate and soil drivers on trait variation - -* ***as already suggested by a number of earlier studies (Ordoñez et al. 2009, Maire et al. 2015, Bjorkman et al. 2018), but not yet quantified globally. "***

Rev2_2 | Climate and soil: joint and independent effects. This section needs work. I understand that the authors would like to highlight the shift in importance of soil on economic traits, but the current text over emphasizes the importance of climate on size traits, when the results suggest that climate is important across all traits, but that soil becomes increasingly important for economic traits.

In the current state it is also difficult to link which analysis are being referred to throughout the text and even in the figure/table descriptions. For example, in Figure 3, does both (a) and (b) refer to hierarchical partitioning? If so, it may be helpful to change the first sentence to reflect that "Hierarchical partitioning identifies climate and soil variables' contribution to explain each trait (ecoregional median trait, blue=size, red=economics, yellow=other)." And change (b) to reduce redundancy and state what is present there. For example, "Percent variation explained by climate (purple), soil (peach), and joint (grey) assorted according to trait groups: size, economics, other."

We thank the reviewer for this comment and have added the text with the method used (lines 208 to 215):

> *"We assess the joint and independent effects of climate and soil on trait variability (**ridge regression, RR**,Table 1), Figure 3). Overall, size traits are better explained (**RR**; r2=0.55;*

*maximum r2=0.78 for conduit density; Table 1) than are economics traits (**RR**; r2=0.40; maximum r2=0.55 for Leaf N:P ratio; Table 1). We find a substantial joint effect of climate and soil variables -- in every case larger than either unique effect -- which reflects strong interactions between specific climate and soil predictors (**RR with hierarchical partitioning (HP)**, Figure 3b). However, we also observe independent effects of climate and soil (**RR with HP**; Figure 3, Table 1).”*

We revised figure captions accordingly (see answer Rev2_4).

The paragraph “Climate and soil: joint and independent effects” only presents the results from the ridge regression analysis with hierarchical partitioning. In the discussion, we focus on climate and soil separately, especially in the discussion (see below) and hope to have sufficiently described the importance of both, especially given requests to reduce the length.

on climate (lines 286 to 307).

*“The orthogonality of the two main dimensions of plant trait variation suggests that different aspects of climate and soil variables are relevant to explain plant trait patterns at the global scale (Figures S11 to S14, **S10). While latitude-related variables, mainly climate - explain size traits, variables that share less explanatory power with latitude - mainly soil - explain economics traits (Table 1, Figure S10).** The RDA presented in Figure 4 (Figure S13) provides some insight on the nature of these climate - soil interactions. The first RDA axis, which describes variation in size traits, resembles a latitudinal gradient. On one extreme end, ample water supply from high and frequent precipitation, abundant water vapour, and constant rates of high solar radiation meet the fundamental requirements of plant physiology: water, sunlight and warm temperatures. Additionally, these conditions promote weathering of soil minerals; but also microbial activity, contributing to fast turnover rates of organic matter supporting nutrient provisioning **(Slessarev et al. 2016, Blume et al. 2016)**; in brief, they represent conditions, which allow plants to grow fast and tall in the race for light. Large vessels supporting large leaves promote high rates of water transport and thus growth, which is only possible because of the small risk of embolism under these benign water conditions (Zanne et al. 2018). The high carbon gains can be invested in large fruits and seeds (seed mass, seed length, dispersal unit length). Further along this gradient, the above mentioned plant requirements become limited: water supply and temperatures are reduced and slow metabolic rates above- and belowground. In ecoregions of the boreal and desert biomes, conduit diameter is constrained by the risk of cavitation during freeze-thaw cycles (Zanne et al. 2018) and water scarcity, amplified by little water holding capacity of gravel-rich soils. Our analysis thus indicates that size traits appear to be related to a latitudinal gradient of climatic favorability for plant growth determined by water and light availability.“*

on soil (lines 308 to 327):

*“Important correlates of water and nutrient availability are associated with the second RDA axis, describing variation in economics traits. Traits associated with an acquisitive strategy are related to indicators of soil fertility, most importantly silt and organic matter concentration as well as pH **(Maire et al. 2015, Blume et al. 2016). Soil pH** is intermediate between the two axes, as might be expected given that pH both reflects broadscale climate*

*variation (especially aridity Slessarev et al. 2016), and a variety of processes related to nutrient availability **and soil microbial communities (Maire et al. 2015, Fierer et al. 2006, Sinsabaugh et al. 2012, deVries et al. 2012)**. Silt forms the substrate of our most fertile soils as its structure is able to retain water against gravitation (unlike sand), but renders it accessible to plants under drought conditions (**Zech et al. 2014**, Blume et al. 2016) (unlike clay). The high fertility is associated with a high concentration of organic matter, which has a high cation exchange capacity especially under high pH (Slessarev et al. 2016). On the opposite end of the gradient, sandy soils require adaptations to both water and nutrient limitation. The trait configuration at the conservative end of the economics traits (low SLA, high tissue density, high organ longevity) represents an adaptation to both (Wright et al. 2004, **Chapin 1980**). Various processes exist that lead to variation in the soil characteristics underlying the second RDA axis independent of latitude (**Maire et al. 2015**) -- for example, sandstone as a geological substrate giving rise to sandy soils exists from the tropics to the arctic (Blume et al. 2016, Zech et al. 2014). However, different climate variables related to solar radiation, temperature and precipitation, which influence long-and short-term soil development processes **directly and indirectly via soil biology** (**Rosenberg et al. 2012**, Blume et al. 2016, Zech et al. 2014), are related to this axis. Variation in economic traits is most likely the evolutionary response to exploiting this partly climate-independent edaphic niche axis."*

Further, on the explained variability and soil see Rev1_6.8

Rev2_3 | Discussion: This section is difficult to follow. It needs to be broken up into smaller sections with clear headings to help distill how the key findings relate to the literature and extend our understanding. In its current state, as one long paragraph, it is easy to lose track of the topic and miss the important links made. The main text uses subheaders that help the reader know what is coming and I think this section would really benefit from a similar structure. I also think this section is missing a few citation, especially with respect to the soils – formation, microbes, organic matter, nutrient cycling. Some of the work cited in the introduction can be revisited here, such as Chapin 1980, Vitousek et al 2004, Reich et al 2004 and others. I also think that since the soil characteristic presented in this study focuses on physical and chemical properties, other studies that highlight soil biological characteristics/processes that are not explored here, but also impact plant
trait distributions and help to explain variation in economic and even some of the "other" traits. Adding a sentence or two about how soil biology may help explain some of the observed uncertainty.

Thank you. We have divided the discussion into paragraphs and added subheadings.

Moreover we revise (and add) some references:
lines 293-296:

"On one extreme end, ample water supply from high and frequent precipitation, abundant water vapour, and constant rates of high solar radiation meet the fundamental requirements of plant physiology: water, sunlight and warm temperatures. Additionally, these conditions promote weathering of soil minerals; but also microbial activity, contributing to fast turnover

*rates of organic matter supporting nutrient provisioning (Slessarev et al. 2016, **Blume et al. 2016, Rosenberg et al. 2012)**; in brief, they represent conditions, which allow plants to grow fast and tall in the race for light. "*

lines 306-324 (as in Rev2_2*):*

*"Important correlates of water and nutrient availability are associated with the second RDA axis, describing variation in economics traits. Traits associated with an acquisitive strategy are related to indicators of soil fertility, most importantly silt and organic matter concentration as well as pH **(Maire et al. 2015, Blume et al. 2016). Soil pH** is intermediate between the two axes, as might be expected given that pH both reflects broadscale climate variation (especially aridity Slessarev et al. 2016), and a variety of processes related to nutrient availability **and soil microbial communities (Maire et al. 2015, Fierer et al. 2006, Sinsabaugh et al. 2012, deVries et al. 2012)**. Silt forms the substrate of our most fertile soils as its structure is able to retain water against gravitation (unlike sand), but renders it accessible to plants under drought conditions (**Zech et al. 2014**, Blume et al. 2016) (unlike clay). The high fertility is associated with a high concentration of organic matter, which has a high cation exchange capacity especially under high pH (Slessarev et al. 2016). On the opposite end of the gradient, sandy soils require adaptations to both water and nutrient limitation. The trait configuration at the conservative end of the economics traits (low SLA, high tissue density, high organ longevity) represents an adaptation to both (Wright et al. 2004, **Chapin 1980**). Various processes exist that lead to variation in the soil characteristics underlying the second RDA axis independent of latitude (**Maire et al. 2015**) -- for example, sandstone as a geological substrate giving rise to sandy soils exists from the tropics to the arctic (Blume et al. 2016, Zech et al. 2014). However, different climate variables related to solar radiation, temperature and precipitation, which influence long-and short-term soil development processes **directly and indirectly via soil biology** (**Rosenberg et al. 2012**, Blume et al. 2016, Zech et al. 2014), are related to this axis. Variation in economic traits is most likely the evolutionary response to exploiting this partly climate-independent edaphic niche axis."*

We also discuss the importance of soil biology shaping soil heterogeneity here (lines 326 to 341):

*" The lower fraction of explained variance for economics traits could have several causes: firstly, [...]. Secondly, [...]. **Thirdly, soil heterogeneity within ecoregions -- both abiotic and biotic -- may weaken the relationship between economics traits and environmental variables** (Butler et al. 2017, Freschet et al. 2011, Bruelheide et al. 2018). Reasons for small scale soil variation are e.g. topography, soil age and thus fertility (Yemefack et al. 2005), **but also abundance of microbial communities and mycorrhiza that interact with climate, pH, soil properties and also plant traits (deVries et al. 2012). Trait-environment relationships due to smaller scale variation require well resolved soil data. However, we note that soil physics and chemistry explains a large portion of variance along the trait PC axis three (which itself explains slightly less than 10% of variance in the PCA (9.36%); see Figures S1, S2, S42). We expect that with improved soil data sets and a higher resolution, the joint control of climate and soil on trait variation will most likely appear even stronger and more evenly distributed between the two groups of driver variables."***

Rev2_4 | Figures: Overall, figure descriptions need clarity and consistent terminology used throughout the text, especially for analyses used.
Thank you. We changed the following figure captions:

> *Figure 1:"**Previously identified global axes of variation in size and economics traits hold for an extended trait set. The set of 17 investigated traits can be primarily divided into size and eco- nomics traits, which load differently onto the two PC axes describing their global distribution.**"*
>
> *Figure 2:"**Size traits, not economics traits vary with latitude:** The first principal component (PC1) of the PCA on 17 plant traits **shows a clear latitudinal gradient while PC2 does not** (n=36,197, species per ecoregion median)."*
>
> *Figure 3:"**Climate and soil variables explain up to 78% of variance in size and economics traits. Hierarchical partitioning identifies the contribution of climate and soil variables to explain each trait (ecoregional median trait, blue=size, red=economics, yellow=other).**"*
>
> *Figure 4: "Redundancy analysis (RDA) of traits **reveals the relationships of climate and soil factors associated with trait distributions** (n=220, ecoregion median, only top soil layer variables included; variance explained: RDA1=63%, RDA2=18%). "*

Minor Comments:
Main text:
Rev2_5.1 | Page 7: "Early plant biogeographers (12-14)…" move citation to the end of "function (12-14),…"
Thank you, done accordingly.

Rev2_5.2 | Page 12: Change "intuition" to "hypothesis"
Thank you, done accordingly.

Rev2_5.3 | Page 12: "We investigate the power of climate and soil variables…" change to support what you show in table 1 and fig 3 "We assess the joint and independent effects of climate and soil on trait variability."
Thank you, done accordingly.

Rev2_5.4 | Page 12: "Overall, size traits are better explained…" Better explained by what? Help your reader by specifying where you are pulling these values from. Add "Table 1" to the parenthesis. And clarify in the text which analysis the r2 come from. For example, "Overall, size traits are better explained by climate and soil using ridge regressions? (Table 1; r2=0.55; maximum r2=0.78 for conduit density) than are economics traits (Table 1; r2=0.40; maximum r2=0.55 for Leaf N:P ratio)."

Thank you, changed accordingly.

Rev2_5.5 | Page 12: Change "specific" to "observed" in the following sentence: "…which reflects strong interactions between specific climate and soil predictors (Figure 3b)."

We indeed speak here about specific climate and soil variables, which interact. Therefore we would prefer to keep the wording as is.

Rev2_5.6 | Page 12: Change "…ubiquitous across traits,…" to "…observed across most traits,.." since climate alone explains 0% of the variation observed in vessel length.
Thank you, changed accordingly.

Discussion:
Rev2_5.7 | Page 14: "In this study, we find that the global spectrum of plant form and function, divided into size and economics traits by Dıaz and colleagues based on a 6-trait analysis (10), still holds for our extended database of 17 traits (Figure 1b)." Yes, this is true, but this is expected since this study largely uses the same dataset as Diaz et al 2016. I think it would be a stronger statement is to extend your findings to the reader by changing the focus. For example, "In this study, we identify 9 additional traits that support the global spectrum of plant form and function (10), seven traits that capture the whole plant size spectrum and seven traits that capture the leaf economic spectrum, with only three traits that not fall along these dimensions (Figure 1b)."
Thank you very much for this suggestion. We have changed the text accordingly.

Methods:
Rev2_5.8 | Page 46: "For each of the 867 regions, we calculated the median of all species median trait values." Maybe clarify, I think the authors are saying "For each of the 867 ecoregions, we calculated the median ecoregion aggregate trait value from the median trait values of all species identified in each region." But they could be referring to the median at the species-level from traits measured at the individual-level.

Thank you. We have changed the text accordingly.

Rev2_5.9 | Table 1: Add another header level that identifies the analysis used. For example, above the "Explained Variance by Soil and Climate [r2]" column add another column head above it that states "Ridge Regression Model" and above the "Soil, Climate, Joint" column add a header that states "Hierarchical Partitioning"
Thank you, this indeed clarifies it better. Changed accordingly.

Supplemental Data.
Rev2_5.10 | Trait data table states that TRY is the source of trait data, however in the text of the main manuscript there is a statement in the Methods that states that the data include published literature "We extracted data on 17 plant functional traits from the TRY database (9) (Table S2, www.trydb.org, accession date July 2017, request nb.3282) including published literature (11,

45–88, 88–298).” How do the data represented in the published literature differ from those from the TRY database, or are those data included in the TRY database and represent a coupled climate/soil/ trait collection comparison? If the data from the published literature differ from the TRY database in their use in this manuscript, please state how. If not, and those data are part of the TRY database, it might be helpful to clarify this. Maybe just changing the word “including” to “representing” or “which include”.
The data used for this study were accessed via TRY, but are derived from the mentioned studies. Data entering TRY undergoes a cleaning and harmonization process. Thank you for your suggested change, which we integrated.

Rev2_5.11 | Trait data table “(N/P) ratio/ 56” is repeated.
Thank you, erased.

Reviewer #3 (Remarks to the Author):

Rev3_1 | Joswig et al. have analysed the variation of two main trait axes across soil and climate gradients globally, a first of its kind in terms of number of traits and coverage as gappiness in trait databases has prevented doing such global comparisons previously. One outstanding issue in this study is the **lack of a more detailed explanation/exploration of the use of the gap-filling algorithm**. Currently, there are no more than three lines of text describing the imputation method. While the use of imputing methods is conventional, how imputations are carried out has direct repercussions on the final structure of the data and therefore analyses. I think that explaining the following points is necessary for the reader to be fully aware of the impact of the imputing methods on the results:

We appreciate this consideration and have added more information on the imputation to the methods section.
lines 878 to 905

> *“**Hierarchical Probabilistic Matrix Factorization***
>
> ***Description***
> ***BHPMF decomposes or factorizes probabilistically a matrix (probabilistic matrix factorization, PMF (301)) using information contained within different hierarchical levels (here: taxonomy) within a Bayesian framework (8). The underlying premise of BHPMF is to gap-fill (or more accurately, to predict) traits of an individual plant using trait-trait correlations as well as intra- and interspecific trait variability. (8, 302). Using a Gibbs sampler (a Markov Chain Monte Carlo algorithm), BHPMF also provides a prediction confidence in the form of standard deviations (SD) which is a per-value estimate of uncertainty in trait predictions (8).*** BHPMF can fill gaps if there is at least one value per row (species) and column (trait).

Implementation
The largest possible data set was retrieved at the time when study was conducted; including 15x traits of 600,000 individuals (Table Sx). *For data preparation before BHPMF, all individual-level trait data were firstly log transformed and secondly normalized via zlog transformation ($z=x-\bar{x}sd$). Log transformation was chosen to achieve a closer to normal distribution of values per trait (Kattge et al. 2011). This transformation is considered necessary, because a given difference for small trait values (absolute value) is likely to be physiologically more relevant than the same difference (absolute value) for large trait values. BHPMF internally splits the datasets randomly into a training dataset (80%), a test dataset (10%) and a validation dataset (10%).*
The training dataset is used during training of latent vectors, while the test data is tested against in order to improve the latent vectors, and finally the validation dataset serves as the basis for calculation of the RMSE and stopping the optimization of latent vectors within BHPMF (8). The validation dataset ensures ongoing amelioration of the model performance during the training process, and stops the process after 5 consecutive iterations with stable RMSE. The test dataset is used only on the lowest taxonomic level (individuals x traits). The R package BHPMF was run with a maximum of 1000 iterations, whereas the first 200 were discarded during the "burn-in" phase, as predictions of these iterations are likely to be influenced by the initialization of BHPMF rather than being part of the probability density distribution to be sampled by BHPMF. In order to avoid autocorrelation, only every 20th iteration was used to calculate the resulting trait values. The mean of these predictions result in the final trait values used as the output."

We would furthermore like to mention that we are currently preparing for publication a sensitivity analysis to evaluate the impact of BHPMF prediction patterns on taxonomic clustering and trait-trait correlation patterns (Joswig et al. in prep.). The results of this publication in preparation indicate that trait-trait relationships are robust under BHPMF prediction.

Additionally, we elaborate more on the data set that was used to impute missing entries (lines 855 to 860):

"We extracted data on 17 plant functional traits from ***a gap-filled version of TRY database*** *(Kattge et al. 2020) (Table \ref{tab_TRYtrait}, www.try-db.org, accession date July 2017, request nb.3282) which including published literature (11, 55–98, 98–308). [...] Prior to this, missing data were imputed using a Bayesian Hierarchical Probabilistic Matrix Factorization (BHPMF) algorithm (Shan et al. 2012, Schrodt et al. 2015) for an extended data set, derived from TRY (Tables S4). This was done to be able to include the maximum number of species in our analyses. Then the 17 traits were selected from the best represented."*

Rev3_2 | Raw data and BHPMF thresholds and implementation
How was BHPMF implemented? In the study you say "The data were attributed to ecoregions (26) (Table S5, Figure extended data 1) and aggregated to species median values" does that

mean that you calculated the imputed values using the whole dataset and "stop" BHPMF at the individual record level and then calculated species median at the ecoregion level, or something else?

Yes, we calculated the imputed values using the whole dataset and "stop" BHPMF at the individual record level. BHPMF calculates the imputations from 1000 Gibbs sampler (MonteCarloChain) imputations by taking the mean of every 20th imputation of these 1000 "versions", after the first 200 are removed. Yes, we then calculated species median at the ecoregion level.

We clarified this in the Methods section (lines 865 to 868):

> ***"The imputed values were calculated using the whole dataset at the individual record level. BHPMF calculates the imputations from 1000 Gibbs sampler (MonteCarloChain) imputations by taking the mean of every 20th imputation of these 1000 "versions", after the first 200 are removed. Then the species median was calculated at the ecoregion level. "***

What was the gappiness per traits and across the database?

This is shown in table S2. We used all available data for gap-filling and then selected only those traits for which data had the most complete global distribution for further analysis. Thus we expect that our gap-filled data are representative of the content of the TRY database.

Table S2: Trait information, including original trait name and ID as used in the TRY data set (try-db.org), abbreviation as used in this study, trait units and original number of observations in data.

Trait names	Trait Group	Trait name/ TRY TraitID	Unit	nb of observations
Seed length	Size	Seed length/ 27	mm	136
Dispersal U length	Size	Dispersal unit length/ 237	cm	26
Seed mass	Size	Seed dry mass/ 26	mg	6847
Height	Size	Plant height / 18	m	44971
Leaf fresh mass	Size	Leaf fresh mass/ 163	mg	21246
Leaf Area	Size	Leaf area/ 1	mm2	72686
Conduit density	Size	Stem conduit density (vessels and tracheids)/ 169	mm-2	153
Stem Density	Eco	Stem dry mass per stem fresh volume (stem specific density, SSD, wood density)/ 4	mg mm-3	12776
Leaf C	Eco	Leaf carbon (C) content per leaf dry mass/ 13	mg g-1	15510
Leaf N:P ratio	Eco	(N/P) ratio/ 56 (N/P) ratio/ 56	g g-1	6088
Leaf P	Eco	Leaf phosphorus (P) content per leaf dry mass/ 15	mg g-1	14655
Leaf N per Area	Eco	Leaf nitrogen (N) content per leaf area / 50	g m-2	15108
SLA	Eco	Leaf area per leaf dry mass (specific leaf are, SLA)/ 11	mm2 mg-1	71724
Leaf N	Eco	Leaf nitrogen (N) content per leaf dry mass/ 14	mg g-1	34719
Vessel el. length	Other	Wood vessel element length/ 282	m	0
Delta 15 N	Other	Leaf nitrogen (N) isotope signature (delta 15N)/ 78		8806
Seeds per Reprod U	Other	Seed number per reproduction unit/ 138		0

What is BHPMF threshold for gaps?

BHPMF can fill gaps if there is at least one value per row (species) and column (trait). The influence of starting information is investigated by Joswig et al. (in prep). To be on the safe side we used all available starting information, as mentioned in the previous response.

We added to the methods section (lines 885):

> *"**BHPMF can fill gaps if there is at least one value per row (species) and column (trait).**"*

Rev3_3 | As in any database, TRY will have some errors. On the other hand, BHPMF reproduces extreme values very accurately. Because of this BHPMF is generally better at capturing the shape of the scatter of observed trait data, but also because of this it is better at reproducing errors in the data. What is the error rate in the TRY version used? What measures were taken to do QC of the data?
*It is difficult to assess the error rate. However, we now added to the **methods** section (lines 857 to 858):*

> *"Quality control was conducted according to the published protocol of TRY (Kattge et al. 2011, Kattge et al. 2020). Traits with z- score >4 were excluded and those with z-score >3 were checked for plausibility."*

Rev3_4 | Prediction accuracy:
It is known that within global databases, imputation techniques may introduce inaccurate information in the case of traits that are both very plastic and highly influenced by local environmental conditions. Similarly, it is now known that for traits that are mainly determined by phylogeny, imputation methods may increase the tightness of their correlation with other traits. Were any steps taken to account for or describe BHPMF impact on prediction accuracy under these scenarios?
We indeed expect BHPMF to smooth some existing variation and to tighten taxonomic clustering. In our sensitivity analysis (Joswig et al., in prep.) the clustering is mainly increased for species and genera, less so for families and clades where more information per group is available. Bjorkman et al. 2018 find species, more than intraspecific variability to define trait shifts. We do not attempt to address intraspecific variability in this study, and focus on trait-trait relationships rather than taxonomic clustering of traits. Trait-trait relationships are more robust to prediction by BHPMF, as indicated above.

Rev3_5 | I am not too worried about traits which are well-known and well-represented in global databases, but more suspect of traits that are either less-well represented across the phylogeny and whose variation we know less about globally.
The 17 traits selected for this analysis are the 17 best represented quantitative traits in the TRY database. This was the reason for not using the full TRY Database for this analysis, but only for the initial gap filling, as described above.

Rev3_6 | For plastic traits and traits with tight correlations, and for highly conserved traits, how much does the trait-trait correlation change compared to the original data vs imputed values?
Good point, we now state in the methods (lines 906 to 908):

> *"Compared to the original data, the imputed values show a "significant similarity" in terms of trait-trait correlation, according to the Procrustes test provided in Diaz et al. 2015."*

Minor comments

Rev3_7.1 | Page 18: You mention "Secondly, economics traits show relatively more within-site variation than across-site variation in comparison to size traits (Figure S9), one reason being that economics traits are sensitive to light availability, which often varies strongly at the local scale (41)"
However, leaf traits measurements for most datasets are made using the plant traits handbook (i.e. Cornelissen et al or Perez-Harguindeguy) and are therefore focused on top canopy, fully developed leaves. So, while the statement above is generally true is probably not true for the data you used.

It is not clear if, and the extent to which the protocol is always followed (for example top canopy leaves may not always be accessible). We now add to the single reason another one (age dependency) which should apply to the TRY data either way, since this - especially for evergreens - cannot be controlled for (lines 328 to 331).

> *"Secondly, economics traits show relatively more within-site variation than across-site variation in comparison to size traits (Figure S9), **likely because economics traits vary within one plant, e.g. leaf N per area and SLA vary with age and light availability (Niinemets et al. 2016).**"*

Rev3_7.2 | The differences in areas are a problem for the use of ecoregion. This should be controlled for. Also, a term for sampling intensity could be included to account for differences in how much an ecoregion is sampled
The areas of the ecoregions define which species become aggregated, since not all pixels within an ecoregion have samples. Thus usually only a small number of pixels is aggregated per ecoregion. We have checked for bias due to aggregation and our test indicates that different sampling intensities do not influence the outcome of our analysis (Figure S18). The sampling intensity is shown in the extended data figure 1.

Rev3_7.3 | Is the explanatory power of latitude preserved after adding the climate and soil variables?
Thank you for this interesting question, which links the latitudinal approach to the single variable analysis on ecoregion scale. The results from our additional analysis point to the same direction as our key findings. We looked at the additional explained variation by latitude compared to climate and soil variables.

Most variation explained is joint between the latitude and climate or soil, and both joint effects are similarly high. Latitude does not add much explained variation to climate (median 0.7%), while it adds a bit more to soil (median 3.1%). This is in line with the observation of stronger relationships between latitude and climate, so that most information encapsulated in latitudes are already present in climate variables. On average 11.5 and 10.5% of the variation is explained uniquely by

climate and soil variables, respectively. We draw from this result that latitudinal information may be still too coarse for capturing the same patterns as at lower levels for both climate and soil.

Our results underline the importance of a latitude-related environmental effect (climate and soil alike) for size traits, by the great share of explained variation by both predictors.
Our results point once more to the importance of a latitude-independent effect of soil for economics traits. The independent effect of soil to explain economics traits is substantially greater than for explaining size traits (2.8% for size versus 19.8 for economics).

We added to the ***results*** *section (lines 199 to 203):*

> *"Latitudinal gradients are known to be strongly related to climate, due to the distribution of solar energy and general atmospheric circulation patterns. Therefore, we hypothesize that those climate (and soil) aspects that covary with latitude consistently determine size traits, while they have little effect on economics traits, which are more strongly affected by latitude-independent soil (and climate) effects* ***(Figure S10).*** *"*

We added to the ***discussion*** *(lines 284 to 289):*

> *"The orthogonality of the two main dimensions of plant trait variation suggests that different aspects of climate and soil variables are relevant to explain plant trait patterns at the global scale (Figures S11 to S13****, S10****).* ***While latitude-related variables - mainly climate - explain size traits, variables that share less explanatory power with latitude - mainly soil - explain economics traits (Table S1, Figure S10).*** *The RDA presented in Figure 4 (Figure S13) provides some insight on the nature of these climate - soil interactions."*

We added to the ***Supplementary material*** *figures showing complete results of hierarchical partitioning of ridge regression. See*

- *Table S1 and Figure S10*

Table S1: Independent and joint effect of latitude and climate or soil derived from ridge regression and hierarchical partitioning. Median for all traits and split into size or economics traits. Analysis based on ridge regression and hierarchical partitioning.

**Independent latitude**	**Joint**	**Independent climate**	**Independent soil**
Total			
0.06	25.89	12.01	
5.12	27.74		9.62
Size			
0.04	42.06	9.18	
9.07	32.32		4.13
Economics			
2.62	9.71	13.47	
1.02	12.73		15.43

- *Figures S10a and b.*

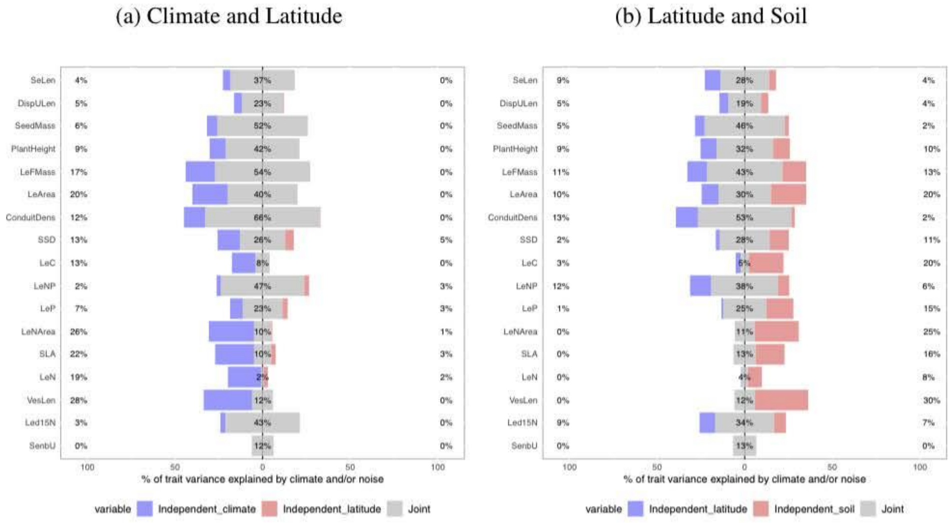

Methods in the Supplementary (lines 1098 to 1101 page S14):

> *"The analysis was conducted in the same way as the original analysis (see Methods), only replacing one of the variable types with latitude (median, max and min ecoregion aggregation). The comparison of latitude and ecoregions demanded an aggregation to ecoregions, also for latitude. "*

********************END********************

Our flexible approach during the COVID-19 pandemic

If you need more time at any stage of the peer-review process, please do let us know. While our systems will continue to remind you of the original timelines, we aim to be as flexible as possible during the current pandemic.

This email has been sent through the Springer Nature Tracking System NY-610A-NPG&MTS

Confidentiality Statement:

This e-mail is confidential and subject to copyright. Any unauthorised use or disclosure of its contents is prohibited. If you have received this email in error please notify our Manuscript Tracking Tracking System Helpdesk team at http://platformsupport.nature.com .

Details of the confidentiality and pre-publicity policy may be found here http://www.nature.com/authors/policies/confidentiality.html

Privacy Policy | Update Profile

DISCLAIMER: This e-mail is confidential and should not be used by anyone who is not the original intended recipient. If you have received this e-mail in error please inform the sender and delete it from your mailbox or any other storage mechanism. Springer Nature America, Inc. does not accept liability for any statements made which are clearly the sender's own and not expressly made on behalf of Springer Nature America, Inc. or one of their agents.

Please note that neither Springer Nature America, Inc. or any of its agents accept any responsibility for viruses that may be contained in this e-mail or its attachments and it is your responsibility to scan the e-mail and attachments (if any).

Decision Letter, first revision:

25th August 2021

Dear Dr. Joswig,

Thank you for submitting your revised manuscript "Climatic and soil factors explain the two-dimensional spectrum of global plant trait variation" (NATECOLEVOL-210112692A). It has now been seen again by the original reviewers and their comments are below. The reviewers find that the paper has improved in revision, and therefore we'll be happy in principle to publish it in Nature Ecology & Evolution, pending minor revisions to satisfy the reviewers' final requests and to comply with our editorial and formatting guidelines.

If the current version of your manuscript is in a PDF format, please email us a copy of the file in an editable format (Microsoft Word or LaTex)-- we can not proceed with PDFs at this stage.

We are now performing detailed checks on your paper and will send you a checklist detailing our editorial and formatting requirements in about a week. Please do not upload the final materials and make any revisions until you receive this additional information from us.

Thank you again for your interest in Nature Ecology & Evolution. Please do not hesitate to contact me if you have any questions.

[REDACTED]

Reviewer #1 (Remarks to the Author):

Thank you for the careful and thoughtful review. I am satisfied that you have addressed my previous concerns, and I believe that the manuscript now reads more clearly, with greater potential impact on the field.

I have no further major comments, but have included a small number of minor suggestions below that may add additional clarity.

L149-156: This new phrasing is helpful, and I agree with your approach. For slight additional clarity I might suggest you break up this into two sentences e.g.

Combining the insights that the global spectrum of plant traits reveals two internally correlated, orthogonal groups, and that many plant traits are individually linked to environmental gradients. We would therefore expect that global patterns of trait correlation should closely follow gradients of climate and soil properties.

L156-158: Given the rephrased 'hypothesis' (expectation) statement, I am no longer sure these sub-questions are needed. Although previously I highlighted them as a good example of a clear hypothesis statement, given the new approach they slightly weaken the previous statement. It is also not

immediately clear to the reader why you would need to determine those two questions in order to answer your overarching question. Instead, the reason for answering them comes through clearly enough later on in the text - to test a larger subset of traits (169-172), and to test whether relationships are different for woody vs non-woody species (189-191. I thought this was particularly clear!). I would therefore suggest you drop lines 156-158.

L223-224: I think I understand what you are getting at here, but this sentence could be overlooked as not particularly meaningful. Would it be helpful to add something like "..., as opposed to trait syndromes being defined by single environmental variables in isolation'

L264: Bjorkman rather than Bjørkman

L268-276: This paragraph is valid and important, but feels slightly out of place here. Is there scope to move to the methods or after line 168?

L328-329: This line does not make sense out of context. I suggest it needs qualifying in the context of the study e.g. "...by the environmental variables considered in this study".

L333: 'likely because economics traits vary within one plant' - so do some size traits (e.g. leaf area, leaf mass, seed mass). Suggest you add 'vary more than size traits' or something to that effect.

L359-360: This line is unclear, please could you rephrase. (e.g. Yet, the ubiquitous importance of climate variables for explaining current differences in trait expression (at what scale - multiple scales?), suggests that trait shifts will occur with climate change

L363-364: This is a good example, but not clear as written (the link with water is not there). It may be more appropriate to say either that "soil moisture had a marked influence on the strength and direction of temperature-trait relationships in the tundra", or if you specifically want to limit to height, perhaps . "species become larger and large species are more prevalent at warmer and wetter sites in the tundra"

Thank you once again.
Haydn Thomas

Reviewer #2 (Remarks to the Author):

Manuscript: 'Climatic and soil factors explain the two-dimensional spectrum of global plant trait variation'

The authors address many comments and concerns presented in the review. They include more recent references and place their study in terms of what has been previously studied. They also extend their study beyond that of Diaz et al 2015. The new sections in the discussion along with the added section headers, makes it easier to follow and link directly to their findings.

I do not have any major comments but would like to suggest adding a comment in the discussion that points out the reduced sample sizes used for the woody, nonwoody comparison since this may result

in some of the observed differences.

Reviewer #3 (Remarks to the Author):

The authors have done a good job reviewing the manuscript. One very minor outstanding issue is that while the authors state the gap-filling method needs at least one observation per trait, table S3 page S44 of supplement shows two traits with 0 observations (seeds per reproductive unit and wood vessel element length), yet these traits have been included in the gap-filling and subsequent analyses. Please correct the table and/or correct the wording on the methods or please explain why traits with 0 observations have been used for the gap filling.

Minor comment
Supplementary needs some cleaning, pay particular attention to figures where legends are missing (e.g Fig S4, S14)

Our ref: NATECOLEVOL-210112692A

31st August 2021

Dear Dr. Joswig,

Thank you for your patience as we've prepared the guidelines for final submission of your Nature Ecology & Evolution manuscript, "Climatic and soil factors explain the two-dimensional spectrum of global plant trait variation" (NATECOLEVOL-210112692A). Please carefully follow the step-by-step instructions provided in the attached file, and add a response in each row of the table to indicate the changes that you have made. Please also check and comment on any additional marked-up edits we have proposed within the text. Ensuring that each point is addressed will help to ensure that your revised manuscript can be swiftly handed over to our production team.

We would like to start working on your revised paper, with all of the requested files and forms, as soon as possible (preferably within two weeks). Please get in contact with us immediately if you anticipate it taking more than two weeks to submit these revised files.

When you upload your final materials, please include a point-by-point response to any remaining reviewer comments.

If you have not done so already, please alert us to any related manuscripts from your group that are under consideration or in press at other journals, or are being written up for submission to other journals (see: https://www.nature.com/nature-research/editorial-policies/plagiarism#policy-on-duplicate-publication for details).

In recognition of the time and expertise our reviewers provide to Nature Ecology & Evolution’s editorial process, we would like to formally acknowledge their contribution to the external peer review of your manuscript entitled "Climatic and soil factors explain the two-dimensional spectrum of global plant trait variation". For those reviewers who give their assent, we will be publishing their names alongside the published article.

Nature Ecology & Evolution offers a Transparent Peer Review option for new original research manuscripts submitted after December 1st, 2019. As part of this initiative, we encourage our authors to support increased transparency into the peer review process by agreeing to have the reviewer comments, author rebuttal letters, and editorial decision letters published as a Supplementary item. When you submit your final files please clearly state in your cover letter whether or not you would like to participate in this initiative. Please note that failure to state your preference will result in delays in accepting your manuscript for publication.

Cover suggestions

As you prepare your final files we encourage you to consider whether you have any images or illustrations that may be appropriate for use on the cover of Nature Ecology & Evolution.

Covers should be both aesthetically appealing and scientifically relevant, and should be supplied at the best quality available. Due to the prominence of these images, we do not generally select images featuring faces, children, text, graphs, schematic drawings, or collages on our covers.

We accept TIFF, JPEG, PNG or PSD file formats (a layered PSD file would be ideal), and the image should be at least 300ppi resolution (preferably 600-1200 ppi), in CMYK colour mode.

If your image is selected, we may also use it on the journal website as a banner image, and may need to make artistic alterations to fit our journal style.

Please submit your suggestions, clearly labeled, along with your final files. We’ll be in touch if more information is needed.

Nature Ecology & Evolution has now transitioned to a unified Rights Collection system which will allow our Author Services team to quickly and easily collect the rights and permissions required to publish your work. Approximately 10 days after your paper is formally accepted, you will receive an email in providing you with a link to complete the grant of rights. If your paper is eligible for Open Access, our Author Services team will also be in touch regarding any additional information that may be required to arrange payment for your article.

Please note that <i>Nature Ecology & Evolution</i> is a Transformative Journal (TJ). Authors may publish their research with us through the traditional subscription access route or make their paper

immediately open access through payment of an article-processing charge (APC). Authors will not be required to make a final decision about access to their article until it has been accepted. Find out more about Transformative Journals

<B>Authors may need to take specific actions to achieve compliance with funder and institutional open access mandates. For submissions from January 2021, if your research is supported by a funder that requires immediate open access (e.g. according to Plan S principles) then you should select the gold OA route, and we will direct you to the compliant route where possible. For authors selecting the subscription publication route our standard licensing terms will need to be accepted, including our self-archiving policies. Those standard licensing terms will supersede any other terms that the author or any third party may assert apply to any version of the manuscript.

Please note that you will not receive your proofs until the publishing agreement has been received through our system.

For information regarding our different publishing models please see our Transformative Journals page. If you have any questions about costs, Open Access requirements, or our legal forms, please contact ASJournals@springernature.com.

Please use the following link for uploading these materials:
[REDACTED]

If you have any further questions, please feel free to contact me.

[REDACTED]

Reviewer #1:
Remarks to the Author:
Thank you for the careful and thoughtful review. I am satisfied that you have addressed my previous concerns, and I believe that the manuscript now reads more clearly, with greater potential impact on the field.

I have no further major comments, but have included a small number of minor suggestions below that may add additional clarity.

L149-156: This new phrasing is helpful, and I agree with your approach. For slight additional clarity I might suggest you break up this into two sentences e.g.

Combining the insights that the global spectrum of plant traits reveals two internally correlated, orthogonal groups, and that many plant traits are individually linked to environmental gradients. We would therefore expect that global patterns of trait correlation should closely follow gradients of climate and soil properties.

L156-158: Given the rephrased 'hypothesis' (expectation) statement, I am no longer sure these sub-questions are needed. Although previously I highlighted them as a good example of a clear hypothesis statement, given the new approach they slightly weaken the previous statement. It is also not immediately clear to the reader why you would need to determine those two questions in order to answer your overarching question. Instead, the reason for answering them comes through clearly enough later on in the text - to test a larger subset of traits (169-172), and to test whether relationships are different for woody vs non-woody species (189-191. I thought this was particularly clear!). I would therefore suggest you drop lines 156-158.

L223-224: I think I understand what you are getting at here, but this sentence could be overlooked as not particularly meaningful. Would it be helpful to add something like "..., as opposed to trait syndromes being defined by single environmental variables in isolation'

L264: Bjorkman rather than Bjørkman

L268-276: This paragraph is valid and important, but feels slightly out of place here. Is there scope to move to the methods or after line 168?

L328-329: This line does not make sense out of context. I suggest it needs qualifying in the context of the study e.g. "...by the environmental variables considered in this study".

L333: 'likely because economics traits vary within one plant' - so do some size traits (e.g. leaf area, leaf mass, seed mass). Suggest you add 'vary more than size traits' or something to that effect.

L359-360: This line is unclear, please could you rephrase. (e.g. Yet, the ubiquitous importance of climate variables for explaining current differences in trait expression (at what scale - multiple scales?), suggests that trait shifts will occur with climate change

L363-364: This is a good example, but not clear as written (the link with water is not there). It may be more appropriate to say either that "soil moisture had a marked influence on the strength and direction of temperature-trait relationships in the tundra", or if you specifically want to limit to height, perhaps . "species become larger and large species are more prevalent at warmer and wetter sites in the tundra"

Thank you once again.
Haydn Thomas

Reviewer #2:
Remarks to the Author:
Manuscript: 'Climatic and soil factors explain the two-dimensional spectrum of global plant trait variation'

The authors address many comments and concerns presented in the review. They include more recent references and place their study in terms of what has been previously studied. They also extend their study beyond that of Diaz et al 2015. The new sections in the discussion along with the added section headers, makes it easier to follow and link directly to their findings.

I do not have any major comments but would like to suggest adding a comment in the discussion that points out the reduced sample sizes used for the woody, nonwoody comparison since this may result in some of the observed differences.

Reviewer #3:
Remarks to the Author:
The authors have done a good job reviewing the manuscript. One very minor outstanding issue is that while the authors state the gap-filling method needs at least one observation per trait, table S3 page S44 of supplement shows two traits with 0 observations (seeds per reproductive unit and wood vessel element length), yet these traits have been included in the gap-filling and subsequent analyses. Please correct the table and/or correct the wording on the methods or please explain why traits with 0 observations have been used for the gap filling.

Minor comment
Supplementary needs some cleaning, pay particular attention to figures where legends are missing (e.g Fig S4, S14)

Author Rebuttal, first revision:

Answer to reviewers

Reviewer #1 (Remarks to the Author):

Thank you for the careful and thoughtful review. I am satisfied that you have addressed my previous concerns, and I believe that the manuscript now reads more clearly, with greater potential impact on the field.
We thank this reviewer for constructive comments, helping to substantially improve the manuscript.

I have no further major comments, but have included a small number of minor suggestions below that may add additional clarity.

L149-156: This new phrasing is helpful, and I agree with your approach. For slight additional clarity I might suggest you break up this into two sentences e.g.

Combining the insights that the global spectrum of plant traits reveals two internally correlated, orthogonal groups, and that many plant traits are individually linked to environmental gradients. We would therefore expect that global patterns of trait correlation should closely follow gradients of climate and soil properties.
Thank you. We changed it accordingly.

L156-158: Given the rephrased 'hypothesis' (expectation) statement, I am no longer sure these sub-questions are needed. Although previously I highlighted them as a good example of a clear hypothesis statement, given the new approach they slightly weaken the previous statement. It is also not immediately clear to the reader why you would need to determine those two questions in order to answer your overarching question. Instead, the reason for answering them comes through clearly enough later on in the text - to test a larger subset of traits (169-172), and to test whether relationships are different for woody vs non-woody species (189-191. I thought this was particularly clear!). I would therefore suggest you drop lines 156-158.
Thank you. We dropped these lines accordingly.

L223-224: I think I understand what you are getting at here, but this sentence could be overlooked as not particularly meaningful. Would it be helpful to add something like "..., as opposed to trait syndromes being defined by single environmental variables in isolation'
Thank you. We added this line accordingly.

L264: Bjorkman rather than Bjørkman
Thank you. We have made this change.

L268-276: This paragraph is valid and important, but feels slightly out of place here. Is there scope to move to the methods or after line 168?
Thank you. We agree with this shift and changed the position of this paragraph.

L328-329: This line does not make sense out of context. I suggest it needs qualifying in the context of the study e.g. "...by the environmental variables considered in this study".
Thank you. We added the suggested phrasing.

L333: 'likely because economics traits vary within one plant' - so do some size traits (e.g. leaf area, leaf mass, seed mass). Suggest you add 'vary more than size traits' or something to that effect.
We agree, and have made this change.

L359-360: This line is unclear, please could you rephrase. (e.g. Yet, the ubiquitous importance of climate variables for explaining current differences in trait expression (at what scale - multiple scales?), suggests that trait shifts will occur with climate change
We have rephrased and clarified that we refer to the ecoregion scale.

L363-364: This is a good example, but not clear as written (the link with water is not there). It may be more appropriate to say either that "soil moisture had a marked influence on the strength and direction of temperature-trait relationships in the tundra", or if you specifically want to limit to height, perhaps "species become larger and large species are more prevalent at warmer and wetter sites in the tundra"
Thank you for this clarification and suggestion. We have included it.

Thank you once again.
Haydn Thomas

Reviewer #2 (Remarks to the Author):

Manuscript: 'Climatic and soil factors explain the two-dimensional spectrum of global plant trait variation'

The authors address many comments and concerns presented in the review. They include more recent references and place their study in terms of what has been previously studied. They also extend their study beyond that of Diaz et al 2015. The new sections in the discussion along with the added section headers, makes it easier to follow and link directly to their findings.

I do not have any major comments but would like to suggest adding a comment in the discussion that points out the reduced sample sizes used for the woody, nonwoody comparison since this may result in some of the observed differences.

Thank you very much for your positive evaluation. We included your suggestion.

> *Lines 255-257: "However, we showed that these two main trait groups remain clearly identifiable when the analysis is conducted separately, **yet with fewer samples,** for woody and non-woody species (see Figures S3 to S5)."*

Reviewer #3 (Remarks to the Author):

The authors have done a good job reviewing the manuscript. One very minor outstanding issue is that while the authors state the gap-filling method needs at least one observation per trait, table S3 page S44 of supplement shows two traits with 0 observations (seeds per reproductive unit and wood vessel element length), yet these traits have been included in the gap-filling and subsequent analyses. Please correct the table and/or correct the wording on the methods or please explain why traits with 0 observations have been used for the gap filling.
Thank you very much for your positive evaluation. Regarding the question on gap filling: Table S3 indicates the data set derived from the large gap-filled data set. The data set used for gap-filling is described in Table S4. Both tables S3 and S4 have clarified table captions now. In the case of seeds per reproduction unit and vessel element length the observed number of values in the used data set is indeed 0, but was 10,083 and 12,455 respectively in the data set used for gap-filling.

> *Table S3: **Table describing the** trait information **of the data on 17 traits used for this study. This data has been extracted from a BHPMF gap-filled version of a larger trait set data (Table S4). The table includes the original** trait name and ID as used in the TRY data set (try-db.org), the abbreviation as used in this study, trait units, **and the original** number of observations in **the data prior to gap-filling.***

> *Table S4: **Trait information of the trait data set used for BHPMF gap-filling (for details see methods). This data set contains observed values of** 172 traits and 652,957 individuals. The table includes trait name and number of individual samples. **Traits that entered the analysis of this study appear in bold and in Table S3.***

Minor comment
Supplementary needs some cleaning, pay particular attention to figures where legends are missing (e.g Fig S4, S14)

Thank you. We cleaned some parts of the texts in the supplement, i.e. some figure and table captions, as well as adding some missing quotes Additionally we now explain for Figure S4 and S14 the colour coding of the traits in the caption.

> *S4, added: "**Trait row box colored according to the trait group (blue=size, red=economics, orange=other).**"*

5

*S14, added: “**Trait row box colored according to the trait group (blue=size, red=economics, orange=other).**”*

Our flexible approach during the COVID-19 pandemic
If you need more time at any stage of the peer-review process, please do let us know. While our systems will continue to remind you of the original timelines, we aim to be as flexible as possible during the current pandemic.

This email has been sent through the Springer Nature Tracking System NY-610A-NPG&MTS

Confidentiality Statement:

This e-mail is confidential and subject to copyright. Any unauthorised use or disclosure of its contents is prohibited. If you have received this email in error please notify our Manuscript Tracking Tracking System Helpdesk team at http://platformsupport.nature.com .

Details of the confidentiality and pre-publicity policy may be found here http://www.nature.com/authors/policies/confidentiality.html

Privacy Policy | Update Profile

DISCLAIMER: This e-mail is confidential and should not be used by anyone who is not the original intended recipient. If you have received this e-mail in error please inform the sender and delete it from your mailbox or any other storage mechanism. Springer Nature America, Inc. does not accept liability for any statements made which are clearly the sender's own and not expressly made on behalf of Springer Nature America, Inc. or one of their agents.

Please note that neither Springer Nature America, Inc. or any of its agents accept any responsibility for viruses that may be contained in this e-mail or its attachments and it is your responsibility to scan the e-mail and attachments (if any).

Final Decision Letter:

10th November 2021

Dear Ms Joswig,

I am writting in the temporary absence of my colleague, Dr. Alexa McKay.

We are pleased to inform you that your Article entitled "Climatic and soil factors explain the two-dimensional spectrum of global plant trait variation", has now been accepted for publication in Nature Ecology & Evolution.

Before your manuscript is typeset, we will edit the text to ensure it conforms to house style.

Once your manuscript is typeset you will receive a link to your electronic proof via email, with a request to make any corrections as soon as possible. If you have queries at any point during the production process then please contact the production team at rjsproduction@springernature.com. Once your paper has been scheduled for online publication, the Nature press office will be in touch to confirm the details.

Acceptance of your manuscript is conditional on all authors' agreement with our publication policies (see www.nature.com/authors/policies/index.html). In particular your manuscript must not be published elsewhere and there must be no announcement of the work to any media outlet until the publication date (the day on which it is uploaded onto our web site).

Please note that <i>Nature Ecology & Evolution</i> is a Transformative Journal (TJ). Authors may publish their research with us through the traditional subscription access route or make their paper immediately open access through payment of an article-processing charge (APC). Authors will not be required to make a final decision about access to their article until it has been accepted. Find out more about Transformative Journals

<B>Authors may need to take specific actions to achieve compliance with funder and institutional open access mandates. For submissions from January 2021, if your research is supported by a funder that requires immediate open access (e.g. according to Plan S principles) then you should select the gold OA route, and we will direct you to the compliant route where possible. For authors selecting the subscription publication route our standard licensing terms will need to be accepted, including our self-archiving policies. Those standard licensing terms will

supersede any other terms that the author or any third party may assert apply to any version of the manuscript.

In approximately 10 business days you will receive an email with a link to choose the appropriate publishing options for your paper and our Author Services team will be in touch regarding any additional information that may be required.

You will not receive your proofs until the publishing agreement has been received through our system.

If you have any questions about our publishing options, costs, Open Access requirements, or our legal forms, please contact ASJournals@springernature.com

An online order form for reprints of your paper is available at https://www.nature.com/reprints/author-reprints.html. All co-authors, authors' institutions and authors' funding agencies can order reprints using the form appropriate to their geographical region.

We welcome the submission of potential cover material (including a short caption of around 40 words) related to your manuscript; suggestions should be sent to Nature Ecology & Evolution as electronic files (the image should be 300 dpi at 210 x 297 mm in either TIFF or JPEG format). Please note that such pictures should be selected more for their aesthetic appeal than for their scientific content, and that colour images work better than black and white or grayscale images. Please do not try to design a cover with the Nature Ecology & Evolution logo etc., and please do not submit composites of images related to your work. I am sure you will understand that we cannot make any promise as to whether any of your suggestions might be selected for the cover of the journal.

You can now use a single sign-on for all your accounts, view the status of all your manuscript submissions and reviews, access usage statistics for your published articles and download a record of your refereeing activity for the Nature journals.

To assist our authors in disseminating their research to the broader community, our SharedIt initiative provides you with a unique shareable link that will allow anyone (with or without a subscription) to read the published article. Recipients of the link with a subscription will also be able to download and print the PDF.

You can generate the link yourself when you receive your article DOI by entering it here: <a href="http://authors.springernature.com/share">http://authors.springernature.com/share<a>.

[REDACTED]

P.S. Click on the following link if you would like to recommend Nature Ecology & Evolution to your librarian http://www.nature.com/subscriptions/recommend.html#forms

** Visit the Springer Nature Editorial and Publishing website at www.springernature.com/editorial-and-publishing-jobs for more information

about our career opportunities. If you have any questions please click here.**
